# Space‐Confined Electrocatalysis for Water Splitting: Synthesis, Performances, and Reaction Mechanism of Nanomaterials Toward High‐Performance Catalysis

**DOI:** 10.1002/anie.202510651

**Published:** 2025-08-21

**Authors:** Feiwu Zhang, Siyuan Niu, Yuxin Zhao, Changqing Li, Zhongping Li, Siliu Lyu, Yang Hou, Jong‐Beom Baek

**Affiliations:** ^1^ Hubei Key Laboratory of Automotive Power Train and Electronics School of Automotive Engineering Hubei University of Automotive Technology Shiyan 442002 China; ^2^ Hubei Key Laboratory of Energy Storage and Power Battery School of Mathematics Physics and Optoelectronic, Engineering Hubei University of Automotive Technology Shiyan 442002 China; ^3^ School of Energy and Chemical Engineering/Center for Dimension Controllable Organic Frameworks Ulsan National Institute of Science and Technology 50 UNIST Ulsan 44919 South Korea; ^4^ Key Laboratory of Biomass Chemical Engineering of Ministry of Education College of Chemical and Biological Engineering Zhejiang University Hangzhou 310027 China; ^5^ Key Laboratory of Automobile Materials of MOE and School of Materials Science and Engineering Jilin University Changchun 130012 P. R. China

**Keywords:** Electrocatalysts, Hydrogen evolution reaction, Overall water splitting, Oxygen evolution reaction, Space‐confined catalysis

## Abstract

Water splitting represents a sustainable and environmentally benign approach for green hydrogen generation and future clean energy solutions. In this context, space‐confined synthesis has emerged as a powerful strategy for engineering high‐performance electrocatalysts. Recent studies have demonstrated that low‐dimensional nanomaterials synthesized via confinement techniques exhibit enhanced electrocatalytic properties. The confined microenvironment imparts superior electrical conductivity, structural stability, and active site accessibility, while also facilitating elevated catalytic activity and offering potential for scalable production in practical energy applications. In this review, we first present mechanistic insights into nanoconfinement‐enhanced water splitting electrocatalysis and characterization techniques including in‐situ/*operando* analysis for confined electrocatalysts, emphasizing how the confined architectures from one to three dimensions (1D‐3D) regulate electronic structures, facilitate reactant adsorption, and reduce energy barriers. We then outline nanoscale confinement strategies, including in‐situ and postsynthetic approaches using diverse host materials such as carbon nanotubes (CNTs), metal–organic frameworks (MOFs), and MXenes, along with advanced methods for controlling particle dispersion and size. Next, we summarize recent progress in confined electrocatalysts for water splitting, highlighting density functional theory (DFT)‐guided design and structure–property relationships. Finally, we address current challenges and future opportunities in synthesis control, in‐situ characterization, and scalable deployment for practical hydrogen production.

## Graphical abstract

Schematic representation of dimensionally confined nanocatalysts (1D to 3D) for electrocatalytic water splitting, which represents a sustainable route for green hydrogen generation. Space‐confined synthesis has emerged as a potential strategy for constructing advanced electrocatalysts. The graphic represents the space‐confined nanomaterials within 1D to 3D frameworks and their roles in enhancing HER, OER, and overall water splitting performance, with emphasis on the associated catalytic mechanisms.

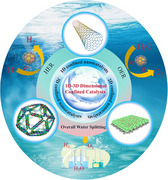

## 1 Introduction

Growing environmental problems and increasing climate change have created urgent demand for clean and renewable energy sources.^[^ ^1^, ^2^ ^]^ Alternative approaches to reduce dependence on fossil fuels are being developed, including some based on nuclear energy, wind energy, and hydrogen energy.^[^ ^3^, ^4^ ^]^ Among them, hydrogen has been identified as a promising substitute for traditional fossil energy sources because of its advantageous properties, including good thermal conductivity, high energy density, and high environmental compatibility.^[^ ^5^ ^]^ Currently, Pt and RuO_2_/IrO_2_ are the state‐of‐the‐art electrocatalysts for hydrogen evolution reaction (HER) and oxygen evolution reaction (OER), respectively. However, their limited abundance, poor durability, and high cost render them unsuitable for large‐scale industrial use.^[^ ^6^, ^7^ ^]^ Consequently, extensive research in recent decades has focused on developing earth‐abundant nonprecious electrocatalysts and nonmetal catalysts to reduce noble metal usage while maintaining sufficient activity. However, these alternatives typically exhibit lower activity and stability than precious metal‐based materials, highlighting the significant challenges in achieving efficient and durable catalysts.^[^ ^8^, ^9^ ^]^

Confining catalytic materials within nanoconfined structures significantly alters the physical and chemical properties of guest materials or composites while stimulating interactions between trapped molecules/particles. Collectively, these effects define the nanoconfinement effect.^[^ ^10^, ^11^, ^12^ ^]^ Domain confinement strategies for nanoconfinement have emerged as a promising approach to enhance electrocatalytic performance.^[^ ^13^ ^]^ Downsizing nanoparticles to subnanometer or atomic scales has emerged as a promising strategy to markedly increase surface active sites and improve reactant accessibility for catalysts.^[^ ^14^ ^]^ Nanoscale confinement endows catalysts with key properties; i) atomic dispersion of active centers, maximizing reactant exposure; ii) coordinatively unsaturated sites featuring high reactivity; iii) electron confinement combined with quantum size effects to fine‐tune performance; and iv) intricate support interactions that modulate catalytic activity.^[^ ^15^, ^16^, ^17^, ^18^ ^]^ Considering these properties, nanoconfined catalysts can simultaneously enhance both intrinsic activity and selectivity in diverse electrochemical reactions, underscoring the potential of nanoscale confinement for optimizing catalytic systems.^[^ ^18^ ^]^

Notably, in recent years there has been rapid progress in the synthesis of catalysts of various dimensions and their application to electrochemical water splitting.^[^ ^19^ ^]^ A range of nanoconfined materials have been developed that demonstrate their promising electrocatalytic potential, including diverse dimensional structures [like 1D carbon nanotubes (CNTs),^[^ ^20^ ^]^ 2D graphene,^[^ ^21^ ^]^ and 3D metal–organic frameworks (MOFs)],^[^ ^22^, ^23^ ^]^ doped and functionalized nanoconfined materials, along with hybrid or nanostructured composites.^[^ ^24^ ^]^

To maximize active sites for reactants/intermediates and enhance water splitting performance, strategies including structural design, surface modification, and interface optimization have been developed.^[^ ^25^, ^26^ ^]^ Crafting and assembling structures with distinctive attributes can expand electrochemical surface areas and provide more active sites, thus enhancing their electrochemical properties.^[^ ^27^ ^]^ From 1D fullerene to 3D porous structures, these nanoconfined spaces of various dimensions can precisely control reaction pathways and efficiencies, providing ideal conditions for catalytic reactions.^[^ ^10^ ^]^

For example, 1D nanowires or nanotubes serve as ideal channels for efficient electron and proton transport, minimizing energy loss during electrocatalysis and enhancing the reaction kinetics of the HER.^[^ ^12^, ^28^, ^29^ ^]^ 2D materials, such as graphene, provide expansive active surfaces that expedite surface reactions for both the OER and the HER, while their distinctive electronic structures enhance electron conduction.^[^ ^30^ ^]^ 3D structures such as porous MOFs or porous carbon materials increase reaction areas and facilitate more effective mass and energy transfers, further enhancing the overall catalytic performance of water splitting.^[^ ^31^ ^]^ Such confined structures provide highly concentrated active sites and improved electron transport efficiency, greatly enhancing catalytic activity and selectivity while also strengthening the stability and durability of the catalysts.^[^ ^32^ ^]^

Additionally, multidimensional confinement strategies optimize reaction conditions by creating a stable chemical environment to protect catalysts and enable precise control over water electrolysis through tailored structural and compositional designs, thereby prolonging catalyst service life.^[^ ^32^, ^33^ ^]^

In this work, our review provides a comprehensive overview of nanoconfined electrocatalysts for water splitting, emphasizing their structural design, mechanistic understanding, and practical applications. We first introduce the fundamental concepts and classifications of spatial confinement and discuss its mechanistic roles in modulating water adsorption, electric fields, and ion diffusion. Recent advancements in in‐situ/*operando* techniques and density functional theory (DFT) simulations are highlighted for their ability to reveal atomic‐level structure–activity relationships. We then summarize various confinement strategies using host materials such as porous carbon, CNTs, MOFs, and MXenes, along with advanced synthesis methods that improve dispersion and control. Special attention is given to how confinement alters electronic structures, stabilizes intermediates, and lowers energy barriers in HER and OER. A systematic summary of confined systems (1D–3D) is provided, linking dimensionality to performance. Finally, we discuss current challenges and future directions, including mass transport limitations, scalability, and the integration of theory and experiment for rational catalyst design.

## 2 Mechanistic Insights and Characterization Techniques for Water Splitting Electrocatalysis

### 2.1 Concept and Classification of Nanoconfinement

Nanoconfinement refers to the phenomenon where materials or molecules are confined within nanoscale spaces, resulting in significant changes to their physical and chemical properties.^[^ ^34^ ^]^ Specifically, this effect typically occurs when active sites are constrained within nanoscale channels, cavities, or interfaces, thereby altering the mass transport of reactants, the electronic structure of catalysts, and their interactions with reactants. Compared to traditional bulk materials, nanoconfinement significantly influences the reaction kinetics, selectivity, and stability of catalytic reactions, making it an important method for optimizing material performance in catalytic processes.^[^ ^35^ ^]^

The core of nanoconfinement is physical confinement, where the active sites of the catalyst are restricted within nanoscale spaces, limiting reactants to interact only within this confined space. This spatial restriction can effectively control the distribution of reactants and the reaction pathways.^[^ ^36^ ^]^ As a result, the energy barriers of the reaction may decrease, reaction rates may accelerate, or the product selectivity may change. Nanoconfinement is not merely a spatial dimension limitation but also involves electronic effects, which refer to changes in the electron density distribution within the confined space. These changes can alter the surface electronic structure of the catalyst, thereby affecting its catalytic performance.^[^ ^36^, ^37^, ^38^ ^]^ Furthermore, nanoconfinement also influences the water splitting reaction by altering the structure, thermodynamic behavior, and dynamic processes of water. Compared to bulk water, water in confined spaces exhibits distinct hydrogen bonding structures, thermodynamic properties, and dynamic behaviors, which further reduce the energy barriers of reactions and enhance both the efficiency and selectivity of the reaction.^[^ ^36^, ^39^ ^]^

This review introduces the applications of nanoconfinement in water splitting from the perspective of the dimensionality of the confined spaces, categorized into 1D, 2D, and 3D confinement: 1D nanoconfinement refers to this type of confinement occurring in linear structures, such as nanopores, where materials are restricted along a single dimension, influencing the transport and reactivity of molecules within that space.^[^ ^34^ ^]^ 2D nanoconfinement refers to confinement occurring at interfaces or in layered structures, restricted to two dimensions. Typical examples include thin films and certain nanostructures, where molecules are confined between two closely spaced surfaces. 3D nanoconfinement is the most comprehensive form of confinement, where molecules or reactants are completely enclosed within a 3D space, such as nanopores or cavities.^[^ ^35^, ^40^ ^]^ This type of confinement provides a fully restricted environment, altering the way molecules interact and react. Understanding these different types of nanoconfinement and their effects helps in developing materials and catalysts with higher performance, particularly in processes such as water splitting, where improving reaction kinetics and product selectivity is crucial.

### 2.2 Fundamental Principles of Nanoconfinement Effects on Water Splitting Catalysis

Nanoconfinement effects are essential in catalysis, particularly in water splitting reactions, where the confined space alters the interactions between reactants and catalysts, modifies the electronic structures of catalysts, and promotes the formation of unique reaction pathways. This section explores the mechanisms of nanoconfinement effects in water splitting catalysis, with a focus on 1D, 2D, and 3D materials that act as confined catalytic environments.^[^ ^36^ ^]^

The nanoconfinement effect in 1D CNTs has a significant impact on water splitting catalysis. For example, the reduction temperature of Fe_2_O_3_ nanoparticles supported on the exterior walls of CNTs is above 1070 K, while Fe_2_O_3_ nanoparticles confined within CNTs (with an inner diameter of approximately 8 nm) can be reduced at about 900 K. When the inner diameter of the CNTs is reduced to about 2 nm, the reduction temperature further decreases to approximately 860 K. This demonstrates that the confined space within CNTs significantly enhances catalytic activity by altering the redox properties of the embedded metal nanoparticles.^[^ ^32^ ^]^ The electronic interaction between the metal nanoparticles and the CNT walls causes a downshift in the metal's d‐band states, which weakens the adsorption energy of oxygen, thus promoting key reduction reactions in the water splitting process. To quantify this effect, the concept of confinement energy (*E*_con_) is introduced, defined as the difference in the adsorption energy of reaction intermediates on confined and nonconfined catalysts. A positive *E*_con_ value indicates that the adsorption of reactants/intermediates is weakened in the confined space, suggesting that reactants are less likely to bind too strongly with the catalyst surface in the confined space. This effect can be validated through surface science experiments, such as adsorption energy measurements. Additionally, this encapsulated structure not only improves catalytic efficiency but also significantly enhances the stability of the catalyst by preventing sintering and agglomeration of the active components. By precisely controlling the inner diameter and crystallinity of CNTs, the confinement effect can be further optimized to achieve milder and more efficient water splitting conditions.^[^ ^32^, ^41^ ^]^

In 2D materials, as a fundamental nanoconfinement catalyst scaffold, MOFs are central to the advancement of water splitting processes. Studies have shown that the adsorption of atoms and molecules on metal surfaces within the pores of MOFs is enhanced due to the geometric constraints and confinement field created by the MOF. The geometric constraints and confinement effect of MOFs alter the electronic state of the metal active sites, lowering the energy barrier for the dissociation of water molecules on the catalyst surface, thereby enhancing catalytic efficiency for water splitting.^[^ ^42^ ^]^ The MOF not only alters the electronic structure of the metal active sites but also adjusts the interaction between water molecules and the metal active sites, making it easier for water molecules to dissociate in the confined space, generating hydrogen and oxygen species, and further promoting the water splitting reaction. The strength of the confinement effect is closely related to the interactions between the walls of the confined space and the metal active sites. The use of different MOFs with varying pore sizes, shapes, and chemical compositions can also modify the local potential distribution, thereby adjusting the catalytic performance of water splitting reactions. By carefully selecting and designing MOFs, catalytic performance can be fine‐tuned, providing a more efficient catalytic environment for water splitting reactions.^[^ ^42^, ^43^ ^]^

3D nanoconfinement structures, such as MOFs, COFs, and porous metal oxides, offer unique microenvironments for water splitting reactions, thanks to their highly ordered porous structures and tunable pore sizes and chemical compositions. The pore size plays a crucial role in 3D nanoconfinement structures.^[^ ^32^, ^41^, ^43^ ^]^ By precisely controlling the types and connections of metal nodes and organic ligands, the pore size can be finely tuned, enabling molecular sieving of water molecules, selectively allowing specific sizes of water molecules to enter active sites while blocking larger impurities or by‐product intermediates. The chemical composition of 3D nanoconfinement structures also significantly affects water splitting reactions. The specific interaction between metal centers and water molecules can lower the dissociation energy barrier for water, while functional groups can stabilize the transition state of water molecules through noncovalent interactions, such as hydrogen bonding, further promoting the water dissociation process. Moreover, factors such as the shape of the pores, surface charge distribution, and internal chemical potential profoundly influence the catalytic process.^[^ ^43^ ^]^ By adjusting synthesis conditions, postsynthesis modifications, and composite strategies, catalytic performance can be finely tuned, especially by combining 3D nanoconfinement materials with other cocatalysts or carriers, which can enhance electron transport efficiency and stability, further improving the catalyst's activity and stability. The surface charge and polarity effects of 3D nanoconfinement structures also offer potential advantages. Through electrostatic interactions and the polarization effects of water molecules, water molecules can be more readily adsorbed, activated, and dissociated, thereby enhancing the efficiency of the water splitting reaction.^[^ ^41^, ^43^, ^44^ ^]^ Through the integrated control of 1D, 2D, and 3D nanoconfinement effects, more efficient catalytic systems for water splitting reactions can be designed. These materials not only optimize the electronic structure of catalysts but also improve the efficiency, stability, and selectivity of the water splitting reaction by adjusting the adsorption and conversion pathways of reactants.^[^ ^42^ ^]^ This provides important theoretical guidance for the development of catalysts for sustainable hydrogen production.

### 2.3 Characterization Techniques for Space‐confined Electrocatalysts

Physicochemical characterization is crucial for understanding confined electrocatalysts by revealing active site structures and guiding rational design for water splitting. Techniques such as scanning electron microscopy (SEM), transmission electron microscopy (TEM), and high‐angle annular dark‐field scanning transmission electron microscopy (HAADF‐STEM) visualize nanoscale confinement, while synchrotron‐based X‐ray absorption spectroscopy (XAS) including X‐ray absorption near‐edge structure (XANES) and extended X‐ray absorption fine structure (EXAFS) elucidates chemical states and coordination environments. DFT simulations offer atomic‐level insights into structure–activity relationships. Advancements in in‐situ/*operando* spectroscopy further enable real‐time monitoring of active site evolution and catalytic mechanisms under working conditions.

#### 2.3.1 Electron Microscopy Characterization

Catalyst morphology is typically analyzed using scanning electron microscopy (SEM) and transmission electron microscopy (TEM), though these techniques offer only microscale resolution.^[^ ^45^ ^]^ To resolve atomic structures in confined catalysts, more advanced imaging is necessary. Scanning transmission electron microscopy (STEM), enhanced with scanning coils and detectors, enables compositional and bonding analysis at buried interfaces. Annular dark‐field (ADF) imaging via STEM is effective for visualizing heavy metal atoms in thin microenvironments.^[^ ^18^ ^]^ High‐angle annular dark‐field STEM (HAADF‐STEM) further improves atomic‐scale imaging by reducing structure‐related artifacts. Coupling HAADF‐STEM with electron energy‐loss spectroscopy (EELS) allows for simultaneous elemental analysis.^[^ ^46^ ^]^ To visualize light atoms and active sites, aberration‐corrected STEM (AC‐STEM) provides real‐space imaging and chemical information such as bonding states and atomic valence. Scanning tunneling microscopy (STM) is well‐suited for examining 2D confined systems, while modified atomic force microscopy (AFM), particularly in noncontact mode, achieves high‐resolution 3D imaging in air or liquid‐independent of sample conductivity‐thus complementing STM's limitations.^[^ ^47^ ^]^

#### 2.3.2 Spectroscopic Methods Based on X‐rays

Compared with microscopic techniques, X‐ray‐based spectroscopic methods offer superior elemental resolution, especially for elements with similar atomic numbers, due to their short wavelengths (0.01–1 nm).^[^ ^48^ ^]^ This enables atomic‐scale identification of active sites in confined catalysts. X‐ray diffraction (XRD) is commonly employed to determine crystalline structures of space‐confined electrocatalysts. X‐ray photoelectron spectroscopy (XPS) provides insights into surface elemental composition and oxidation states, though its limited sensitivity necessitates complementary analysis like inductively coupled plasma optical emission spectroscopy (ICP‐OES).^[^ ^49^ ^]^ For detailed atomic coordination, X‐ray absorption fine structure (XAFS), including extended X‐ray absorption fine structure (EXAFS) and X‐ray absorption near edge structure (XANES), is widely used. EXAFS reveals interatomic distances and coordination numbers, while XANES elucidates oxidation states, bond angles, and electronic structures, offering a deeper understanding of confined catalytic environments.^[^ ^50^ ^]^

#### 2.3.3 *In‐situ/Operando* Analysis of Confined Systems

Raman spectroscopy, with its high specificity and sensitivity in the low‐frequency region, is particularly advantageous for in‐situ monitoring of space‐confined electrocatalytic water splitting. Coupling Raman spectroscopy with electrochemical techniques enables real‐time tracking of dynamic processes and provides valuable insight into the structure–activity relationships of confined catalysts. In particular, in‐situ/*operando* Raman spectroscopy facilitates the understanding of lifetime structural evolution and its correlation with catalytic performance.^[^ ^51^, ^52^ ^]^ The advancement of light sources with enhanced brightness and broader spectral output in confined environments has significantly improved the signal quality of in‐situ Fourier‐transform infrared spectroscopy (in‐situ FTIR). These improvements allow the detection of vibrational modes at the reaction interface, enabling molecular‐level identification of transient intermediates. The integration of customized in‐situ reaction cells further aids in elucidating mechanistic pathways within nanoconfined catalytic systems.^[^ ^53^ ^]^

In‐situ X‐ray diffraction (in‐situ XRD) offers real‐time observation of crystalline phase transitions under operational conditions.^[^ ^54^ ^]^ However, its limitations include low spatial resolution and insensitivity to surface or interfacial phenomena. Meanwhile, in‐situ X‐ray absorption spectroscopy (in‐situ XAS), particularly with synchrotron radiation, provides atomic‐level information on local coordination environments and electronic structure dynamics. This is critical for capturing catalyst reconstruction, electron redistribution, and intermediate formation during water oxidation.^[^ ^55^, ^56^ ^]^ Nevertheless, XAS largely reflects averaged structural data and may lack surface‐specific sensitivity, especially in heterogeneous or complex systems.

In‐situ electron microscopy techniques, such as environmental TEM,^[^ ^57^ ^]^ in‐situ STEM,^[^ ^58^ ^]^ scanning electrochemical cell microscopy (SECCM),^[^ ^59^ ^]^ and liquid‐phase transmission electron microscope (LP‐TEM),^[^ ^60^ ^]^ offer real‐time monitoring of structural and compositional evolution in confined electrocatalysts during water splitting. These approaches enable direct visualization of active site dynamics, particle migration, and morphological changes, providing mechanistic insights essential for the rational design of stable and high‐performance confined electrocatalysts.

### 2.4 Confined Effects in Water Splitting: DFT Insights

DFT calculations have been extensively employed to investigate the influence of confined effects on catalytic processes, revealing their critical roles in electronic structure modulation, reactant adsorption, and energy barrier reduction.^[^ ^61^, ^62^ ^]^ In this section, we combine the results from DFT calculations to provide a concise analysis of the application of confined effects in water splitting reactions, exploring their impact on reaction mechanisms.^[^ ^63^ ^]^

DFT calculations show that confined effects significantly enhance the adsorption and activation of water molecules by restricting their movement in nanoscopic spaces.^[^ ^64^ ^]^ Within confined structures, water molecules experience structural rearrangement of their hydrogen bond network, leading to stronger interactions with catalyst surfaces and accelerating the reaction between water molecules and catalysts. For instance, in Fe_2_O_3_‐loaded CNT catalysts, DFT calculations indicate that water molecules undergo structural rearrangements within the confined environment, enhancing adsorption and lowering the energy barrier required for water dissociation.^[^ ^65^ ^]^ The confined space also induces metal–support charge transfer and quantum effects, which shift the d‐band center of the catalyst and further optimize the electronic structure of active sites. Studies show that in Fe_2_O_3_ catalysts, confined effects lower the energy barrier for OER, making oxygen generation more efficient. These findings suggest that confined effects are highly advantageous in accelerating reaction rates and reducing energy barriers.^[^ ^65^, ^66^, ^67^ ^]^

Confined effects also enhance the catalytic efficiency of water splitting reactions by altering the electronic structure of the catalyst.^[^ ^67^, ^68^ ^]^ DFT calculations reveal that within confined spaces, the electronic density distribution of catalysts undergoes significant changes, primarily evidenced by a shift in the d‐band center of the catalyst surface. For example, in the case of graphene‐confined single‐atom Fe (FeN_4_) catalysts for methane oxidation, the confined environment shifts the d‐band center of Fe by 0.3 eV, enhancing the hybridization with O_2_ antibonding orbitals.^[^ ^67^ ^]^ This increases the adsorption energy of O_2_ and lowers the O─O bond dissociation energy. By modulating the electronic structure, confined effects enhance the electron transfer capability of reactants, which is particularly beneficial for OER, as it helps to reduce the energy barriers in the reaction. DFT calculations further reveal that confined effects reduce the energy barriers of water splitting reactions by optimizing the interaction between reactants and the catalyst surface. For instance, in HER, IrO_2_ catalysts within confined spaces enhance the adsorption of hydrogen intermediates, significantly lowering the energy required for the reaction. In OER, confined effects optimize the dissociation pathways of water molecules, thus reducing the energy requirements for the reaction. Confined effects significantly improve the kinetics and thermodynamics of water splitting reactions by altering reaction pathways, lowering energy barriers, and accelerating reaction rates. DFT calculations provide a detailed mechanism analysis, offering valuable theoretical insights for catalyst design.^[^ ^66^, ^67^, ^69^ ^]^

Confined effects significantly enhance the local concentration of reactants within nanoscopic channels, which in turn lowers the diffusion resistance of reactants, as demonstrated by DFT calculations. DFT studies show that spatial constraints within these channels improve the interaction between water molecules and catalyst surfaces, accelerating dissociation rates. For example, in CoFe_2_O_4_ catalysts, DFT simulations reveal that confined effects facilitate the diffusion of reactants to the catalyst surface, leading to a notable increase in the dissociation rate of water molecules. Moreover, DFT results indicate that confined environments stabilize reaction intermediates, such as oxygen species, during the OER, thus reducing the energy required for the reaction. Furthermore, DFT analysis suggests that confined effects optimize the stability of reaction intermediates by altering the electronic structure of the catalyst surface. In single‐atom catalysts, such as the graphene‐confined single‐atom Fe catalyst, DFT calculations show that confined effects cause shifts in the d‐band center of the catalyst, enhancing electron transfer between the catalyst and reactants. This shift increases O_2_ adsorption and lowers O─O dissociation energy, which, according to DFT, leads to improved methane oxidation efficiency.

Confined effects play a significant role in enhancing the efficiency of water splitting reactions by modulating the electronic structure of catalysts, optimizing the interaction between reactants and catalyst surfaces, reducing energy barriers, and accelerating reaction rates.^[^ ^61^, ^62^, ^70^, ^71^ ^]^ DFT calculations provide the theoretical foundation for understanding these mechanisms and offer valuable insights for designing novel, efficient catalysts. Future research may combine machine learning techniques and high‐throughput calculations to further optimize the design of confined structures, such as pore size and coordination environment of active sites. Additionally, research integrating multiphysics coupling models will enable more precise simulations of the synergistic effects of electric fields, strain fields, and confined effects on catalysis. These advances will not only help improve water splitting efficiency but also provide new material design concepts for energy catalysis, environmental remediation, and other applications.^[^ ^66^, ^69^, ^72^ ^]^

## 3 Postconfinement and In‐situ Confinement Strategies for Engineering Nanoscaled Spaces

Nanoconfinement is achieved by partially or completely enclosing materials within nanoscale structures such as pores, channels, 2D interfaces, pockets, or cavities.^[^ ^73^ ^]^ In nanoconfined synthesis, these confined spaces act as nanoreactors to regulate product size and morphology while providing tailored microenvironments that facilitate nucleation, growth, and stabilization.^[^ ^10^, ^73^ ^]^ Consequently, the confined spaces play a crucial role in the nanoconfinement process. They can be classified into three categories based on their dimensionality: 1D (longitudinal nanoconfined spaces), 2D (interfacial nanoconfined spaces), and 3D (porous nanoconfined spaces).

**Figure 1:**
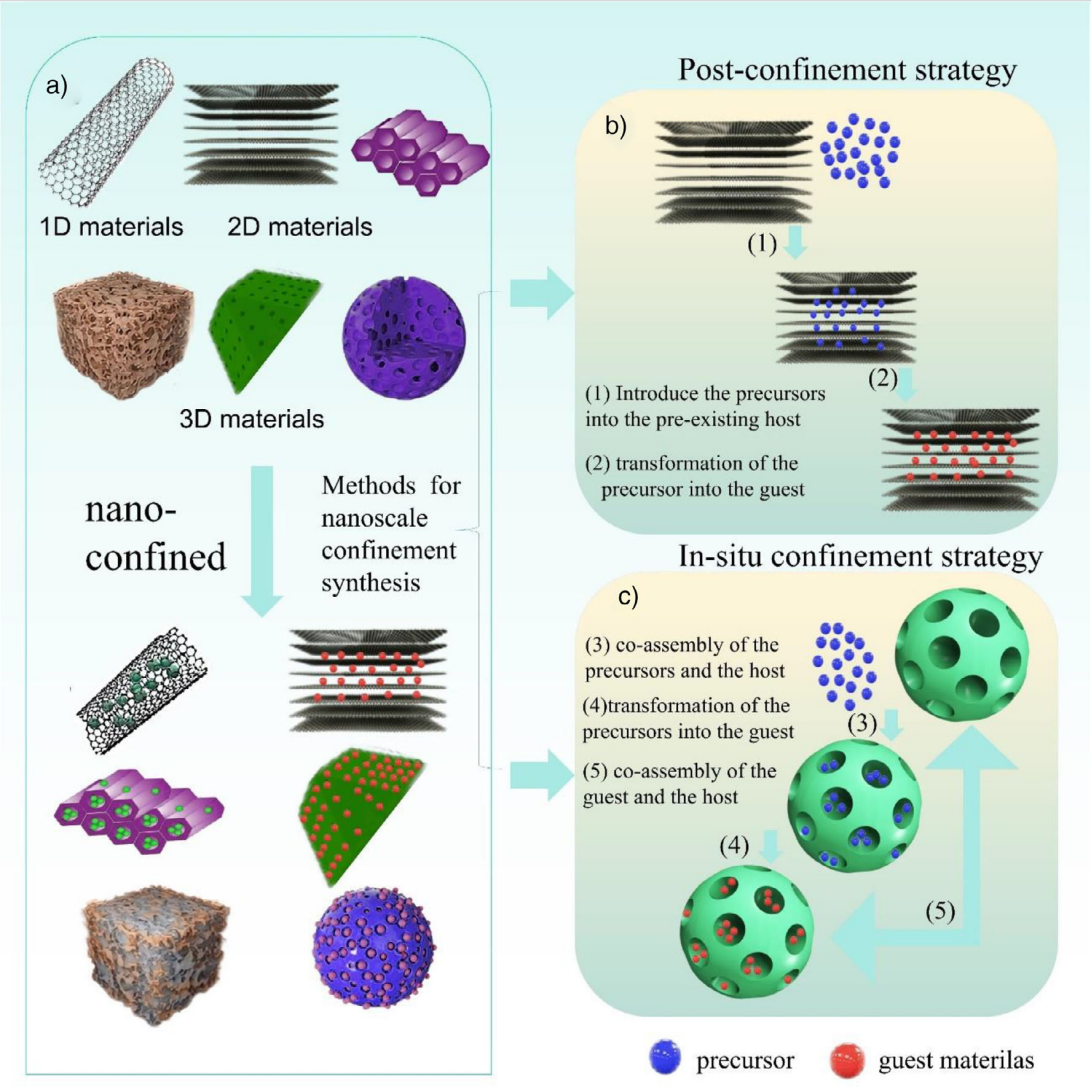
a) Host materials are categorized into different dimensions, reflecting their 1D, 2D, or 3D structures. b) and c) Nanoconfined synthesis is typically achieved through two predominant strategies: postconfinement and in‐situ confinement. These methods harness the unique properties of confined nanospaces to enhance the synthesis of materials.

Nanoconfined synthesis entails encapsulating reactants (termed “guests”) within the nanoscale spaces of host materials to create materials with tailored properties.^[^ ^10^, ^74^ ^]^ As illustrated in Figure 1, nanoconfined synthesis is classified into two primary strategies based on the preparation sequence of host and guest materials. The first, postconfinement strategy, involves introducing precursors into a preformed host and converting them into the guest. The second, in‐situ confinement strategy, entails simultaneous formation of both host and guest materials.^[^ ^75^ ^]^ The in‐situ strategy may also influence the direct coassembly of host and guest materials, or the combined assembly of their precursors, which then undergo transformation into the final guest material.^[^ ^10^, ^75^ ^]^

Frequently used host materials, often referred to as nanoreactors or nanoscaffolds,^[^ ^76^ ^]^ include carbon‐based materials,^[^ ^77^ ^]^ MOFs,^[^ ^78^ ^]^ MXenes,^[^ ^79^ ^]^ and porous organic polymers,^[^ ^80^ ^]^ that primarily consist of advantageous porous structures and tunable pore sizes. As discussed, nanoconfined spaces are classified into three types by confinement dimensions: 1D nanotubes, 2D interfaces, and 3D pores. Synthesizing materials within these structures represents the main approaches for constructing nanoconfined environments. In the following sections, we will systematically analyze how these two distinct confinement strategies influence catalytic performance within nanoreactors.

### 3.1 Post Confinement Strategy

The postconfinement strategy is a method for synthesizing nanomaterials within preformed confined spaces, such as porous materials and nanochannels. The core of this approach involves introducing precursors into these predefined confined spaces and then converting them into the desired materials via chemical reactions. This process leverages the confined space as a “nanoreactor”, enabling precise control over the synthesis of nanomaterials. One of the key advantages of the postconfinement strategy is its relative simplicity and ease of implementation.^[^ ^81^, ^82^ ^]^ It is applicable to a wide range of material systems, including MOFs, mesoporous silica, and carbon materials. By employing this strategy, researchers can precisely control the size and morphology of nanomaterials, thereby optimizing their properties. For instance, by adjusting the dimensions and shapes of the confined spaces, it is possible to synthesize nanoparticles with specific sizes and morphologies, which hold significant potential for applications in catalysis, adsorption, and sensing. Moreover, the postconfinement strategy allows for further optimization of the synthesis process by altering reaction conditions, such as temperature, pressure, and solvent. This flexibility is particularly beneficial for synthesizing nanomaterials with complex structures and functions, as it enables precise control at the nanoscale.^[^ ^83^, ^84^ ^]^

**Figure 2:**
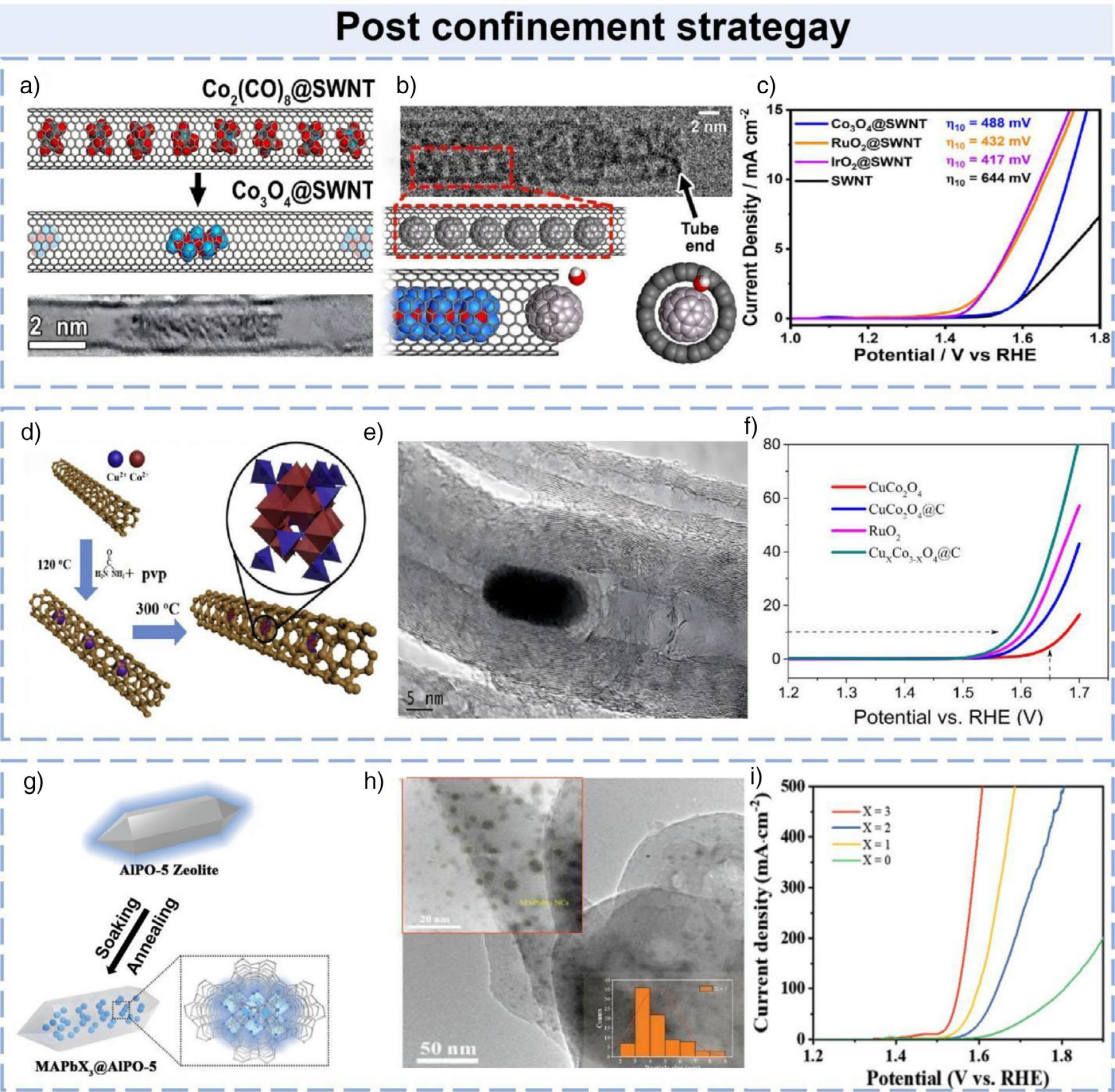
a) Co_2_(CO)_8_@SWNT to Co_3_O_4_@SWNT. b) HR‐TEM image in Panel b reveals the presence of fullerene filling at the ends of SWNTs within the C_60_‐plugged Co_3_O_4_@SWNT composite. c) RDE‐LSVs of 0.5 mg cm^−2^ Co_3_O_4_@SWNT, RuO_2_@SWNT, IrO_2_@SWNT, and empty SWNTs in O_2_‐saturated 0.1 M KOH. Test conditions: 1600 rpm, 5 mV s^−1^, 1.0–1.8 V versus RHE. Materials on GC disk electrode (graphite counter electrode). Currents normalized to disk area. Hg/HgO reference electrode; potentials converted to RHE scale. Reproduced fromRef.[85] Copyright 2025, with permission from Elsevier. d) Schematic synthesis of the Cu_X_Co_3‐X_O_4_@C nanocomposites. e) HRTEM image of the Cu_X_Co_3‐X_O_4_@C composites. f) OER polarization curves for CuCo_2_O_4_, CuCo_2_O_4_@C, Cu_x_Co_3‐x_O_4_@C, and RuO_2_ catalysts in O_2_‐saturated 0.1 M KOH. (Sweep rate: 5 mV s^−1^; rotation rate: 1600 rpm). Reproduced from Ref.[86] Copyright 2019, with permission from Elsevier. g) Schematic diagram of the synthesis procedure for MAPbX_3_@AlPO‐5 composite. h) Show a magnified region of the TEM image and the particle size distribution of MAPbBr_3_ nanocrystals (NCs), respectively. i) Polarization curves. Reproduced from Ref.[87] Copyright 2023, with permission from John Wiley and Sons.

In 2025, Townsend and colleagues reported a carbon‐coated catalyst comprising metal‐oxide nanoparticles encapsulated within single‐walled carbon nanotubes (SWNTs).^[^ ^85^ ^]^ This study investigated the effects of carbon coatings on catalytic performance by encapsulating metal oxides within SWNTs. (Figure 2a) depicts the encapsulation of Co_2_(CO)_8_ within SWNTs and its subsequent oxidation to form Co_3_O_4_@SWNT, achieving effective confinement of metal‐oxide nanoparticles. HRTEM images (Figure 2b) confirm direct contact between the nanoparticles and SWNT sidewalls, facilitating electron transfer. Electrochemical tests (Figure 2c) reveal that the significant enhancement of carbon‐based catalysts' electrocatalytic activity through metal‐oxide encapsulation within SWNTs, aligning with the postconfinement strategy. This strategy allows for precise control of catalyst distribution and electronic properties, thereby enhancing catalytic activity and stability. In this study, encapsulating metal‐oxide nanoparticles within SWNTs not only stabilizes the catalyst but also significantly improves its OER catalytic activity through electron transfer between the carbon shell and the metal oxide. By encapsulating metal‐oxide nanoparticles within SWNTs, the catalyst achieves enhanced stability while simultaneously improving OER performance through optimized electron transfer between the carbon shell and the encapsulated metal oxide.

Jin et al. reported a high‐performance bifunctional oxygen electrocatalyst synthesized via a postsynthetic confinement strategy, in which spinel‐type Cu_X_Co_3‐X_O_4_ nanocrystals were encapsulated within multiwalled carbon nanotubes (MWCNTs).^[^ ^86^ ^]^ The synthesis of the Cu_X_Co_3‐X_O_4_@C composite utilized a hydrothermal strategy. Copper and cobalt salts were dissolved in water, and acid‐treated MWCNTs were added. Hydrothermal treatment at 120 °C induced urea decomposition, generating hydroxide ions that coordinated with Cu^2+^ and Co^2+^ to form Cu_3_[Co(OH)_6_]_2_ precursors within the nanotube channels. These were then oxidized at 300 °C for 3 h to yield crystalline Cu_X_Co_3‐x_O_4_ (Figure 2d). High‐resolution transmission electron microscopy (HRTEM) confirmed the successful encapsulation of Cu_X_Co_3‐X_O_4_ nanocrystals inside the MWCNTs, with intimate interfacial contact between the spinel particles and the carbon walls (Figure 2e). Electrochemical evaluation in alkaline electrolyte revealed that the Cu_X_Co_3‐X_O_4_@C catalyst exhibited a significantly more negative polarization curve and higher current density than reference catalysts (Figure 2f). This postconfinement strategy not only enhanced electron transfer efficiency and catalytic performance but also improved structural stability through the protective carbon matrix. The superior activity‐attributed to abundant oxygen vacancies and optimized interfacial charge transfer‐positions Cu_X_Co_3‐X_O_4_@C as a promising candidate for practical energy conversion devices.

Similarly, a novel electrocatalyst, MAPbBr@AlPO‐5 was reported to synthesize via a postconfinement strategy that exploits the ordered porous architecture of AlPO‐5 zeolite.^[^ ^87^ ^]^ As illustrated in Figure 2g, the synthesis involved impregnating AlPO‐5 with a MAPbX_3_ precursor solution (where X denotes halogen), followed by thermal annealing to confine MAPbBr_3_ nanocrystals (NCs) within the zeolite channels. Transmission electron microscopy (Figure 2h) confirmed the uniform dispersion of MAPbBr_3_ NCs (average size ≈ 3.7 nm) within the AlPO‐5 matrix, with a narrow size distribution‐key factor for enhanced electrocatalytic activity. Electrochemical testing in alkaline media revealed exceptional OER performance for MAPbBr_3_@AlPO‐5. Figure 2i showed that the critical role of composition optimization in maximizing OER activity, with MAPbBr@AlPO‐5 emerging as the most efficient catalyst. The study underscores the effectiveness of postconfinement strategies in tailoring nanostructured electrocatalysts for advanced energy applications.

The modulation of reaction pathways and intermediate stabilization by confinement effects is central to understanding the enhanced catalytic performance in nanoconfined systems. This mechanism fundamentally relies on spatial constraints to optimize the microenvironment of active sites, as exemplified by the Cu_X_Co_3‐X_O_4_@C composite: the intimate contact between spinel particles and carbon walls facilitates efficient electron transfer, while the confined environment of carbon nanotubes stabilizes active sites and promotes the formation of intermediates with favorable adsorption energies, thereby enhancing OER efficiency.^[^ ^86^ ^]^ In the MAPbBr_3_@AlPO‐5 composite, the ordered porous architecture of the zeolite framework stabilizes halide perovskite nanocrystals and optimizes their electronic structure, leading to significantly enhanced OER activity.^[^ ^87^ ^]^ Confinement effects essentially regulate reaction pathways by providing a stable environment that lowers the activation energy barrier for specific intermediates, thus collectively improving the catalytic activity and stability of materials. From a general perspective, precise regulation of the electronic structure and transport properties of materials represents a core strategy to enhance electrocatalytic performance.^[^ ^88^ ^]^ Spatial confinement structures, including zeolite pores, carbon nanotubes, and other frameworks, commonly provide a stable antiaggregation environment for active components, optimize electron and mass transport, and mitigate active site loss. Notably, the postconfinement strategy enables researchers to further adjust catalytic performance after material synthesis, offering flexible and customizable optimization pathways for catalyst design.^[^ ^89^ ^]^

### 3.2 In‐situ Confinement Strategy

The in‐situ confinement strategy enables the synthesis of target materials concurrently with the formation of confined spaces. This approach typically involves coassembly of guest and host materials, or direct introduction of guest precursors during host material synthesis to facilitate their growth within confined spaces. The in‐situ strategy offers advantages in precisely controlling the growth location and morphology of nanomaterials, thereby avoiding aggregation of precursors outside the host framework. Furthermore, as the growth of guest materials synchronizes with host formation, it enables better synergistic effects between the two components.^[^ ^90^, ^91^ ^]^

**Figure 3:**
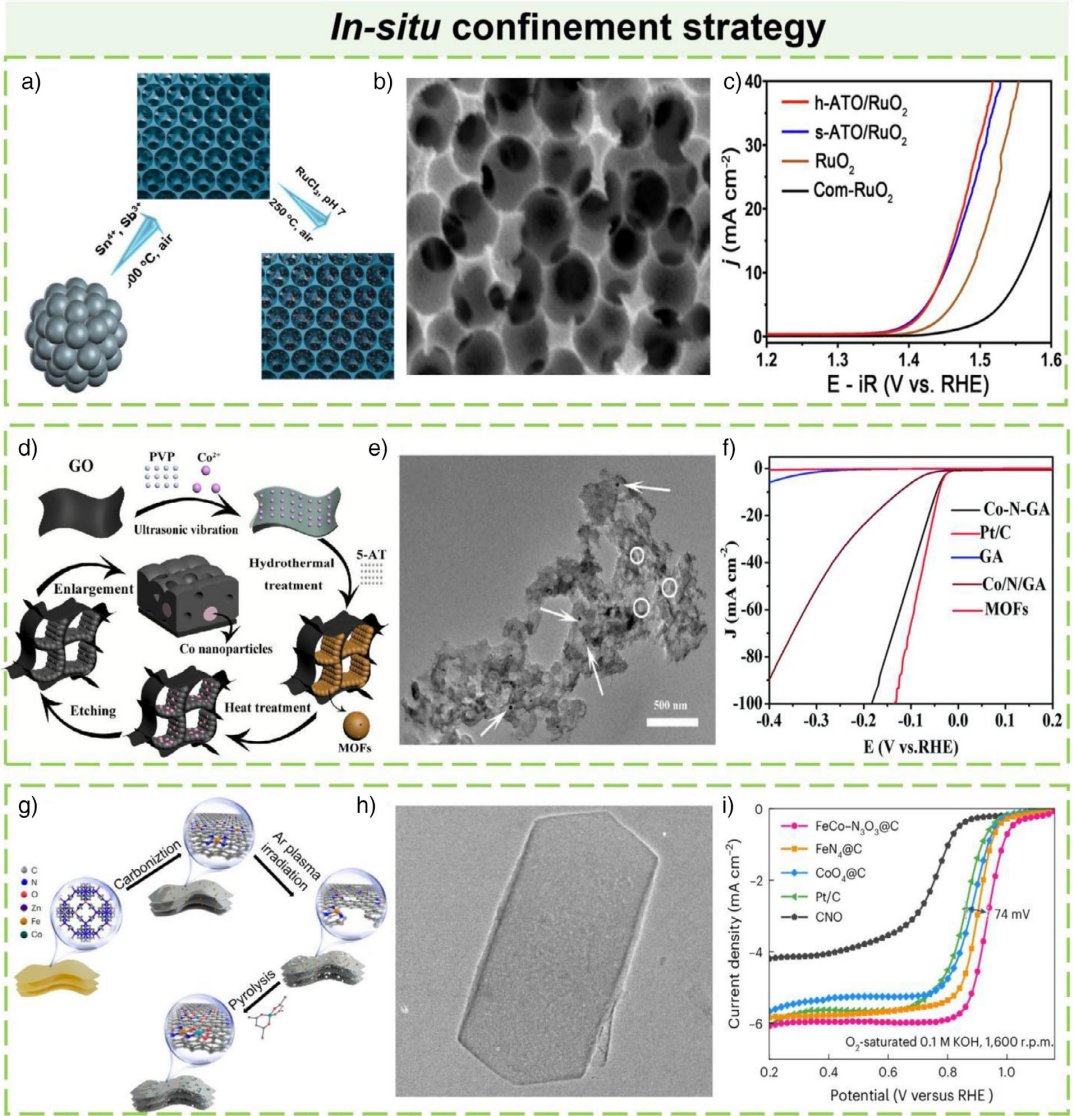
a) Schematic illustration of the h‐ATO/RuO_2_ synthesis process. b) SEM images of h‐ATO/RuO_2_. c) LSV curves of different catalysts with 100% iR correction (*R*: 6 ± 0.3 Ω) were measured in O_2_‐saturated 0.5 M H_2_SO_4_ at room temperature, with a scan rate of 5 mV s^−1^ and RuO_2_ loading of 0.18 mg cm^−2^. Reproduced from Ref.[92] Copyright 2025, with permission from Springer Nature. d) Illustration of the preparation procedure of Co–N–GA. e) TEM of Co–N–GA. f) Polarization curves of Co–N–GA, Co/N/GA, MOFs, GA, and Pt/C. Reproduced from Ref.[93] Copyright 2016, with permission from Royal Society of Chemistry. g) Schematic illustration of the synthesis of FeCo‐N_3_O_3_@C. h) TEM image of the FeN_4_@C precursor. i) LSV curves of FeCo‐N_3_O_3_@C, FeN_4_@C, CoO_4_@C, Pt/C, and CNO in O_2_‐saturated 0.1 M KOH solution at 1600 r.p.m. Reproduced from Ref.[94] Copyright 2024, with permission from Springer Nature.

In 2024, Zheng et al. reported a novel synthesis strategy for an h‐ATO/RuO_2_ catalyst to enhance the OER in proton exchange membrane water electrolysis (PEMWE).^[^ ^92^ ^]^ The synthesis of the RuO_2_ catalyst utilized a templating and confinement strategy. PS spheres were impregnated with tin and antimony precursors, then removed by heating to form the ATO support. RuO_2_ nanoparticles were confined within this support via vacuum degassing, hydrothermal treatment, and annealing. Schematic illustrations and scanning electron SEM images depicted in Figure 3a,b elucidate the synthesis process and the resultant microstructure of the h‐ATO/RuO_2_ catalyst, highlighting the uniform distribution of RuO_2_ nanoparticles on the ATO support. Figure 3c presents a comparison of the OER performance of h‐ATO/RuO_2_ with other catalysts in acidic media. The results indicate that h‐ATO/RuO_2_ exhibits a lower overpotential and higher current density, thereby demonstrating superior electrocatalytic activity. This synthesis approach effectively leverages a confinement effect by spatially confining RuO_2_ nanoparticles within the pores of the honeycomb‐like ATO support.

Zhu et al. reported a method for synthesizing a cobalt‐embedded nitrogen‐doped graphene aerogel (Co–N–GA) catalyst. The synthesis of Co–N–GA employed a hydrothermal and pyrolysis strategy that enabled the uniform embedding of cobalt nanoparticles within a nitrogen‐doped carbon matrix by directly growing MOFs within the pores of graphene oxide (GO).^[^ ^93^ ^]^ This method ensured the homogeneous distribution of active sites and facilitated reactant and product diffusion through its hierarchical porous structure, thereby significantly enhancing the catalyst's performance in the HER. Figure 3d shows the incorporation of cobalt ions and 5‐AT into GO via ultrasonic treatment and hydrothermal assembly to form MOFs. The TEM image of Figure 3e reveals the MOFs' distribution and cobalt nanoparticles within the graphene aerogel. Figure 3f compares the electrocatalytic activity of Co–N–GA with other catalysts in acidic media, highlighting its superior HER performance. The synthesis strategy employed in the preparation of the Co–N–GA catalyst exemplifies the application of principles akin to the in‐situ confinement strategy. By directly growing MOFs within the pores of GO, the method ensures the uniform embedding of cobalt nanoparticles within a nitrogen‐doped carbon matrix. This approach effectively leverages the concept of spatial confinement to enhance the distribution of active sites and optimize the hierarchical porous structure, thereby facilitating the diffusion of reactants and products.

In 2024, Tang et al. reported the development of a Janus dual‐metal site catalyst, FeCo‐N_3_O_3_@C, via a multistep synthetic strategy.^[^ ^94^ ^]^ The synthesis strategy began with the preparation of Fe–N single‐atom catalyst (FeN_4_@C) using 2D MOF nanosheets as precursors, which were pyrolyzed at high temperature to achieve single‐atom dispersion (Figure 3g). Argon plasma irradiation was subsequently employed to introduce vacancies into FeN_4_@C, yielding defect‐rich d‐FeN_3_@C. Finally, Co–O groups were anchored onto the defect sites of d‐FeN_3_@C via low‐temperature pyrolysis of Co(acac)_2_ at 330 °C, a process that ensured precise spatial placement of Co–O moieties as validated by HAADF‐STEM imaging (Figure 3h). Figure 3i demonstrates the OER electrocatalytic performance of FeCo‐N_3_O_3_@C in alkaline media. This approach enables deliberate engineering of active sites to enhance OER performance. Conceptually analogous to in‐situ confinement strategies, this synthetic methodology facilitates precise active site engineering by leveraging tailored environmental conditions for atomic/group anchoring, thus enabling the construction of highly efficient catalysts for oxygen‐related electrocatalytic reactions.

In summary, the in‐situ confinement strategy has proven highly effective in enhancing the performance of catalysts by precisely controlling the spatial distribution and electronic properties of active sites. This approach, as demonstrated in the synthesis of h‐ATO/RuO_2_, Co‐N‐GA, and FeCo‐N_3_O_3_@C, ensures uniform embedding of active components within a tailored host structure, thereby optimizing mass transfer, electron transport, and catalytic activity.^[^ ^92^, ^94^ ^]^ The strategy's ability to prevent precursor aggregation and synchronize guest–host formation not only improves stability and durability but also maximizes synergistic effects, making it a powerful tool for designing advanced catalysts with superior performance in electrochemical reactions.^[^ ^90^ ^]^

In the context of nanoconfinement strategies, both postconfinement and in‐situ confinement offer unique advantages and challenges. Postconfinement strategies involve introducing precursors into preformed confined spaces and then transforming them into the desired materials. This method allows for precise control over the synthesis process, enabling the optimization of material properties such as size, morphology, and electronic structure.^[^ ^91^ ^]^ For instance, the encapsulation of metal oxides within protective matrices not only enhances catalytic activity but also improves stability. In contrast, in‐situ confinement strategies involve the simultaneous formation of the host and guest materials, which can lead to more uniform distribution and better integration of the active components. However, this approach may be more challenging to control and scale up. A systematic comparison reveals that postconfinement strategies provide greater flexibility in adjusting reaction conditions and optimizing material properties after synthesis, while in‐situ confinement strategies offer better control over the nucleation and growth of materials within the confined space. Both approaches have their merits, and the choice between them depends on the specific requirements of the application and the desired material properties.^[^ ^13^ ^]^

## 4 Electrocatalysts for HER in Confined Space

The following section will provide an overview of recent developments in HER catalysts, categorized into three dimensionalities‐1D, 2D, and 3D. These catalysts and their key properties will be systematically discussed and summarized in Table 1, highlighting their advantages, challenges, and potential for industrial‐scale application.

**Table 1:** Properties of 1D–3D confined electrocatalysts for HER.

Electrocatalysts	Dimension	Overpotential at 10 mA cm^−2^ (mV vs RHE)	Tafel slope (mV dec^−1^)	Electrolyte	Stability (h)	Ref.
Co/CNFs	1D	190	66	1 M aq. KOH	200	[99]
WN‐Ni@N, P‐CNT‐800	1D	268	59.8	1 M aq. KOH	30	[97]
Ru_0.33_Se@TNA	1D	57	50	1 M aq. KOH	10	[100]
HUST‐200	1D	131	51	1 M aq. KOH	10	[101]
Co_4_Ni_1_P	1D	129	52	1 M aq. KOH	50	[102]
BP/Co_2_P	2D	336	72	1 M aq. KOH	24	[103]
MXene@RuCo NPs	2D	20	30.4	1 M aq. KOH	−	[104]
Mo_2_TiC_2_T_X_‐PtSA	2D	30	30	0.5 M aq. H_2_SO_4_	100	[19]
Ru_SA_‐N‐S‐Ti_3_C_2_T_X_	2D	76	90	0.5 M aq. H_2_SO_4_	16	[105]
MoS_2_/G	2D	180	79	0.5 M aq. H_2_SO_4_	30	[19]
NiFe@MoS_2_	2D	67	26.8	0.5 M aq. H_2_SO_4_	100	[111]
Ru@TiO_2_	2D	57	67	0.1 M aq. KOH	12	[114]
Ni‐Sn@C	3D	−170	35	0.5 M aq. H_2_SO_4_	−	[120]
Ni_2_P‐UNMs/NF	3D	75	60	1 M aq. KOH	−	[121]
NiCo DASs/N‐C	3D	189	72.5	1 M aq. KOH	−	[125]
Ru_NP_‐Ru_SA_@CFN‐800	3D	33	37	1 M aq. KOH	1400	[23]
Co_3_O_4_‐Mo_2_N	3D	100	162.4	1 M aq. KOH	20	[122]
MI‐PtZnCo	3D	29	64.2	1 M aq. KOH	−	[123]
Pt_3_Fe/NMCS‐A	3D	29	50	1 M aq. KOH	10	[131]

### 4.1 Electrocatalysis in 1D Channels

The 1D nanoconfined space refers to a nanoscale space where one dimension is used to achieve nanoconfinement, with nanotubes being one of the most typical examples of such a structure. 1D channels can be synthesized on a large scale with tunable diameters, making them highly suitable for space‐confined catalytic applications. An optimal diameter enhances activity‐larger ones weaken confinement, while smaller ones lower binding energy, hindering catalysis according to the Sabatier principle.^[^ ^95^, ^96^ ^]^ Length also matters, as overly long 1D channels impede mass transport. Their curved graphene‐like structure distorts π orbitals, shifting electron density outward. This leads to lower reactivity on the inner surface. For encapsulated catalysts, nanoconfinement alters electronic states and redox behavior, significantly influencing catalytic performance.

**Figure 4:**
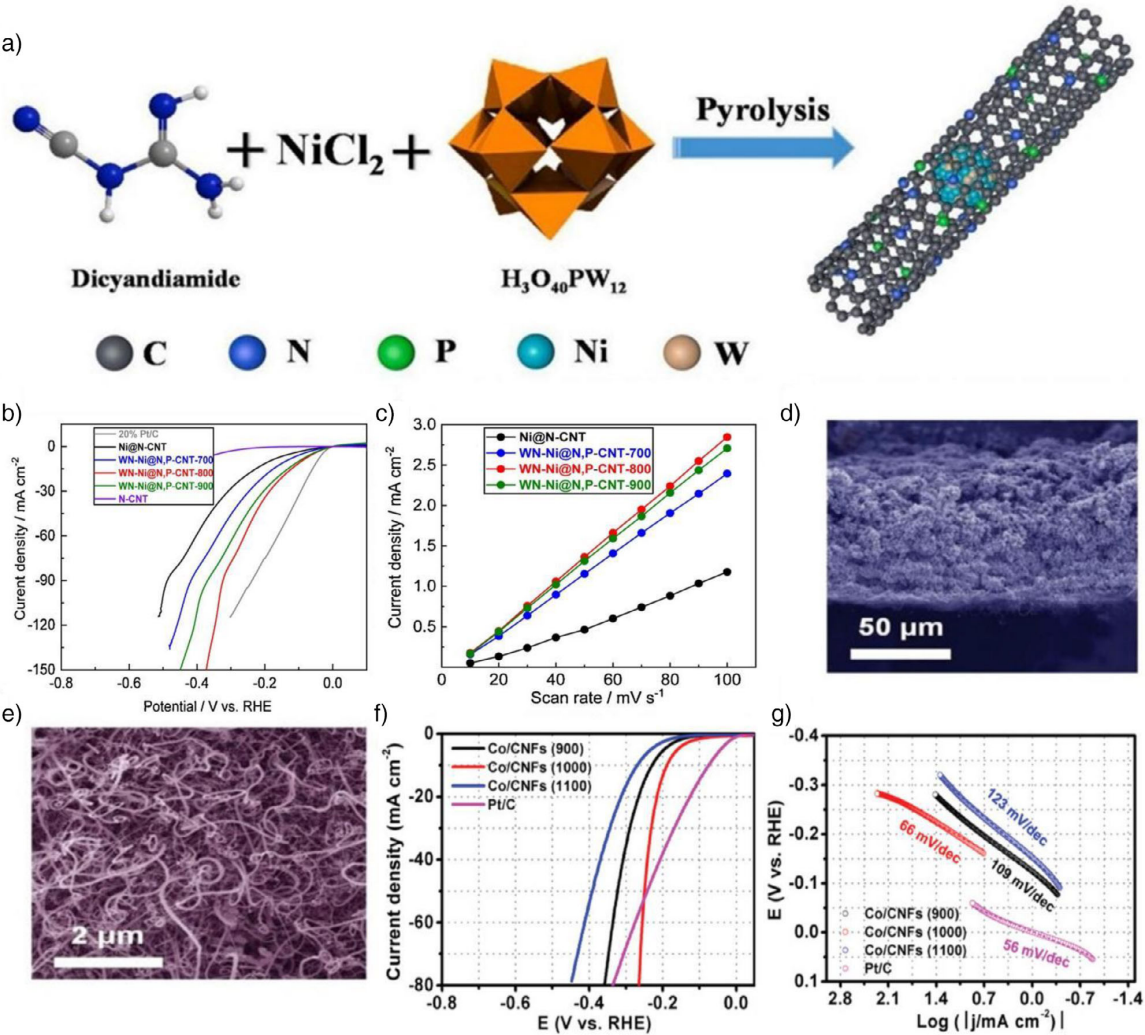
a) Schematic illustration of the synthesis process. b) HER performances. c) The electrochemically active surface areas (ECSAs). Reproduced from Ref.[97] Copyright 2021, with permission from Elsevier. d) Cross‐sectional SEM images and e) top‐view SEM images of Co/CNFs (1000). f) HER linear sweep voltammograms (LSV) plots, g) HER Tafel plots. Reproduced from Ref.[99] Copyright 2019, John Wiley and Sons.

These theoretical understandings have inspired the rational design of CNT‐based catalysts, as exemplified by the following work. For instance, Zhang et al. integrated WN‐Ni Mott–Schottky hetero‐nanoparticles into N,P‐codoped CNTs via one‐pot pyrolysis to synthesize the electrocatalyst WN‐Ni@N,P‐CNT.^[^ ^97^ ^]^ The process entailed heating urea, nickel salt, and phosphotungstic acid to form N,Pco‐doped CNTs encapsulating WN‐Ni heterostructured nanoparticles (Figure 4a). This catalyst combines 1D CNTs confining 0D WN‐Ni heteronanoparticles (1D system). In 1 M KOH, WN‐Ni@N,P‐CNT outperformed Ni@N‐CNT for HER: 70 mV at 10 mA cm^−2^. It also showed the highest Cdl of 29.8 mF cm^−2^ (Figure 4b), indicating more active sites. The specific activity of WN‐Ni@N, P‐CNT‐800 reached 0.61 s^−1^ at −73 mV versus RHE, which is significantly higher than that of Ni@N‐CNT (Figure 4c), underscoring its superior catalytic performance. DFT simulations revealed that the confined WN‐Ni heterostructure not only optimizes the electronic configurations of the Ni and WN phases but also facilitates more efficient charge transfer. The strong electronic coupling between Ni and WN lowers the energy barriers for HER, making the hydrogen adsorption/desorption cycles more favorable. This theoretical insight is consistent with experimental findings, where the WN‐Ni@N,P‐CNT exhibited a significantly higher catalytic performance compared to Ni@N‐CNT. Furthermore, the DFT‐derived insights into the Mott–Schottky heterostructure suggested that the interaction between Ni and WN facilitates efficient water dissociation and hydrogenation steps, which is crucial for optimizing HER performance. WN‐Ni@N, P‐CNT‐800 had a lower Tafel slope (151.7 mV dec^−1^), indicating improved HER intrinsic activity. It retained performance with minimal overpotential increase after 5000 cycles, sustaining 5 mA cm^−2^ for 30 and 330 h of continuous operation. The unique WN‐Ni heterostructure enabled efficient water dissociation and charge transfer via nanoconfinement: confined nanoparticles in N,P‐CNT frameworks enhanced electronic pathways, while heterostructure interfacial engineering increased active‐site electron density, optimizing oxygen adsorption/hydrogenation.

Building on the potential of 1D nanoconfined spaces, certain materials, despite showing low intrinsic catalytic activity in HER, can benefit from their distinctive structural characteristics, providing a unique environment that enhances catalytic performance.^[^ ^98^ ^]^ For example, when electrocatalysts are encapsulated within nanotube structures, the 1D nanochannels can act as confined spaces that boost HER efficiency. CNTs, while not intrinsically catalytic, are widely used as support materials in electrocatalytic systems due to their outstanding chemical stability. Yang et al. successfully synthesized cobalt nanoparticles encapsulated within CNTs using an in‐situ solid‐state diffusion method, where cobalt atoms were extracted from bulk cobalt foil and diffused into nitrogen‐rich carbon. This process creates a unique environment that prevents nanoparticle aggregation, optimizes active site density, and ultimately enhances the catalytic performance for HER efficiency (Figure 4d,e).^[^ ^99^ ^]^ When directly employed as electrodes in HER catalysis, they achieved an overpotential of 0.19 V versus RHE at a current density of 10 mA cm^−2^ and exhibited a Tafel slope of 66 mV dec^−1^ in a 1 M aq. KOH solution (Figure 4f,g). Furthermore, the DFT findings are consistent with experimental observations, where the Co/CNT catalyst maintains stable performance even after 1000 cycles, as confirmed by chronoamperometry and cyclic voltammetry tests. CNTs’ excellent electrical conductivity makes them effective supports in catalysis, offering a large surface area, efficient mass transport, and enhanced electron transfer in HER electrodes, thereby improving performance. Their confined space prevents nanoparticle aggregation, sustains a high active site density, and optimizes catalysis for enhanced HER efficiency. Moreover, DFT simulations provide insights into the underlying mechanisms of nanoparticle confinement, revealing that the interaction between CNTs and nanoparticles creates a unique electronic environment. This environment significantly lowers the energy barrier for hydrogen evolution, making the hydrogen adsorption–desorption cycle more favorable and further improving overall HER efficiency. The stability of these electrodes was confirmed through chronoamperometry and cyclic voltammetry (CV) tests over 1000 cycles.^[^ ^96^ ^]^ CNTs’ excellent electrical conductivity makes them effective supports in catalysis. In HER electrodes, CNTs offer large surface area, efficient mass transport, and boosted electron transfer, improving performance. Additionally, their confined space prevents nanoparticle aggregation, sustains high active site density, and optimizes catalysis for enhanced HER efficiency.^[^ ^96^ ^]^

**Figure 5:**
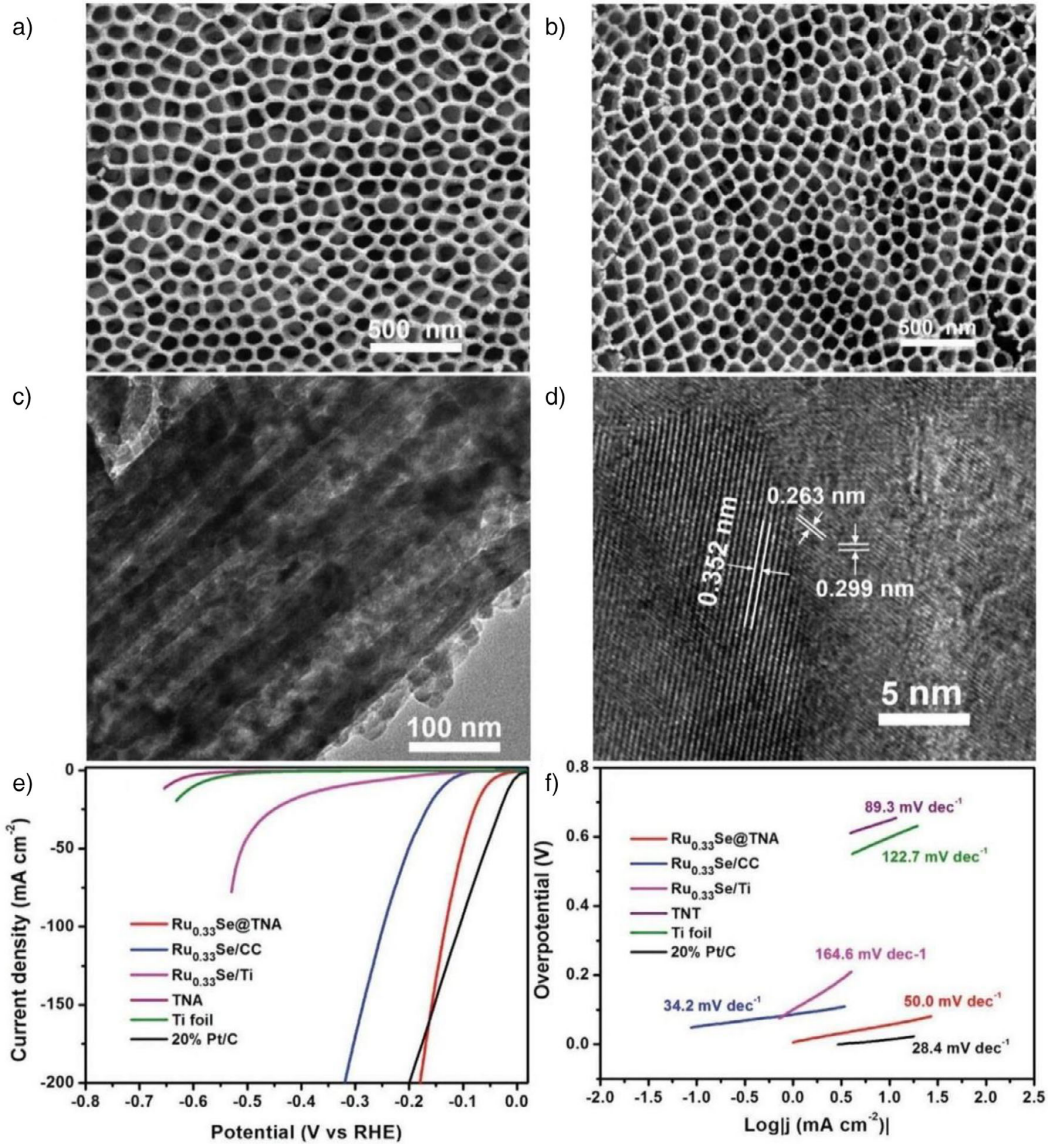
a) SEM images. b) TiO_2_ nanotube arrays. c) Ru_0.33_Se@TNA hybrid, and d) TEM and HRTEM images of the Ru_0.33_Se@TNA hybrid. e) Polarization curves for HER measured on various catalysts, including Ti foil, TiO_2_ nanotube arrays (TNA), Ru_0.33_Se/Ti, Ru_0.33_Se/CC, and the Ru_0.33_Se@TNA hybrid catalyst. f) The corresponding Tafel plots generated from the polarization curves. Reproduced from Ref. [100] Copyright 2018, with permission from John Wiley and Sons.

In contrast, while CNTs offer excellent mechanical properties and are effective supports, other 1D nanomaterials such as TiO_2_, though poorly conductive, offer unique advantages due to their adjustable channel diameters and the prevention of gas accumulation.^[^ ^100^ ^]^ For example, TiO_2_ nanotube arrays decorated with Ru_0.33_Se nanoparticles significantly influence the electronic structure of the catalyst, promoting optimized electron density at the surface and enhancing charge transfer efficiency. The confined geometry of the TiO_2_ nanotube arrays, as revealed by DFT simulations, further improves the electronic properties of the Ru_0.33_Se nanoparticles, reducing the energy barrier for proton adsorption and enhancing the HER kinetics. This effect not only facilitates improved charge transfer but also optimizes the catalytic environment, ultimately boosting the performance of the HER. As revealed by SEM and TEM analyses (Figure 5a–d), the synthesized Ru_0.33_Se@TNA retains the well‐ordered structure of the TiO_2_ nanotube arrays, which exhibit uniform dimensions. Following a hydrothermal reaction, these arrays preserve their morphology, with Ru_0.33_Se nanoparticles uniformly dispersed across the TiO_2_ nanotubes with no noticeable aggregation. This uniform dispersion prevents clogging of the nanotube openings, ensuring a high density of active sites that greatly improve the catalytic efficiency of the HER. Compared to Ru_0.33_Se nanoparticles deposited on Ti foil or carbon cloth, Ru_0.33_Se@TNAs exhibit a significantly lower overpotential of 57 mV at a current density of 10 mA cm^−2^ and a smaller Tafel slope of 50.0 mV dec^−1^ in 1 M aq. KOH, as shown in Figure 5e,f. These enhanced HER properties are attributed to the improved electron transport efficiency, facilitated by the TiO_2_ nanotube structure and the optimized dispersion of Ru_0.33_Se nanoparticles.

In recent years, metal‐organic materials have attracted significant attention as emerging materials. By selecting specific types and employing appropriate preparation methods, nanotube structures can also be constructed from these materials. For instance, copper–ligand mixed metal‐organic nanotubes (MONTs) can act as scaffolds for encapsulating polyoxometalates (POMs), leading to the formation of advanced nanostructures.^[^ ^88^ ^]^ Zhang et al. applied this approach to synthesize two novel polyoxometalate‐encapsulated metal‐organic nanotube frameworks, HUST‐200 and HUST‐201, using hydrothermal assembly with polyoxometalates, copper ions, bipyridine, and tetrazole ligands.^[^ ^101^ ^]^ The HUST‐200 1D nanotube catalyst shows outstanding performance in the HER, with an overpotential of 131 mV at a current density of 10 mA cm^−2^, a Tafel slope of 51 mV dec^−1^, and remarkable stability over 5000 cycles. The performance results from the nanoscale confinement effect of POMs, which improves electron transfer efficiency and enhances catalytic activity, outperforming previous POM‐MOF‐based catalysts. POM encapsulation within the MONT framework changes the electronic structure of copper sites, allowing for more favorable adsorption of reaction intermediates during HER. DFT calculations demonstrate that electron density redistribution around the copper centers lowers the activation energy for proton reduction, further boosting catalytic activity. The confined environment within the nanotubes stabilizes key intermediates, preventing desorption and improving electron transfer efficiency. This nanoscale confinement effect has been confirmed by DFT‐derived reaction pathways, showing that the interaction between POM and the MONT framework enhances stability and reactivity under harsh reaction conditions.^[^ ^88^ ^]^

Inactive nanotubes can serve as templates for HER‐active catalytic nanotubes. MOFs, with tunable channels and metal centers, are effective templates. Heat treatment, sulfuration, or phosphorization can convert MOFs into metal oxides, sulfides, phosphides, or metal‐loaded carbon composites, preserving their morphology and channel structure.^[^ ^96^ ^]^ For example, Yan et al. synthesized two novel nickel‐cobalt bimetallic phosphide nanotube catalysts using a low‐temperature phosphorization process from a bimetallic metal‐organic framework (MOF‐74) precursor.^[^ ^102^ ^]^ These catalysts are 1D Ni‐Co bimetallic phosphide nanotubes fabricated by incorporating Ni/Co into MOF‐74 frameworks followed by phosphorization. In an alkaline solution (1.0 M aq. KOH), the catalysts exhibited exceptional HER performance, with an overpotential of 129 mV at a current density of 10 mA cm^−2^ and a Tafel slope of 52 mV dec^−1^. Additionally, the catalysts demonstrated excellent electrochemical stability after 5000 cycles. This HER performance results from the nanoconfinement of Ni–Co phosphide nanotubes, which boosts electron transfer and active site utilization for improved catalysis. The hollow structure offers large surface area, facilitating reactant access to active sites and further enhancing efficiency.

Confined space catalysts, such as 1D nanostructures like CNTs or MOFs, enhance HER by restricting reactant movement, increasing local concentrations, and improving mass transfer. These features optimize reactant–catalyst interactions, boosting efficiency and selectivity. DFT simulations show confinement impacts electronic structure, adsorption, and reaction pathways, with graphene and MOFs improving hydrogen adsorption and stabilizing intermediates. Although 1D systems offer high surface area and efficient electron transport, their mass transport limitations at higher current densities hinder catalytic efficiency, spurring interest in 2D confined catalysts for better control and superior HER performance.

### 4.2 Electrocatalysis in 2D Layered Spaces

Various 2D materials, including layered double hydroxides (LDHs), black phosphorene (BP), transition metal dichalcogenides, graphene and its derivatives, graphitic carbon nitride (g‐C_3_N_4_), MXenes, carbon networks, and graphdiyne, have emerged as promising candidates for electrocatalysis in HER applications. These materials typically feature weak interlayer interactions, allowing atoms or molecules to react within the confined spaces between their layers or between the material and other substrates. In this 2D nanoconfined space, reactants, intermediates, and products interact with the surface of the material, and their movement is constrained in the direction perpendicular to the plane of the layers. This unique confinement effect enhances HER efficiency by optimizing the interaction between reactants and active sites, making these materials highly effective for improving hydrogen evolution in electrochemical processes.^[^ ^18^ ^]^

2D layered materials offer an additional type of host material capable of confining HER catalysts.^[^ ^98^ ^]^ Most 2D materials, bonded by weak van der Waals forces, feature tunable interlayer spacing (0.1‐several nm) in their van der Waals gaps, making them ideal carriers for HER catalysts. For example, BP exhibits higher catalytic activity on edge planes than basal planes, yet its overall HER performance remains lower than many existing electrocatalysts.^[^ ^103^ ^]^ To enhance its HER performance, BP is often hybridized with more active materials. For example, BP/Co_2_P composites have been synthesized and exhibit excellent HER performance, achieving an onset overpotential of 105 mV and a Tafel slope of 62 mV dec^−1^ in 0.5 M H_2_SO_4_(aq). From a theoretical perspective, DFT calculations offer valuable insights into the atomic‐level interaction mechanisms BP/Co_2_P within their heterostructure. DFT simulations indicate that Co_2_P nanoparticles preferentially anchor to the edge defects of BP nanosheets due to the higher electronic density and increased reactivity at these sites. This selective binding significantly lowers the energy barriers for proton adsorption, a key step in the HER catalysis. Additionally, the confined environment created by the BP matrix plays a crucial role in stabilizing the Co_2_P nanoparticles, preventing their aggregation and ensuring a high density of active sites, thus enhancing catalytic efficiency. The synergistic interaction between BP and Co_2_P not only improves the overall electrocatalytic performance but also modifies the electronic properties of the system, promoting faster charge transfer and reducing the overpotentials required for HER. These theoretical findings align with experimental observations, further highlighting the potential of BP/Co_2_P heterostructures for efficient electrochemical water splitting. Anchoring Co_2_P significantly improves electrical conductivity and increases the density of active sites. This improvement is largely attributed to the confined environment within the BP structure, which restricts the aggregation of Co_2_P and maintains a high density of exposed active sites, thereby enhancing catalytic efficiency.

**Figure 6:**
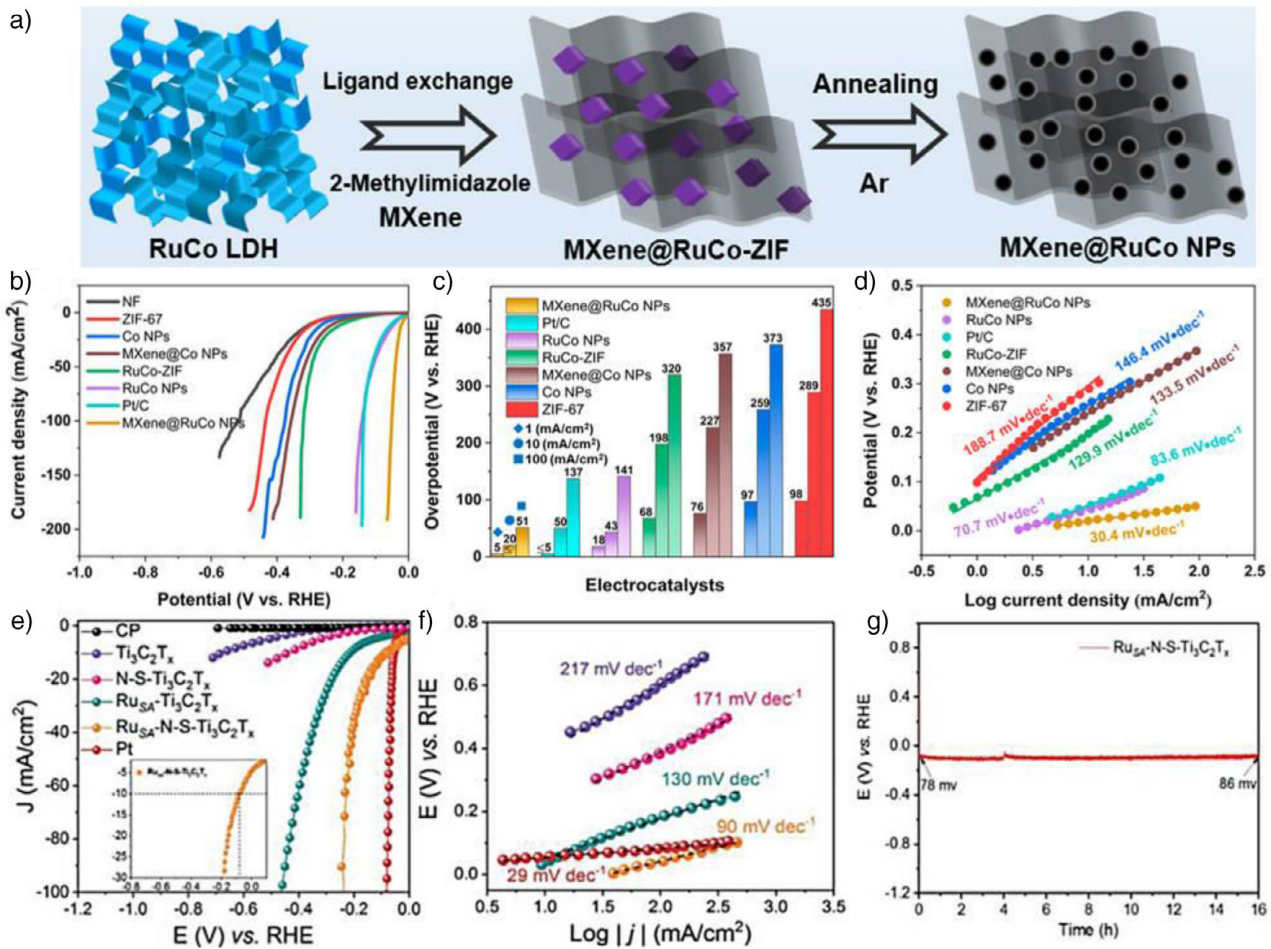
a) Synthesis illustration. b)–c) Electrocatalytic performance for HER. d) the Corresponding Tafel plots for HER analysis. Reproduced from Ref.[104] Copyright 2023, with permission from American Chemical Society. e) Polarization curves for bare carbon paper, Ti_3_C_2_T_X_, N‐S‐Ti_3_C_2_T_X_, Ru_SA_‐Ti_3_C_2_T_X_, Pt, and Ru_SA_‐N‐S‐Ti_3_C_2_T_X_ samples measured in 0.5 M aq. H_2_SO_4_. Inset: enlarged view of the HER polarization curve for Ru_SA_‐N‐S‐Ti_3_C_2_T_X_. f) The corresponding Tafel plots. g) Long‐term stability test of the Ru_SA_‐N‐S‐Ti_3_C_2_T_X_ catalyst at 10 mA cm^−2^ in 0.5 M aq. H_2_SO_4_. Reproduced from Ref.[105] Copyright 2019, with permission from Wiley‐VCH.

Beyond BP, 2D materials like MXene and graphene serve as supports, offering similar benefits for stabilizing and optimizing confined catalysts. MXenes, notable for their large specific surface area, tunable bandgap, excellent conductivity, and robust mechanical stability, are ideal catalyst supports enabling effective stabilization and performance enhancement.^[^ ^103^ ^]^ Li et al. developed an electrocatalyst called MXene@RuCo NPs, derived from a metal‐organic framework and optimized it using a collaborative interface strategy (Figure 6a).^[^ ^104^ ^]^ The LSV analysis for HER revealed that the MXene@RuCo NPs exhibit exceptional performance (Figure 6b), requiring an overpotential of only 20 mV to achieve a current density of 10 mA cm^−2^. This value is significantly lower compared to RuCo NPs (43 mV), RuCo‐ZIF (198 mV), MXene@Co NPs (227 mV), Co NPs (263 mV), ZIF‐67 (289 mV), and even commercial Pt/C (50 mV), highlighting the superior efficiency of MXene@RuCo NPs. Additionally, as depicted in Figure 6c, the catalyst demonstrated an exceptionally low onset overpotential of less than 5 mV at an onset current density of 1 mA cm^−2^, indicating a rapid current response. Furthermore, the Tafel plot analysis in Figure 6d confirms the superior HER kinetics of MXene@RuCo NPs, evidenced by a remarkably low Tafel slope of 30.4 mV dec^−1^. This slope is substantially lower than that of RuCo NPs (70.7 mV dec^−1^), RuCo‐ZIF (129.9 mV dec^−1^), MXene@Co NPs (133.5 mV dec^−1^), Co NPs (146.4 mV dec^−1^), ZIF‐67 (188.7 mV dec^−1^), and Pt/C (83.6 mV dec^−1^), underscoring the faster HER kinetics and overall catalytic efficiency. The Ru doping in the Co lattice optimizes electronic structure and generates active sites. MXene nanosheets, with high surface area and excellent conductivity, facilitate charge transfer and enhance electrolyte interaction with RuCo nanoparticles. Importantly, the confinement of RuCo within 2D MXene prevents aggregation, increasing active site exposure and catalytic efficiency. Ru–Co synergies and MXene's unique confinement significantly boost the catalyst's HER performance and stability.^[^ ^104^ ^]^

Building on the success of MXene‐supported catalysts, further efforts have focused on fine‐tuning the structure and enhancing the stability of these systems through novel configurations. For example, Zhang et al. synthesized the MXene‐supported single‐atom Pt catalyst (Mo_2_TiC_2_T_x_‐PtSA) via electrochemical exfoliation, anchoring single Pt atoms at Mo vacancies in the 2D MXene matrix. This stable 2D composite structure exhibited outstanding HER performance, with an overpotential of only 30 mV at a current density of 10 mA cm^−2^ and a Tafel slope of 30 mV dec^−1^ in a 0.5 M aq. H_2_SO_4_ solution, indicating high catalytic efficiency. Additionally, the catalyst demonstrated remarkable stability, significantly surpassing commercial Pt/C catalysts, which required an overpotential of 65 mV. This superior performance is attributed to the redistribution of the electronic structure caused by the incorporation of single platinum atoms, enhancing the electron environment during the HER process. Moreover, the nanoconfinement effect‐preventing Pt atom aggregation‐further optimizes the catalyst's electrochemical performance.^[^ ^19^ ^]^ Similarly, Ramalingam and coworkers developed a Ru single‐atom catalyst (Ru_SA_‐N‐S‐Ti_3_C_2_T_X_) supported on Ti_3_C_2_T_X_ MXene by coordinating with nitrogen and sulfur species.^[^ ^19^, ^105^ ^]^ This catalyst exhibited a remarkably low overpotential of 76 mV, which is comparable to that of commercial Pt (53 mV), and significantly lower than other configurations like Ti_3_C_2_T_X_ (673 mV) and N‐S‐Ti_3_C_2_T_X_ (453 mV), demonstrating its excellent HER properties (Figure 6e). The Tafel slope for Ru_SA_‐N‐S‐Ti_3_C_2_T_X_ was measured at 90 mV dec^−1^, indicating superior HER kinetics (Figure 6f). Furthermore, the catalyst displayed exceptional durability, maintaining stable performance with minimal changes in electrochemical impedance spectra (EIS) even after extensive CV testing under neutral and alkaline conditions (Figure 6g). The unique confinement provided by the MXene framework significantly enhances the performance and stability of the catalyst during HER.^[^ ^105^ ^]^

The channels in 2D materials, including van der Waals gaps and interlayer spaces, enhance HER performance by encapsulating electrocatalysts. This confinement improves catalyst dispersion, stability, and modifies the adsorption properties of intermediates. These changes optimize interactions between the catalyst and intermediates, boosting HER efficiency. Encapsulating electrocatalysts within specific 2D materials significantly enhances HER performance. For example, DFT calculations investigated hydrogen adsorption on metal substrates (Ni, Pd, Rh, Pt, Cu, Au, and Ag) beneath graphene layers, exploring interfacial interactions critical for catalytic activity.^[^ ^106^ ^]^ Zhou et al. demonstrated that confining hydrogen between the graphene cover and the metal surface modifies hydrogen adsorption energies, thereby affecting HER activity. Their findings indicated that the nanoconfinement created at the graphene–metal interface weakens hydrogen adsorption compared to uncovered metal surfaces, resulting in differences in HER rates.^[^ ^88^, ^106^ ^]^

**Figure 7:**
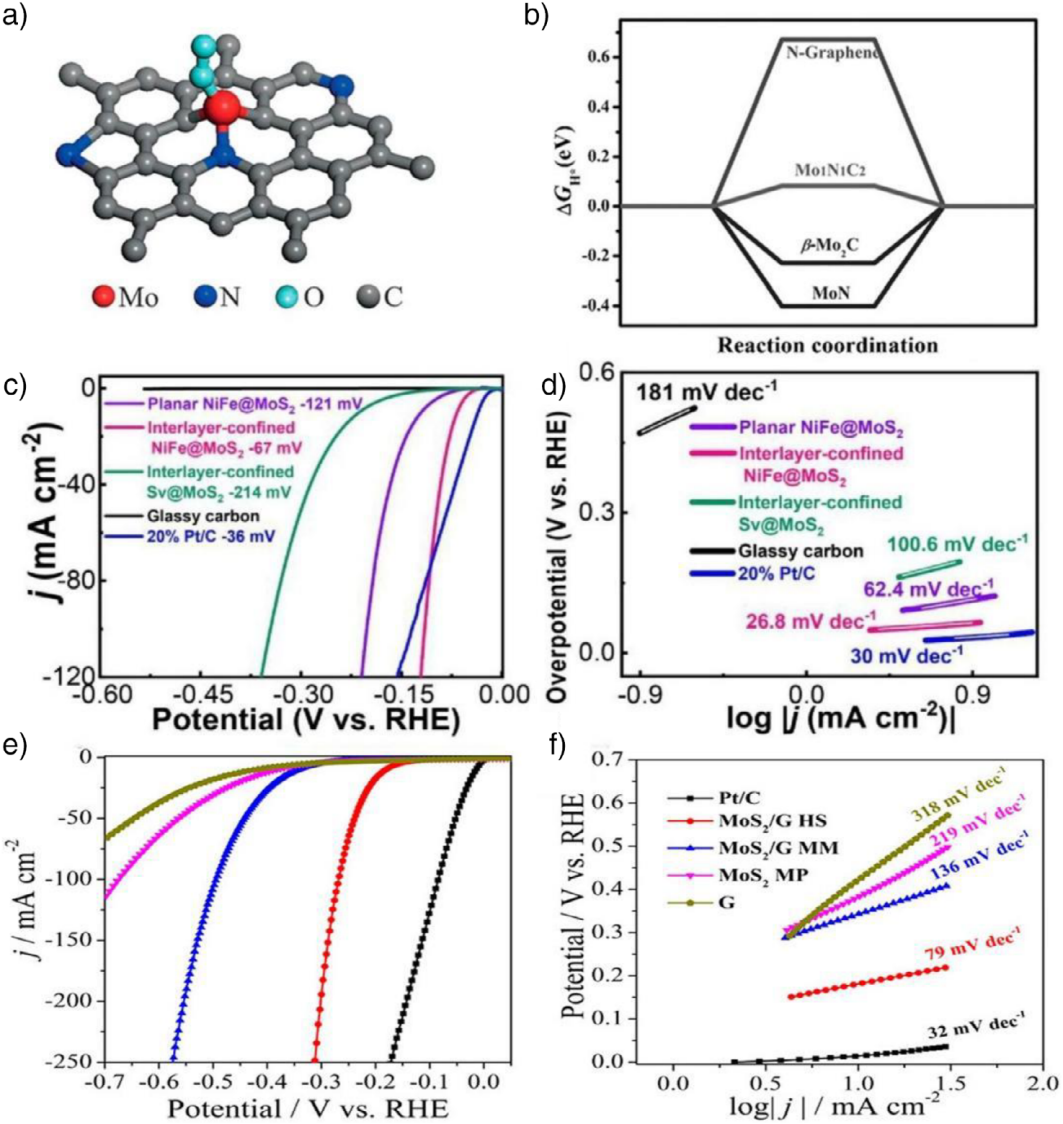
a) Atomic structure model of graphene with a confined Mo_1_N_1_C_2_ center. b) Calculated Δ*G*_H_* for H* adsorption on various Mo‐based catalysts in HER. Reproduced from Ref.[108] Copyright 2017, with permission from John Wiley and Sons. c) Polarization curves. d) Tafel plots for interlayer‐confined NiFe@MoS_2_, planar NiFe@MoS_2_, interlayer‐confined Sv@MoS_2_, bare glassy carbon electrodes, and 20% Pt/C for HER. Reproduced from Ref.[111] Copyright 2023, with permission from John Wiley and Sons. e) LSV curves of MoS_2_/G HS, MoS_2_/G MM, MoS_2_ MP, G, and commercial Pt/C electrodes for HER, recorded at a scan rate of 10 mV s^−1^. f) The corresponding Tafel plots. Reproduced from Ref.[19] Copyright 2020, with permission from American Chemical Society.

Graphene‐confining single‐atom catalysts can also deliver outstanding HER performance. Chen and colleagues developed a system where individual Mo atoms are confined within N‐doped graphene (Figure 7a).^[^ ^107^, ^108^ ^]^ This system exhibited enhanced activity compared to Mo_2_C and MoN bulk catalysts and demonstrated superior stability relative to commercial Pt/C. As depicted in Figure 7b, the calculated Gibbs free energy (∆*G*_H_*) for the Mo_1_N_1_C_2_ site is closest to zero among the four catalysts studied, indicating its high efficiency for H_2_ production. This confined environment within the N‐doped graphene lattice not only prevents the aggregation of Mo atoms but also optimizes the electronic structure around the active sites, enhancing both the stability and the efficiency of the catalyst. Additionally, a single Ni atom confined within the graphene lattice, substituting a carbon atom, led to notable HER activity and excellent cycling stability.^[^ ^107^, ^109^ ^]^ DFT calculations indicated that substitutional dopants occupying carbon sites exhibited higher HER activity than Ni atoms anchored at defect sites, further emphasizing the importance of precise atomic confinement in enhancing electrocatalytic performance.^[^ ^107^ ^]^

Dual‐atom confinement in 2D layered materials represents a promising approach to enhance catalytic activity and stability for HER. Confining dual atoms within the van der Waals gaps of such structures is expected to boost the kinetic and energetic properties of catalytic processes, driving further improvements in performance.^[^ ^110^ ^]^ Jiang et al. synthesized a layer‐confined NiFe dual‐atom catalyst, named NiFe@MoS_2_, using a laser molecular beam epitaxy technique and subsequent self‐curving treatment.^[^ ^111^ ^]^ This catalyst consists of Ni and Fe dual‐atom sites within the 2D MoS_2_ nanosheet, forming a stable 2D composite structure. In a 0.5 M aq. H_2_SO_4_ solution, NiFe@MoS_2_ exhibited excellent HER performance, with an overpotential of only 67 mV at a current density of 10 mA cm^−2^ and a Tafel slope of 26.8 mV dec^−1^, indicating very high catalytic efficiency (Figure 7c,d). To further elucidate the catalytic mechanism and stability of the NiFe@MoS_2_ catalyst under working conditions, X‐ray absorption spectroscopy (XAS) was employed. The XAS results revealed that the oxidation states of Ni and Fe atoms remained stable during the HER process, indicating the robustness of the interlayer confinement effect in maintaining the structural integrity of the dual‐atom sites. Additionally, the XAS spectra showed no significant changes in the local coordination environment of the Ni and Fe atoms, suggesting that the interlayer confinement effectively protected the dual atoms from degradation and aggregation under harsh acidic conditions. Additionally, the catalyst demonstrated significant stability during long‐term tests, outperforming traditional planar NiFe@MoS_2_ structures, as shown by the stable current during amperometric j‐t tests.^[^ ^111^ ^]^ This superior performance is primarily attributed to the interlayer confinement effect, which enhances the adsorption strength at the active sites and provides protection for the dual atoms, preventing aggregation and degradation under harsh acidic conditions. DFT calculations further support this, indicating that the interlayer confinement not only stabilizes the dual atoms but also ensures better catalytic performance by mitigating aggregation and enhancing the overall stability under acidic conditions.

Nonmetal atoms like nitrogen have been explored to improve HER in graphene‐based systems, while recent studies focus on 2D heterostructures that enhance catalytic activity through strong interfacial coupling and structural confinement. Encapsulating 2D catalysts within 2D active host channels is a promising approach for developing innovative HER catalysts.^[^ ^112^ ^]^ The 2D van der Waals heterostructures, characterized by face‐to‐face contact, can establish a strongly coupled interface, which could significantly enhance catalytic performance. For instance, Yu and colleagues constructed a heterostructure composed of alternating layers of ultrasmall monolayer MoS_2_ nanosheets and ultrathin graphene.^[^ ^19^ ^]^ Density functional theory calculations revealed that the interplanar distance within the heterostructure expands to 1.104 nm, compared to 0.615 nm in pristine MoS_2_. During HER testing, the heterostructure exhibits catalytic activity with an overpotential of 180 mV at 10 mA cm^−2^ and a Tafel slope of 79 mV dec^−1^ in 0.5 M aq. H_2_SO_4_ (Figure 7e). These performance metrics are significantly better than those of pristine MoS_2_ (383 mV; 219 mV dec^−1^), graphene (423 mV; 318 mV dec^−1^), and their mechanical mixture (343 mV; 136 mV dec^−1^). The superior HER activity is attributed to the expanded interplanar spacing, which enhances hydrogen adsorption, and the strong interfacial interaction between MoS_2_ and graphene, which facilitates charge transfer (Figure 7f).^[^ ^19^ ^]^ DFT calculations revealed that the interplanar distance within the heterostructure expands to 1.104 nm, compared to 0.615 nm in pristine MoS_2_. This expanded spacing not only increases the density of exposed edge sites but also significantly alters the electronic environment of the MoS_2_ sheets. DFT results demonstrate that such structural modulation leads to a substantial shift in the hydrogen adsorption free energy (Δ*G*_H_^*^), from −0.72 eV in pristine MoS_2_ to −0.28 eV in the MoS_2_/graphene heterostructure. This closer‐to‐zero Δ*G*_H_^*^ indicates a more thermodynamically favorable hydrogen adsorption–desorption balance, which is critical for efficient HER catalysis. Additionally, 2D confinement inhibits MoS_2_ nanosheet aggregation, stabilizes the catalyst, and optimizes the electronic environment, significantly enhancing catalytic activity and durability.

In the pursuit of enhancing the efficiency of electrocatalysts for the HER in alkaline media, Zhou et al. developed a novel catalyst: Pt clusters confined within porous amine cages (Pt/cage). This 2D catalyst utilizes the synergistic interaction between Pt clusters and the amine cage framework, resulting in exceptional HER performance with an overpotential of 32 mV at 10 mA cm^−2^ and a Tafel slope of 37 mV dec^−1^.^[^ ^113^ ^]^ The excellent HER performance can be attributed to the –NH– moiety of the cage framework, which modulates the interfacial water hydrogen‐bond network to facilitate charge transfer, and via the Grotthuss mechanism, lowers the kinetic barrier for hydrogen adsorption. The confined environment enhances proton transfer efficiency and ensures close proximity between Pt clusters and water, improving catalytic synergy. The catalyst also showed excellent stability, with only a 5.1% current density loss after 10,000 cycles, attributed to the robust confined structure that protects the Pt clusters from degradation. This synergistic interaction not only boosts catalytic activity but also improves durability.

**Figure 8:**
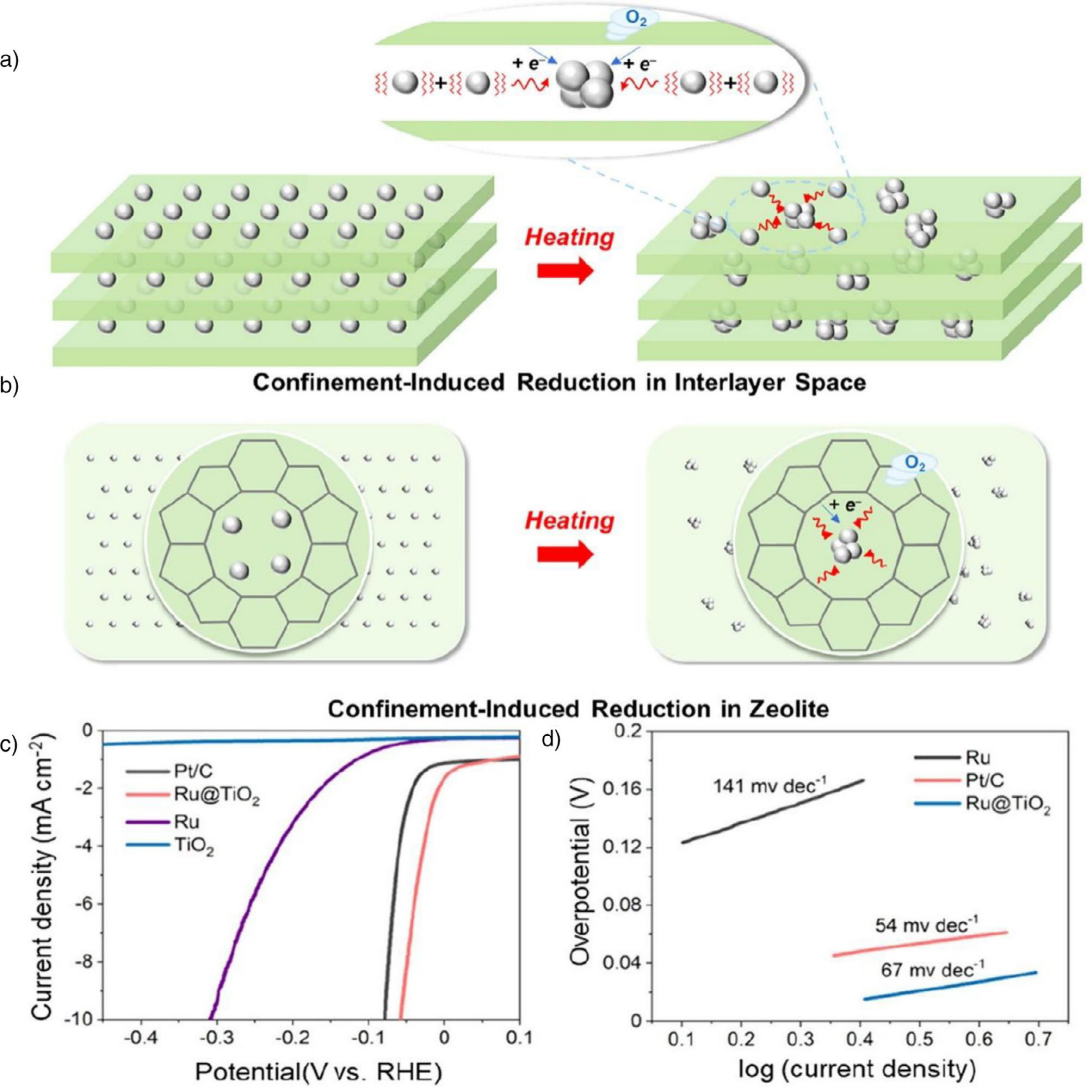
a) and b) Reduction of metal ions induced by confinement within a layered structure (top panel) and a zeolite framework (bottom panel). c) LSV curves for Ru@TiO_2_, commercial Pt/C, Ru, and TiO_2_. d) Tafel slopes for Ru@TiO_2_, commercial Pt/C, and Ru. Reproduced from Ref.[114] Copyright 2024, with permission from American Chemical Society.

As studies continue to explore various methods to improve HER performance through atomic and nanoscale confinement, recent advancements have demonstrated the potential of using confined nanoparticles in 2D structures for enhanced catalytic efficiency. Liang et al. synthesized a size‐tunable Ru nanoparticle catalyst (Ru@TiO_2_) using an ion‐sieve‐confined reduction technique (Figure 8a,b).^[^ ^114^ ^]^ This 2D composite structure features Ru nanoparticles uniformly dispersed on a TiO_2_ matrix. The confined environment provided by the TiO_2_ interlayer spaces facilitated the precise reduction of Ru^3+^ to Ru^0^, preventing aggregation and ensuring uniform nanoparticle distribution. The Ru@TiO_2_ exhibited superior HER performance in a 0.1 M aq. KOH solution, with an overpotential of 57 mV at 10 mA cm^−2^, outperforming Pt/C (87 mV) (Figure 8c). This enhanced activity is attributed to the strong metal–support interaction between Ru and TiO_2_, which optimizes the hydrogen adsorption of Gibbs free energy, accelerating the HER process. The Tafel slope of Ru@TiO_2_ was 67 mV dec^−1^, comparable to that of Pt/C (54 mV dec^−1^), indicating efficient kinetics (Figure 8d). The catalyst also demonstrated excellent stability, maintaining performance over 1000 cycles and 12 h of testing. The Ru@TiO_2_ prepared by the ion‐sieve‐confined reduction had the best performance. The combination of high activity and low cost of Ru@TiO_2_ suggests it will be an efficient substitute for Pt/C in commercial applications. The superior catalytic activity of Ru@TiO_2_ is attributed to the strong metal–support interaction between the Ru nanoparticles and the TiO_2_ matrix, which facilitates electron transfer and optimizes hydrogen adsorption energy.

2D confined space catalysts, such as MXene, graphene, and transition metal dichalcogenides, enhance HER by limiting reactant movement, increasing local concentration, and improving mass transfer. These structural features optimize the interaction between reactants and catalyst surfaces, boosting HER efficiency.^[^ ^18^ ^]^ For example, MXene's interlayer confinement prevents active site aggregation, and graphene's high surface area facilitates charge transfer. DFT studies demonstrate how confinement alters hydrogen adsorption and stabilizes intermediates, improving catalytic performance. However, the relatively narrow confinement in 2D systems can limit active site exposure and reduce efficiency at higher current densities. Additionally, the structural stability and long‐term performance of 2D materials under harsh conditions pose challenges. These limitations suggest that 3D confined catalysts, with larger surface areas and better mass transport properties, may offer significant advantages in enhancing HER efficiency and stability.^[^ ^115^ ^]^

### 4.3 Electrocatalysis in 3D Confined Spaces

3D confined space catalysts have emerged as a promising strategy for enhancing the HER. Unlike 1D and 2D confined spaces, which restrict reactant movement along a single or 2D plane, 3D confined spaces typically provide greater freedom for reactants, intermediates, and products to interact within the material. This allows for efficient mass transport, more accessible active sites, and improved catalytic efficiency. The use of spherical templates and nanoconfined spaces, such as those in MXene, Ni–Sn alloy nanoparticles encapsulated in porous carbon shells, and other 3D structures, significantly boosts the interaction between reactants and the catalyst surface, accelerating HER performance. Moreover, 3D catalysts help prevent nanoparticle aggregation, stabilize active sites, and optimize electron transfer, enhancing both catalytic activity and stability.^[^ ^116^ ^]^

In a similar vein, while the constituent units of certain structures may be 1D or 2D, their arrangement within a 3D framework allows for enhanced catalytic performance.^[^ ^117^ ^]^ These 3D channels may arise from cavities within the encapsulating materials, the porosity of the support materials, or from the stacking of the materials themselves in a 3D order, and so on.^[^ ^118^, ^119^ ^]^ For instance, uniform spherical 3D channels can be formed through a core‐shell structure consisting of Ni–Sn alloy nanoparticle cores surrounded by a porous carbon shell (Ni‐Sn@C).^[^ ^120^ ^]^ The porous carbon shell structure facilitates efficient electrolyte transport and confines the HER within these 3D channels. When used as an HER catalyst, the reaction occurring within the 3D channels demonstrated an onset potential of −170 mV and a Tafel slope of approximately 35 mV dec^−1^ in acidic media.^[^ ^120^ ^]^

**Figure 9:**
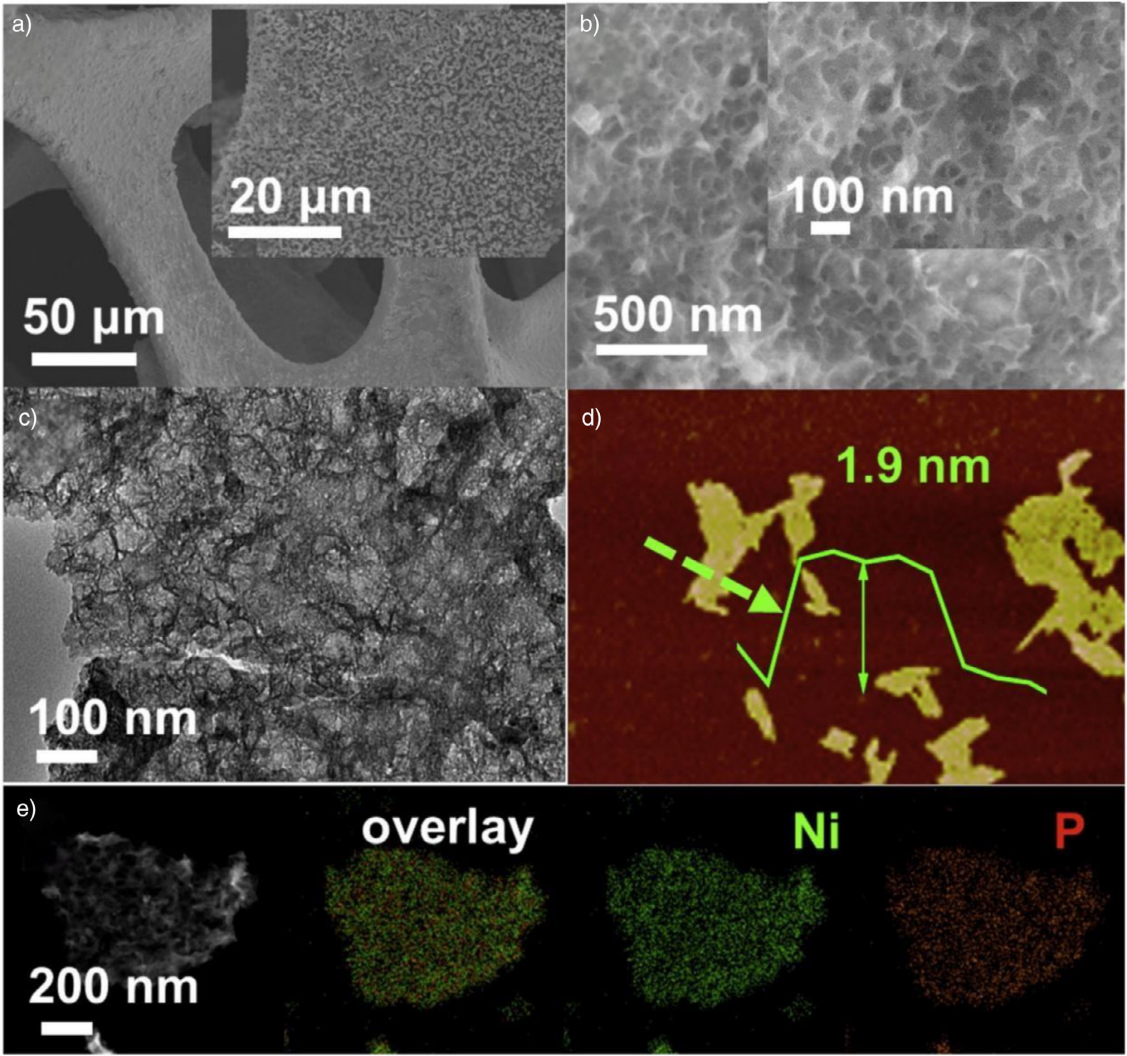
a) SEM images. b) High‐resolution SEM (HR‐SEM) image. c) TEM image. d) AFM image with corresponding height profile, and e) STEM‐EDX elemental mapping of Ni_2_P‐UNMs/NF nanocomposites. Reproduced from Ref.[121] Copyright 2020, with permission from Elsevier.

In a similar fashion, the integration of porous support materials, such as nickel foam (NF), further enhances the performance of 3D catalysts by facilitating effective mass transport and stabilizing the active sites. An example is the Ni_2_P nanomeshes grown on NF (Ni_2_P‐UNMs/NF).^[^ ^121^ ^]^ The NF substrate provides macroporous channels (Figure 9a), which enhance the mass transport of electrolytes, reactants, and products during electrocatalytic reactions. These nanomeshes consist of ultrathin, porous Ni_2_P nanosheets with a thickness of approximately 1.9 nm, as confirmed by AFM analysis (Figure 9b). TEM images further corroborate the sheet‐like morphology of Ni_2_P‐UNMs (Figure 9c,d), while STEM‐EDX mapping reveals a uniform distribution of Ni and P, confirming the effective phosphidation of Ni(OH)_2_ to Ni_2_P (Figure 9e). When employed as a HER electrode, Ni_2_P‐UNMs/NF exhibited an overpotential of just 75 mV at a current density of 10 mA cm^−2^ and a Tafel slope of 60 mV dec^−1^. The confined space within these 3D channels plays a crucial role in maintaining nanoparticle dispersion, optimizing electron transport, and stabilizing the active sites, leading to the enhanced catalytic performance observed in HER. For Ni_2_P‐UNMs/NF, the DFT results further demonstrate that the confinement effects within the porous nanomeshes stabilize the Ni_2_P active sites, preventing aggregation and degradation under harsh electrochemical conditions. These theoretical findings underscore the significance of both spatial confinement and the distinct edge effects in optimizing catalytic performance, resulting in enhanced HER activity with an overpotential of just 75 mV at a current density of 10 mA cm^−2^.

**Figure 10:**
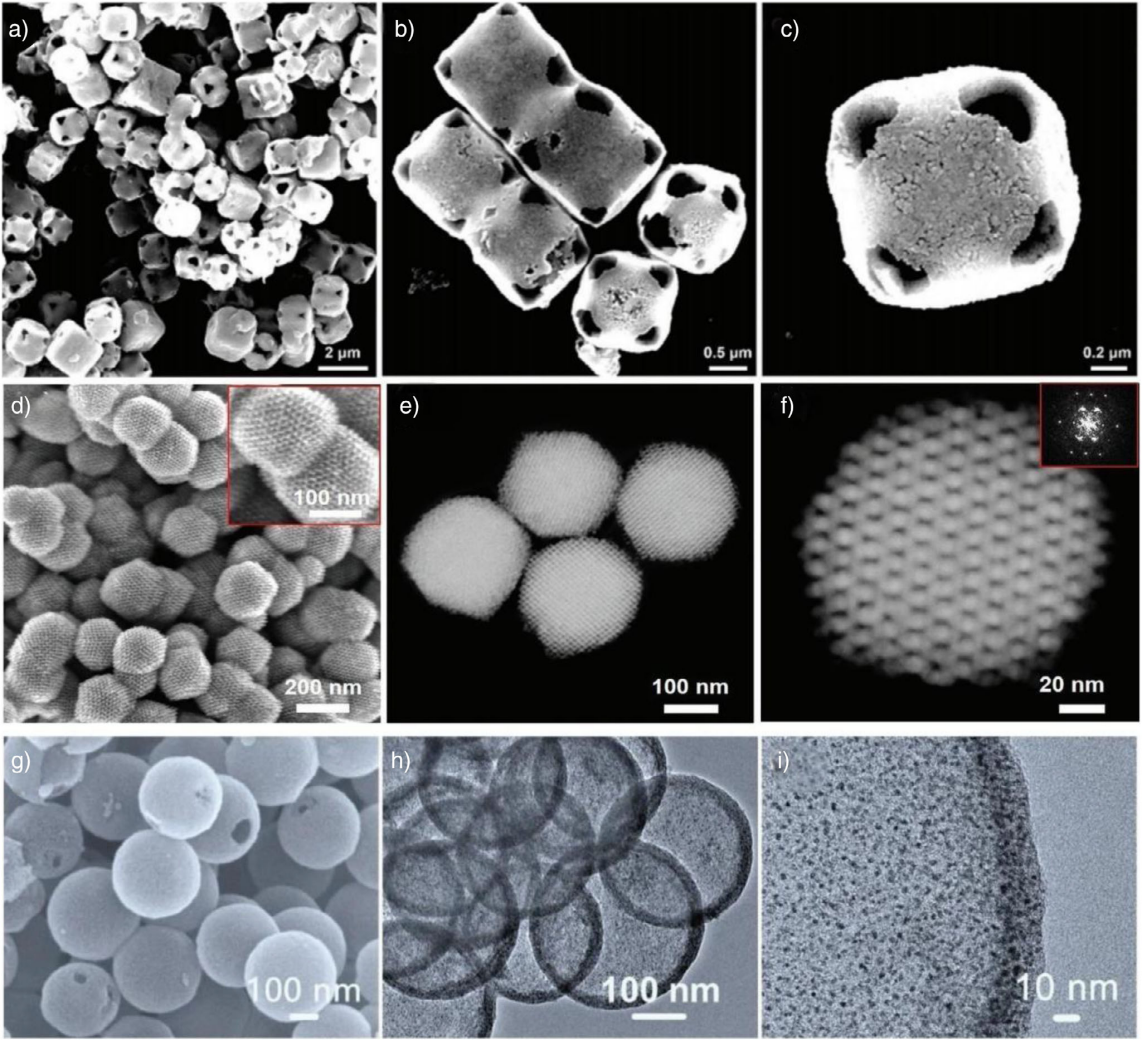
a–c) Morphology and composition of the targeted Co_3_O_4_‐Mo_2_N NFs catalyst. Reproduced from Ref.[122] Copyright 2021, with permission from John Wiley and Sons. d) PXRD patterns of MI–PtZn bimetals and MI–PtZnM trimetals. e) SEM image, f) HAADF‐STEM image. Reproduced from Ref.[123] Copyright 2022, with permission from John Wiley and Sons. g–i) SEM, TEM images. Reproduced from Ref.[131] Copyright 2023, with permission from John Wiley and Sons.

The development of heterostructured catalysts focuses on enhancing HER via precise tuning of interfacial geometry and electronic properties, yet their efficiency is often hindered by restricted single‐particle morphologies that limit active surface/interface exposure and reduce catalytic effectiveness.^[^ ^122^ ^]^ To address this challenge, Wang et al. successfully synthesized a nanoframe‐structured Co_3_O_4_‐Mo_2_N heterointerface catalyst using a controlled pyrolysis method. The catalyst consists of Co_3_O_4_ and Mo_2_N nanoparticles confined within a nanoframe structure. As shown in Figure 10a–c, the Co_3_O_4_‐Mo_2_N nanoframe catalyst retains the nanoframe morphology with uniform shape and size below 1 µm, while exhibiting relatively rough surfaces decorated with numerous small nanoparticles. The images highlight the structural features and material distribution, revealing the uniformity and interaction between Co_3_O_4_ and Mo_2_N within the nanofibers. This design ensures a large surface area with abundant exposed active sites, facilitating efficient catalytic reactions. In a 1.0 M aq. KOH solution, the Co_3_O_4_‐Mo_2_N nanoframe catalyst exhibited excellent HER performance, achieving an overpotential of 100 mV at a current density of 10 mA cm^−2^. The catalyst also demonstrated a significant increase in current density, reaching 61.2 mA cm^−2^ at an overpotential of 300 mV, which is 13.9‐fold higher than that of Co_3_O_4_ nanoframes alone. Additionally, the Tafel slope was measured at 162.4 mV de^−1^, indicating favorable HER kinetics. The exceptional HER performance arises from strong interfacial electron transfer between Mo_2_N and Co_3_O_4_, which balances intermediate adsorption/desorption energies to optimize catalysis. Nanoframe confinement prevents nanoparticle aggregation and preserves structural integrity, significantly enhancing long‐term stability and catalytic activity.

Building on these insights into single‐atom catalysis, the role of nanoconfinement becomes even more critical when exploring bimetallic and trimetallic catalysts. The ordered intermetallic compound platinum–zinc bimetallic (I‐PtZn) constitutes a novel class of alloy electrocatalysts that have emerged for water splitting. Wang et al. successfully developed a trimetallic catalyst, MI–PtZnCo, by using a concurrent template route with a thermally stable KIT‐6 mesoporous template.^[^ ^123^ ^]^ This catalyst, composed of 0D Pt, Zn, and Co atoms embedded in a 3D mesoporous structure, forms a 3D heterostructure. The confined space within the mesoporous structure plays a crucial role in preventing nanoparticle aggregation and enhancing electron transport, leading to improved catalytic performance. Figure 10d shows the powder X‐ray diffraction (PXRD) patterns, revealing the crystalline structures of both compounds. Figure 10e displays an SEM image highlighting surface morphology, while Figure 10f provides a high‐angle annular dark‐field scanning transmission electron microscopy (HAADF‐STEM) image showing atomic‐level elemental distribution. These analyses are essential for understanding the materials' properties and potential applications. In a 1.0 M aq. KOH solution, the MI–PtZnCo catalyst demonstrated outstanding HER activity, with an overpotential as low as 29 mV at a current density of 10 mA cm^−2^. It also exhibited a high mass activity of 1.77 A mg pt^−1^ and a specific activity of 3.07 mA cm pt^−2^, significantly surpassing its bimetallic counterparts. The catalyst retained excellent long‐term stability, with negligible activity loss after 50,000 cycles. The superior HER performance is attributed to strong interfacial interactions and the optimized electronic structure facilitated by the confined space within the mesoporous framework.^[^ ^124^ ^]^ This confinement effect not only prevents the migration and aggregation of metal nanocrystals but also enhances the distribution of active sites and accelerates the overall reaction kinetics, leading to a highly efficient and stable OER catalyst.

Furthermore, the surface characteristics of electrocatalysts play a crucial role in determining their HER catalytic activity.^[^ ^125^ ^]^ In 3D porous catalytic materials, the internal surface properties of 3D channels critically affect HER performance. For instance, Kibsgaard et al. synthesized a highly ordered double‐gyroid MoS_2_ bicontinuous network with uniform, regularly spaced nanochannels (≈7 nm channel‐to‐channel distance; Figure 10g–i)^[^ ^126^ ^]^ Combined with the excellent diffusivity of the 3D channels, the electrolyte can easily reach the inner surfaces of the channels and interact with the numerous active sites on the channel walls.^[^ ^127^ ^]^ Additionally, the catalytic activity of the ordered double‐gyroid MoS_2_ network is influenced by its thickness. As thickness was increased by Mo deposition for 10 s to 1 min, the double‐gyroid MoS_2_ samples exhibited a decreased onset overpotential of 150–200 mV. However, when normalized to surface area, the data show an inverse activity trend, with thinner films demonstrating higher turnover frequencies. The confined space within these 3D channels plays a pivotal role in stabilizing the structure and preventing the aggregation of MoS_2_, which ensures the consistent exposure of active sites and enhances the efficiency of electron transport.

Building on the significance of confined spaces and hierarchical structures to enhance catalytic performance, recent research has also focused on leveraging MOFs and dual atomic sites for further improvements in HER efficiency.^[^ ^128^, ^129^ ^]^ Tao et al. developed a novel 3D porous carbon‐based catalyst (NiCo DASs/N‐C) using a MOF‐assisted host–guest strategy, where nickel and cobalt atoms were encapsulated within the cage‐like framework of ZIF‐8.^[^ ^125^ ^]^ After pyrolysis, this process produced a nitrogen‐doped carbon substrate with uniformly distributed Ni‐N_4_ and Co‐N_4_ diatomic sites. This catalyst exhibited exceptional HER activity, particularly in both 1 aq. M KOH and 0.5 M aq. H_2_SO_4_ solutions. The polarization curves revealed that NiCo DASs/N‐C required significantly lower overpotentials of 189 and 260 mV to reach a current density of 10 mA cm^−2^ under alkaline and acidic conditions, respectively. This performance was superior to that of Ni SAs/N‐C and Co SAs/N‐C, which required much higher overpotentials, demonstrating the enhanced catalytic efficiency of the diatomic site configuration. The Tafel slopes for NiCo DASs/N‐C were 72.5 mV dec^−1^ in 1 aq. M KOH and 82.4 mV dec^−1^ in 0.5 M aq. H_2_SO_4_, indicating efficient HER kinetics. This exceptional HER performance arises from the proximity electronic effect between Ni and Co atomic sites, which modulates the electronic structure of active Co‐N_4_ sites to enhance hydrogen intermediate adsorption and accelerate reaction kinetics. The porous carbon framework's confined space maintains atomic site dispersion and prevents aggregation, ensuring high efficiency and stability in HER applications.

Recent studies show that spherical Ru nanoparticles (NPs) embedded in Ru‐N_x_‐decorated carbon shells exhibit superior HER activity to planar counterparts, highlighting the critical role of substrate geometry in maximizing active site exposure for electrocatalyst design. Luo et al. fabricated an innovative 3D crystalline fullerene network confined electrocatalyst, designated Ru_NP_‐Ru_SA_@CFN‐800, through a solvothermal‐pyrolysis process.^[^ ^23^ ^]^ The synthesis involved the self‐assembly of Ru^3+^, imidazole, and fullerene C_60_ into a 3D structure via a solvothermal approach, followed by pyrolysis, which transformed them into Ru nanoparticles and single atoms confined within the C_60_ lattice. In a 1 M aq. KOH alkaline solution, Ru_NP_‐Ru_SA_@CFN‐800 demonstrated exceptional electrocatalytic hydrogen evolution performance, with an overpotential of only 33 mV at a current density of 10 mA cm^−2^, and exhibited remarkable stability for over 1400 h. Moreover, even under an industrial‐level current density as high as 1000 mA cm^−2^, the catalyst required an overpotential of only 251 mV. RuNP‐Ru_SA_@CFN‐800′s excellent HER performance arises from 3D nanoconfinement, which enhances metal–support electronic interactions to optimize active site electronic structure. This confined environment stabilizes metal species and maximizes catalytic activity, directly boosting HER efficiency.

In the pursuit of enhancing HER efficiency and sustainability in alkaline environments, Wan et al. developed a novel catalyst: a Pt tetrapod encapsulated in an amorphous Ni(OH)_2_ shell (Pttet@Ni(OH)_2_). The design utilizes the synergistic interaction between the Pt core and Ni(OH)_2_ shell. The catalyst showed an overpotential of 196 mV at 10 mA cm^−2^, a Tafel slope of 27 mV dec^−1^, and excellent stability over 10 h.^[^ ^130^ ^]^ These improvements arise from dynamic interfacial engineering, where the Ni(OH)_2_ shell facilitates proton transfer to the Pt surface, optimizing electronic properties and promoting H* adsorption. The interface restructuring creates a proton‐rich environment mimicking acidic HER kinetics, while the Ni(OH)_2_ shell protects the Pt core, enhancing stability. This design demonstrates the potential of dynamic interfacial engineering and confined nanostructures to optimize HER performance in alkaline media.

3D confined space catalysts, such as MXene‐supported RuCo nanoparticles, Ni_2_P‐UNMs/NF, and Ni‐Sn alloy nanoparticles encapsulated in porous carbon shells, enhance the HER by improving electrolyte transport, active site exposure, and electron transfer. The unique 3D nanoconfinement prevents nanoparticle aggregation, stabilizes reaction intermediates, and increases the number of exposed active sites, thus boosting catalytic efficiency and selectivity. DFT studies reveal how the confined spaces optimize electronic structures, stabilize intermediates, reduce energy barriers, and facilitate critical reaction steps, thereby improving HER performance.

In conclusion, this section provides an overview of the development of confined materials for HER in water splitting, emphasizing the distinct advantages of different dimensionalities of confined spaces. These confined spaces are categorized into 1D, 2D, and 3D, each offering unique benefits. 1D confined spaces, such as CNTs are widely utilized for HER due to their ability to encapsulate electrocatalysts, enhancing hydrogen adsorption/desorption and improving catalytic efficiency. In 2D confined spaces, performance is largely dependent on the interlayer spacing. A larger interlayer distance in materials like MXenes facilitates mass transport and increases active site exposure, although it may also affect electron transfer properties for certain catalysts. On the other hand, 3D confined spaces combine various dimensional confined spaces or varying sizes, providing enhanced mass transport and allowing for efficient hydrogen gas bubble escape, which leads to improved HER performance and dynamic stability.^[^ ^116^ ^]^

## 5 Electrocatalysts for OER in Confined Space

To achieve efficient OER catalysis, the ideal catalysts must demonstrate not only low overpotentials but also superior stability, both of which are crucial for sustaining long‐term performance in energy conversion applications. Enhancing the ECSA and increasing the density of active sites through nanostructural engineering are fundamental strategies to boost catalytic performance. However, while these advancements improve reaction efficiency, they are often hindered by the challenges of maintaining catalyst integrity during prolonged operational periods, which can lead to structural degradation. OER catalysts currently encompass a wide range of materials, including transition metal oxides, hydroxides, and phosphides. Although these materials are more cost‐effective than noble metals, they still face significant hurdles in terms of durability and stability under real‐world conditions.^[^ ^48^ ^]^

In recent years, the development of nanoconfinement techniques has shown great promise in improving both catalytic activity and long‐term stability for OER catalysts. The subsequent section presents a comprehensive overview of recent advancements in OER catalysts, classified according to dimensionality: 1D, 2D, and 3D architectures. A systematic discussion and summary of these catalysts alongside their critical properties are outlined in Table 2, emphasizing their performance advantages, technical challenges, and feasibility for industrial‐scale implementation.

**Table 2:** Properties of 1D‐3D confined electrocatalysts for OER.

Electrocatalysts	Dimension	Overpotential at 10 mA cm^−2^ (mV vs RHE)	Tafel slope (mV dec^−1^)	Electrolyte	Stability (h)	Ref.
NiSe@CNTs	1D	145	10.5	1 M aq. KOH	730	[137]
Ni/NiO@Ru‐NC	1D	261	78	1 M aq. KOH	100	[138]
Ir‐IrO_x_/C	2D	198	106.3	0.5 M aq. H_2_SO_4_	18	[146]
NiFe‐BTC//G	2D	106	55	1 M aq. KOH	150	[148]
Cu_2_S/Ni_3_S_2_@Ni‐BDC	2D	353	75	1 M aq. KOH	12	[152]
FeNi@NCSs	2D	318	40.8	1 M aq. KOH	–	[162]
Fe‐ZnMOFS/NF	3D	147	34	1 M aq. KOH	800	[157]
Fe‐MOF@MoS_2_‐6h	3D	187	61	1 M aq. KOH	100	[147]
Fe_.92_Co_.08_S@SC/SCWF	3D	238	35.9	1 M aq. KOH	100	[158]
Ru/TiO_x_	3D	174	45.6	0.5 M aq. H_2_SO_4_	–	[163]
PCO‐nHI	3D	171	–	1 M aq. KOH	100	[161]
Ni_0.25_Cu_0.75_/C	3D	400	80	0.5 M aq. NaOH	–	[119]

### 5.1 Electrocatalysis in 1D Channels

1D nanomaterials, including nanorods, nanotubes, nanowires, and nanofibers, have attracted significant attention in electrocatalysis because they provide inherent advantages compared with nanoparticles.^[^ ^19^, ^132^ ^]^ They have high aspect ratios, large specific surface areas, a high density of surface unsaturated atoms, and high electron mobility. These distinctive features provide significant advantages in surface‐related applications.^[^ ^19^, ^132^ ^]^ Hollow CNTs exhibit higher specific surface area and superior mass transport properties compared to other structures, making them promising supports for electrochemical applications.^[^ ^133^ ^]^ Additionally, CNTs benefit from the typical advantages of carbon materials, such as abundant availability, low cost, and strong resistance to both acidic and alkaline environments.^[^ ^134^ ^]^ Ding and colleagues successfully fabricated Co single atoms confined within a polymerized ionic liquid on multiwalled CNTs, which demonstrated superior OER performance compared to catalysts containing Co particles. The CNTs not only served as the support for Co SA‐PIL but also enhanced the overall conductivity of the catalysts.^[^ ^19^, ^135^ ^]^

**Figure 11:**
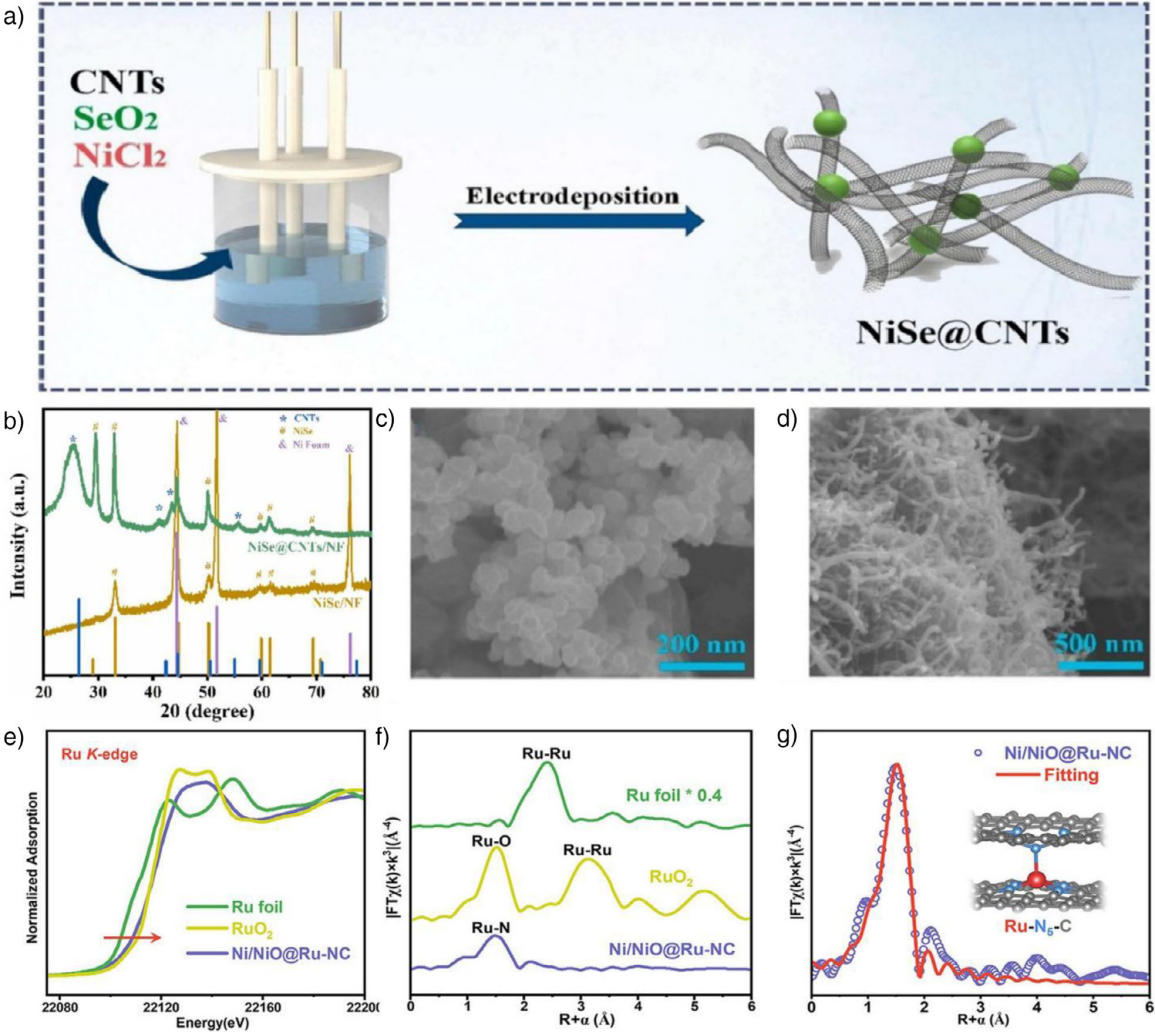
a) Schematic representation of the synthesis process for NiSe@CNTs. b) XRD patterns of both NiSe and NiSe@CNTs. c–d) SEM images of NiSe. Reproduced from Ref.[137] Copyright 2023, with permission from Elsevier. e) Ru K‐edge XANES spectra of Ni/NiO@Ru‐NC in comparison with Ru foil and RuO_2_ standards. f) Fourier‐transformed EXAFS spectra of the same samples, illustrating differences in local structure. g) EXAFS fitting curve of Ni/NiO@Ru‐NC in R space; the inset displays the DFT‐optimized Ru–N_5_‐C coordination geometry. Reproduced from Ref.[138] Copyright 2024, with permission from Elsevier.

In addition to PIL, Liu and co‐workers employed g‐C_3_N_4_ to anchor Ni/Fe atomic sites, using CNTs as a support. The resulting bimetal single‐atom catalyst exhibited remarkable OER activity, with a low overpotential of approximately 326 mV at 10 mA cm^−2^.^[^ ^136^ ^]^ Xue et al. developed a NiSe@CNTs heterostructure catalyst via electrodeposition, combining nickel selenide (NiSe) nanoparticles with CNTs (Figure 11a).^[^ ^137^ ^]^ DFT calculations further elucidated the underlying mechanism by which the confinement effect in the CNTs enhances the catalytic performance. Specifically, the DFT studies indicated that the metal‐Nx structure formed by the Ni and Fe atoms embedded in g‐C_3_N_4_ significantly altered the electronic properties of the catalyst, optimizing the adsorption energy for OER intermediates. This electronic structure modification facilitated a more efficient electron transfer during the OER process, thus contributing to the catalyst's superior performance. Additionally, DFT simulations revealed that the interfacial interaction between the CNTs and the metal sites not only prevented the aggregation of nanoparticles but also stabilized the active sites, ensuring their high dispersion and preventing deactivation under harsh electrochemical conditions. X‐ray diffraction (XRD) confirmed the successful formation of the composite, showing characteristic peaks for both NiSe and CNTs (Figure 11b). SEM images revealed that the confined environment provided by CNTs effectively prevented the aggregation of NiSe nanoparticles, significantly reducing their size to 10–15 nm and exposing more active sites (Figure 11c,d). This confinement not only minimized particle aggregation but also enhanced the dispersion of the active sites, leading to improved catalytic efficiency. X‐ray absorption spectroscopy (XAS) further demonstrated enhanced electronic interactions within the heterostructure. The Ni K‐edge spectrum of NiSe@CNTs showed a shift to lower energy, indicating a reduced valence state of Ni due to the interfacial effects with CNTs. Similarly, the Se K‐edge spectrum shifted closer to that of Se foil, suggesting electron gain and further interaction enhancement. This interaction led to a contraction of the Ni─Se bond, as confirmed by Fourier transform EXAFS analysis. In a 1.0 M aq. KOH alkaline solution, the optimized NiSe@CNTs exhibited remarkably high activity, with an overpotential of only 145 mV for the OER at 100 mA cm^−2^, along with outstanding long‐term stability exceeding 730 h. The superior performance of this catalyst is attributed to the introduction of CNTs, which not only prevent nanoparticle aggregation through spatial confinement but also minimize particle size, thereby increasing the number of exposed Ni active sites.

Nanoconfinement in bimetallic systems enhances the catalytic performance and stability of OER catalysts. Similar strategies are applied to optimize OER catalysts by introducing confined spaces within nanostructured supports. CNT arrays improve both efficiency and durability of electrocatalysts for OER. To address stability challenges of 1D carbon materials, researchers have developed heterostructured catalysts, combining active metal and metal oxide nanoparticles within the carbon matrix to leverage nanoconfinement's synergistic effects. Quan et al. developed a novel bifunctional electrocatalyst, Ni/NiO@Ru‐NC, through a one‐step liquid‐assisted chemical vapor deposition (LCVD) process.^[^ ^138^ ^]^ This catalyst features Janus Ni/NiO nanoparticles and single‐atom Ru sites confined within the sidewalls and apex of nitrogen‐doped CNT arrays, forming a heterostructure. DFT calculations offer important theoretical insights into this design, revealing that the confinement effect within the nitrogen‐doped CNTs is crucial for stabilizing the active metal sites, specifically the Ni and Ru atoms, and optimizing their electronic properties. The CNT confinement enhances the interaction between these active sites and the reaction intermediates, which is essential for facilitating the OER. In a 1 M aq. KOH solution, the Ni/NiO@Ru‐NC catalyst demonstrated excellent OER performance, achieving low overpotentials of 261 and 318 mV at current densities of 100 and 500 mA cm^−2^, respectively. This is consistent with DFT predictions, which suggest that the confined environment lowers the energy barriers for the adsorption and desorption of intermediates, promoting faster electron transfer and improving overall reaction kinetics. To elucidate the local coordination environment and electronic structure of the Ru species in Ni/NiO@Ru‐NC, X‐ray absorption spectroscopy (XAS) was conducted, as shown in Figure 11e–g. Figure 11e shows that the XANES spectrum of Ni/NiO@Ru‐NC at the Ru K‐edge lies between those of Ru foil and RuO_2_, indicating that Ru atoms possess a partial positive valence state (+*δ*, where 0 < *δ* < 4). As presented in Figure 11f, the Fourier‐transformed *k^3^*‐weighted EXAFS spectrum of Ni/NiO@Ru‐NC displays a prominent peak at around 1.5 Å, corresponding to the Ru–N coordination shell. Notably, no Ru–Ru scattering features are observed at 2.4 or 3.1 Å, confirming that Ru atoms are atomically dispersed rather than aggregated into metallic Ru or RuO_x_ nanoparticles. Figure 11g shows the EXAFS fitting results, which reveal a coordination number of approximately 4.8 for Ru–N. This suggests that the dominant coordination environment of Ru in the catalyst is likely a Ru–N_5_ configuration, potentially consisting of a planar Ru–N_4_ unit axially coordinated with a pyridinic nitrogen, forming a pyramidal geometry (as illustrated in the inset of Figure 11g). The DFT results also support the experimental finding that the confined environment prevents the aggregation of active metal nanoparticles, which typically leads to loss of catalytic activity over time. The good OER performance is attributed to the synergistic effect between the Janus Ni/NiO nanoparticles and the Ru single atoms, which together enhance electron transfer and increase the density of active sites. The multiscale confinement within the CNT arrays ensures uniform dispersion of the active species and stabilizes the catalyst structure, thereby optimizing the catalytic environment and significantly improving the overall efficiency and durability of the catalyst.

In the search for high‐performance and sustainable electrocatalysts for the OER in electrochemical water splitting, Townsend et al. developed a novel catalyst system: metal oxide nanoparticles encapsulated within single‐walled carbon nanotubes (MO_x_@SWNTs) to enhance OER performance through synergistic interactions between the metal oxide core and the carbon shell. The catalyst showed excellent activity, with an overpotential of 417 mV at 10 mA cm^−2^ for IrO_2_@SWNT, a Tafel slope of 100 mV dec^−1^, and stability over 2000 cycles.^[^ ^139^ ^]^ These improvements are due to dynamic interfacial engineering, where electron transfer from the carbon nanotubes to the metal oxide alters the interface, promoting ^−^OH adsorption and accelerating the OER rate. The confined environment within the SWNTs enhances stability by protecting the metal oxide from direct contact with the electrolyte. The in‐situ Raman spectroscopy indicates electron transfer from the carbon support to the encapsulated lrO_2_, leading to a more positively charged carbon surface. This charge redistribution enhances the binding of ^−^OH, aligning with Tafel analysis results and the improved OER activity of confined lrO_2_@SWNTs. The *ex‐situ* XPS analysis suggests that the oxidation of the blocked material takes place at relatively higher overpotentials. The DFT calculations indicate that the electronic structure and morphology of the encapsulated lrO_2_ significantly influences charge transfer behavior and charge‐transfer resistance during OER, highlighting the potential of dynamic interfacial engineering in optimizing electrocatalytic performance.

In the quest to enhance the efficiency and stability of OER electrocatalysts in acidic environments, Chen et al. developed a novel 1D catalyst: a bicontinuous nanoreactor composed of multiscale defective RuO_2_ nanomonomers (MD‐RuO_2_‐BN), designed to enhance OER efficiency and stability in acidic environments. The catalyst exhibited exceptional performance, with an overpotential of 196 mV at 10 mA cm^−2^, a Tafel slope of 100 mV dec^−1^, and stability over 24 h.^[^ ^140^ ^]^ These improvements are attributed to the synergistic effects of multiscale defects, including oxygen and ruthenium vacancies, and the confined environment created by the nanoreactor. The in‐situ Raman spectroscopy confirms that these defects weaken the Ru–O interaction, preventing oxidation and dissolution of high‐valence Ru species. DFT calculations revealed that these defects lower the energy barrier for the rate‐determining step, particularly at the twin boundary Ru sites, reducing the overpotential to 0.22 V. The confined nanoreactor structure also enhances stability by protecting the RuO_2_ from direct electrolyte contact, ensuring sustained catalytic activity.

In the pursuit of enhancing the efficiency and stability of electrocatalysts for the OER in acidic media, Lv et al. developed a highly active Ru/TiO_x_ catalyst using the synergistic interaction between ruthenium nanoparticles and a nonstoichiometric titanium oxide support, grown in‐situ on titanium foam. The catalyst showed outstanding performance, with overpotentials of 174 mV at 10 mA cm^−2^ and 265 mV at 500 mA cm^−2^, a Tafel slope of 45.6 mV dec^−1^, and remarkable stability, maintaining activity for over 900 h at 10 mA cm^−2^.^[^ ^141^ ^]^ These improvements result from dynamic interfacial engineering and atomic‐level confinement. The Ru–TiO_x_ interaction induces charge redistribution, stabilizing Ru sites, and preventing over‐oxidation. The TiO_x_ growth on Ti foam provides a confined environment that protects Ru, optimizing its electronic properties for efficient OER. The combined effects of dynamic interface restructuring and atomic‐level confinement enhance the catalyst's performance, showcasing the potential of these strategies in advanced electrocatalyst design.

1D confined structures in electrocatalysts, such as CNTs, enhance OER performance by providing high surface area, improved electron mobility, and excellent stability. Confining single atoms or metal nanoparticles within CNTs improves electron transfer, prevents aggregation, and boosts OER efficiency. DFT calculations show that confinement optimizes metal‐site interactions, stabilizes active sites, and enhances intermediate adsorption. However, 1D materials face scalability issues, uniformity challenges, and difficulty tuning catalytic sites. These limitations drive interest in 2D materials, which offer tunable properties, better accessibility for intermediates, and improved scalability, making 2D OER catalysts a promising alternative.^[^ ^116^ ^]^

### 5.2 Electrocatalysis in 2D Layered Spaces

The unique properties of 2D materials, such as their thinness and large surface area, make them highly suitable for OER catalysis. These materials exhibit high intrinsic catalytic activity, which is further enhanced by their ability to facilitate efficient electron transfer and the adsorption of reaction intermediates. The 2D structure not only optimizes the catalyst's interaction with the adsorbed species but also allows for easy manipulation of the electronic properties, resulting in improved catalytic performance.^[^ ^115^ ^]^ Additionally, 2D materials, particularly those incorporating TMDs or metal hydroxides, are excellent candidates for high‐performance electrocatalysis. Moreover, the confinement effect in 2D nanoconfined spaces, which are gaps between adjacent surfaces, plays a crucial role in enhancing catalytic activity. By engineering layered materials, such as MOFs or graphene, to create confined environments, the interlayer spacing can be finely tuned. This precise control over the nanoconfined space dimensions helps maintain a high dispersion of active sites, reducing the need for precious metals and significantly improving the overall efficiency of the catalyst. This combination of enhanced catalytic properties and efficient use of materials makes 2D nanoconfined structures a promising approach in OER catalysis.^[^ ^12^, ^88^ ^]^

**Figure 12:**
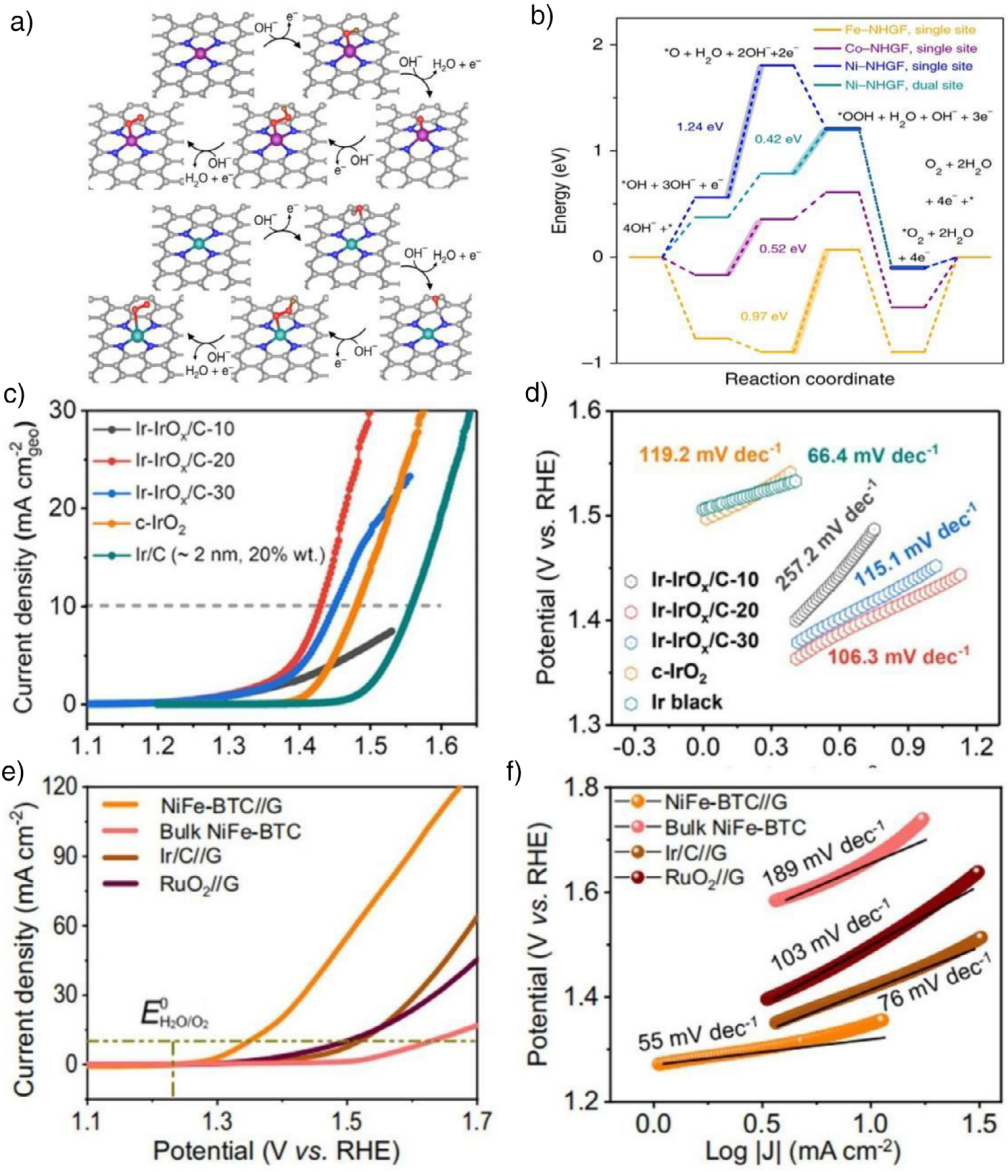
a) Proposed reaction pathways featuring optimized intermediate geometries for OER: single‐site mechanism and dual‐site mechanism. b) Free energy diagrams at 1.23 V for OER on Fe‐NHGF, Co‐NHGF, and Ni‐NHGF with single‐site mechanisms, and Ni‐NHGF with a dual‐site mechanism. Highlighted are the rate‐determining steps, with the limiting energy barrier values indicated. c) Water oxidation polarization curves measured with a linear potential scan at 2 mV s^−1^ for Ir–IrO_x_/C, commercial crystalline c‐IrO_2_, Ir/C, and carbon black, with the current normalized to the geometric surface area. d) Tafel plots comparing Ir–IrO_x_/C, commercial c‐IrO_2_, and Ir/C. Reproduced from Ref.[146] Copyright 2022, with permission from American Chemical Society. e) LSV curves for OER obtained at 10 mV s^−1^ in 1.0 M aq. KOH using NiFe‐BTC//G, bulk NiFe‐BTC, commercial Ir/C, and RuO_2_, with Ag/AgCl as the reference electrode. f) Tafel plots comparing NiFe‐BTC//G, bulk NiFe‐BTC, commercial IrO_2_, and RuO_2_. Reproduced from Ref.[148] Copyright 2022, with permission from Springer Nature.

In addition to these general principles, specific examples of 2D materials with confined atoms demonstrate even more remarkable OER catalytic performance. For instance, graphene that confines single nitrogen atoms can function as a metal‐free catalyst for OER. Graphene that confines single nitrogen atoms can function as a metal‐free catalyst for the OER.^[^ ^107^, ^142^ ^]^ The carbon atom adjacent to pyridinic nitrogen has been identified as the active site. A recent study by Fei et al. revealed that embedding single Ni atoms into graphene lattices with pyridinic nitrogen ligands activates neighboring carbon atoms, forming a cooperative site critical for enhancing OER catalytic activity (Figure 12a).^[^ ^107^, ^143^ ^]^ In addition to nickel, Fe and Co single atoms confined in graphene were synthesized for comparative study (Figure 12b). X‐ray absorption spectroscopy revealed their atomic structures all adopt the MN_4_C_4_ configuration. OER activity followed the order Ni > Co > Fe. DFT calculations identified two possible reaction pathways: a single‐site mechanism (metal center‐only) and a dual‐site mechanism (metal–carbon cooperative). The dual‐site pathway effectively lowers the energy barrier, providing a notable advantage over the single‐site approach by optimizing reaction kinetics and enhancing catalytic efficiency. In alkaline media, Ni single atoms confined within defective graphene, without nitrogen ligands, also exhibit high catalytic activity for the OER. This catalyst shows superior activity compared to graphene containing NiN_4_ centers, as previously reported. At a current density of 10 mA cm^−2^, the overpotential was measured at 270 mV, which is better than that of IrO_2_. DFT calculations reveal that the local defect structure in graphene significantly influences the electronic structure of the Ni atoms, indicating that the most effective configuration for OER involves Ni atoms confined within the divacancy defects of graphene.^[^ ^107^, ^143^ ^]^

Although 2D catalysts with confined atoms show impressive performance, Ir‐based materials like IrO2, metallic Ir, and IrOx remain the benchmark for OER due to their exceptional activity and durability, along with outstanding corrosion resistance in acidic environments.^[^ ^144^, ^145^, ^146^ ^]^ Zu et al. successfully synthesized a 2D Ir‐IrO_x_/C catalyst with an ordered mesoporous interlayer space using a nanoconfined self‐assembly strategy.^[^ ^146^ ^]^ The catalyst consists of uniformly distributed ultrafine Ir–IrO_x_ nanoparticles (∼2 nm) embedded within the mesoporous layered structure, forming a 2D heterostructure. In 0.5 M aq. H_2_SO_4_ acidic solution, the Ir–IrO_x_/C nanosheets exhibited an overpotential of only 198 mV at a current density of 10 mA cm^−2^ and a Tafel slope of 106.3 mV dec^−1^, indicating rapid OER kinetics (Figure 12c,d). After 18 h of stability testing, the catalyst maintained nearly 100% of its initial current density, indicating excellent durability. Its high‐performance stems from synergistic effects among mixed‐valence components, high surface area, open interlayer channels, and a unique oxygen coordination environment, which together promote water activation and O─O bond formation. The nanoconfined self‐assembly strategy ensures precise nanoparticle confinement, preventing aggregation and enhancing dispersion, active site exposure, and electron/ion transport. Additionally, ordered channels improve catalyst‐reactant contact, accelerating reaction kinetics. The DFT simulations further confirm that the ordered nanostructural channels optimize the electron and ion transport pathways, ensuring enhanced catalytic efficiency.

MOFs are recognized as promising candidates for OER applications due to their large surface area, customizable porosity, adjustable compositions, and diverse metal centers.^[^ ^147^ ^]^ However, their inherently poor electrical conductivity and limited stability significantly hinder their effectiveness in water oxidation processes, necessitating the development of solutions to address these challenges. Lyu et al. employed a strategy that confines poorly conductive MOFs between graphene multilayers, resulting in a significant enhancement of their catalytic performance.^[^ ^148^ ^]^ The resultant NiFe‐BTC//G catalyst, which was synthesized via an electrochemical process, exhibited an impressively low overpotential of approximately 106 mV at a current density of 10 mA cm^−2^ in 1.0 M aq. KOH, as demonstrated in Figure 12e. DFT calculations further elucidate the role of the nanoconfinement provided by the graphene multilayers. These calculations reveal that the confinement enhances the electronic structure of the Ni and Fe centers in the MOF, facilitating more efficient electron transfer. Specifically, DFT simulations show a more favorable electronic interaction between the Ni/Fe 3d orbitals and the O 2p orbitals, which results in stronger binding of O intermediates, promoting the OER kinetics. This performance is notably superior compared to bulk NiFe‐BTC and commercial catalysts like Ir/C and RuO_2_, which showed higher overpotentials of 399, 287, and 267 mV, respectively. Additionally, the Tafel slope of the NiFe‐BTC//G catalyst was significantly lower, at 55 mV dec^−1^, compared to bulk NiFe‐BTC (189 mV dec^−1^), Ir/C (76 mV dec^−1^), and RuO_2_ (103 mV dec^−1^), indicating more favorable electrocatalytic kinetics for the OER (Figure 12f). EIS further confirmed the catalyst's efficiency, with the NiFe‐BTC//G showing the smallest charge transfer resistance (*R*_ct_∼0.46 Ω) among the tested materials. Moreover, the NiFe‐BTC//G catalyst demonstrated remarkable stability, maintaining its catalytic activity at a current density of 10 mA cm^−2^ for over 150 h. This long‐term stability, combined with the enhanced performance metrics, underscores the effectiveness of the graphene multilayer confinement strategy for improving the electrocatalytic activity and durability of MOF‐based catalysts.

In a similar vein, nickel‐based MOFs have garnered significant attention as OER catalysts due to their unique structural characteristics and abundant active metal sites. However, their practical performance is often constrained by suboptimal electrical conductivity, limited accessible reactive sites, and reduced catalytic activity. To address these challenges, strategies such as engineering the electrode–electrolyte interface, utilizing 2D nanosheet architectures, and integrating metal sulfide nanoparticles have been successfully employed.^[^ ^149^, ^150^, ^151^ ^]^ The synthesis of 2D heterostructures, merging the properties of quantum dots with those of nanosheets, has been found to be an especially beneficial approach. For example, Jiang et al. successfully synthesized a heterostructured catalyst named Cu_2_S/Ni_3_S_2_@Ni‐BDC using a hydrothermal method followed by electrodeposition.^[^ ^152^ ^]^ The catalyst consists of 0D Cu_2_S and Ni_3_S_2_ nanoparticles embedded within 2D Ni‐BDC nanosheets, which are grown in‐situ on a nickel foam substrate, forming a 2D heterostructure. DFT calculations offer critical insights into the electronic interactions between the Cu_2_S and Ni_3_S_2_ nanoparticles, revealing that the confined environment within the 2D Ni‐BDC nanosheets enhances the electronic properties of these metal sulfides. Specifically, DFT simulations show that the nanoparticle dispersion within the 2D matrix promotes favorable interactions between Cu_2_S and Ni_3_S_2_, leading to improved charge transfer efficiency and lowering the energy barriers for the OER reaction. In 1 M aq. KOH solution, the as‐prepared Cu_2_S/Ni_3_S_2_@Ni‐BDC catalyst exhibited remarkable OER performance, with an overpotential of 353 mV at a current density of 50 mA cm^−2^. The Tafel slope was measured at 75 mV dec^−1^, indicating rapid OER kinetics. Additionally, the catalyst demonstrated excellent stability, retaining over 96% of its initial activity after 12 h of continuous operation. The confinement within the Ni‐BDC nanosheets is crucial, as DFT calculations confirm that the confined space prevents nanoparticle aggregation, which is typically detrimental to catalyst stability. This confinement effect, combined with the synergistic interaction between Cu_2_S and Ni_3_S_2_, optimizes the catalytic environment, enhancing the overall efficiency of the OER process. The outstanding catalytic activity is primarily attributed to the synergistic effect between Cu_2_S and Ni_3_S_2_ nanoparticles and the unique structure of the 2D Ni‐BDC nanosheets, which provides abundant active sites and enhances electron transport. The confined space within the 2D nanosheets plays a critical role in maintaining the dispersion of nanoparticles, preventing their aggregation, and optimizing the catalytic environment, which significantly enhances OER performance.

The search for cost‐effective yet efficient catalysts has led to the exploration of transition metal alloys, such as CoFe and CoNi, which offer promising improvements in catalytic activity through enhanced electrical conductivity and synergistic metal effects.^[^ ^153^, ^154^ ^]^ However, the rising costs of these metals have driven the need for strategies that can reduce their usage and provide more economical alternatives. Zhang et al. developed an efficient OER catalyst: ultrathin confined PbTiO_3_ nanosheets. The catalyst showed exceptional performance, with an overpotential of 250 mV at 10 mA cm^−2^, a Tafel slope of 45 mV dec^−1^, and stability over 100 h.^[^ ^155^ ^]^ These attributes are attributed to synergistic interactions within confined nanostructures and surface reconstruction driven by interfacial charge modulation. During OER, PbTiO_3_ nanosheets undergo dynamic reconstruction, optimizing oxygen intermediate adsorption through charge transfer and electron density modulation at the interface. The confinement of nanosheets within a specific orientation improves water stability. The interaction between confined PbTiO_3_ and the reconstructed surface generates a dual‐site effect, boosting catalytic efficiency, stability, and durability for practical applications.

In the development of high‐performance and sustainable electrocatalysts for the OER in water splitting, Tang et al. developed a high‐performance Janus dual‐atom catalyst (FeCo‐N_3_O_3_@C) for the OER in water splitting, utilizing the synergistic interaction between Fe and Co atoms within a confined environment. The catalyst showed excellent performance, with an overpotential of 0.298 V at 10 mA cm^−2^ and a Tafel slope of 55 mV dec^−1^.^[^ ^156^ ^]^ This improvement is due to dynamic interfacial engineering. Ar plasma treatment induced surface reconstruction, forming defect‐rich d‐FeN_3_@C and providing active sites for Co–O_3_ moieties, which enhanced charge transfer between Fe and Co atoms. X‐ray absorption spectroscopy (XAS) and X‐ray emission spectroscopy (XES) confirmed this synergy. The confined carbon substrate further optimized the electronic properties and mass transfer, boosting OER efficiency.

Confined 2D nanomaterials in electrocatalysts enhance OER performance by optimizing electron transfer, preventing nanoparticle aggregation, and improving active site dispersion. Materials like 2D MOFs or graphene with controlled interlayer spacing show improved catalytic activity. DFT simulations reveal that confinement alters the electronic structure of metal sites, lowering energy barriers and enhancing intermediate adsorption for efficient OER.^[^ ^115^ ^]^ Confined single atoms like Ni within graphene enhance OER activity by modulating nearby carbon atoms' electronic properties. In metal sulfides, confinement improves charge transfer efficiency and lowers energy barriers for OER. It also stabilizes catalysts by preventing nanoparticle growth during prolonged use. These innovations, supported by theoretical insights, result in catalysts with low overpotentials, fast kinetics, and long‐term stability, making confined 2D nanostructures a promising strategy for improving OER efficiency and durability.^[^ ^18^ ^]^

Despite their high catalytic activity, the long‐term stability of 2D OER catalysts is a challenge. The high reactivity of active sites leads to surface reconstruction and degradation under OER conditions, due to the high surface energy of 2D materials. Their limited thickness also hinders ion diffusion, reducing stability during prolonged cycling. As a result, there is increasing interest in 3D OER catalysts, which may offer better stability through a more robust structural framework, preventing dissolution and degradation while maintaining high catalytic performance.

### 5.3 Electrocatalysis in 3D Confined Spaces

3D nanoconfinement enhances OER catalyst performance by providing a more versatile environment than 1D and 2D confinement, improving reactant diffusion, intermediate adsorption, and molecular interactions. The larger spatial dimensions increase active site accessibility and transport efficiency, leading to better reaction kinetics and catalytic efficiency. 3D confinement stabilizes active sites, preventing aggregation and ensuring uniform dispersion for long‐term stability. Designs like mesoporous frameworks, spherical templates, and hierarchical architectures optimize pore size, surface chemistry, and interfacial interactions, boosting electron and ion transfer. Thus, 3D nanoconfinement offers a promising path for high‐performance, durable OER catalysts.^[^ ^116^ ^]^

**Figure 13:**
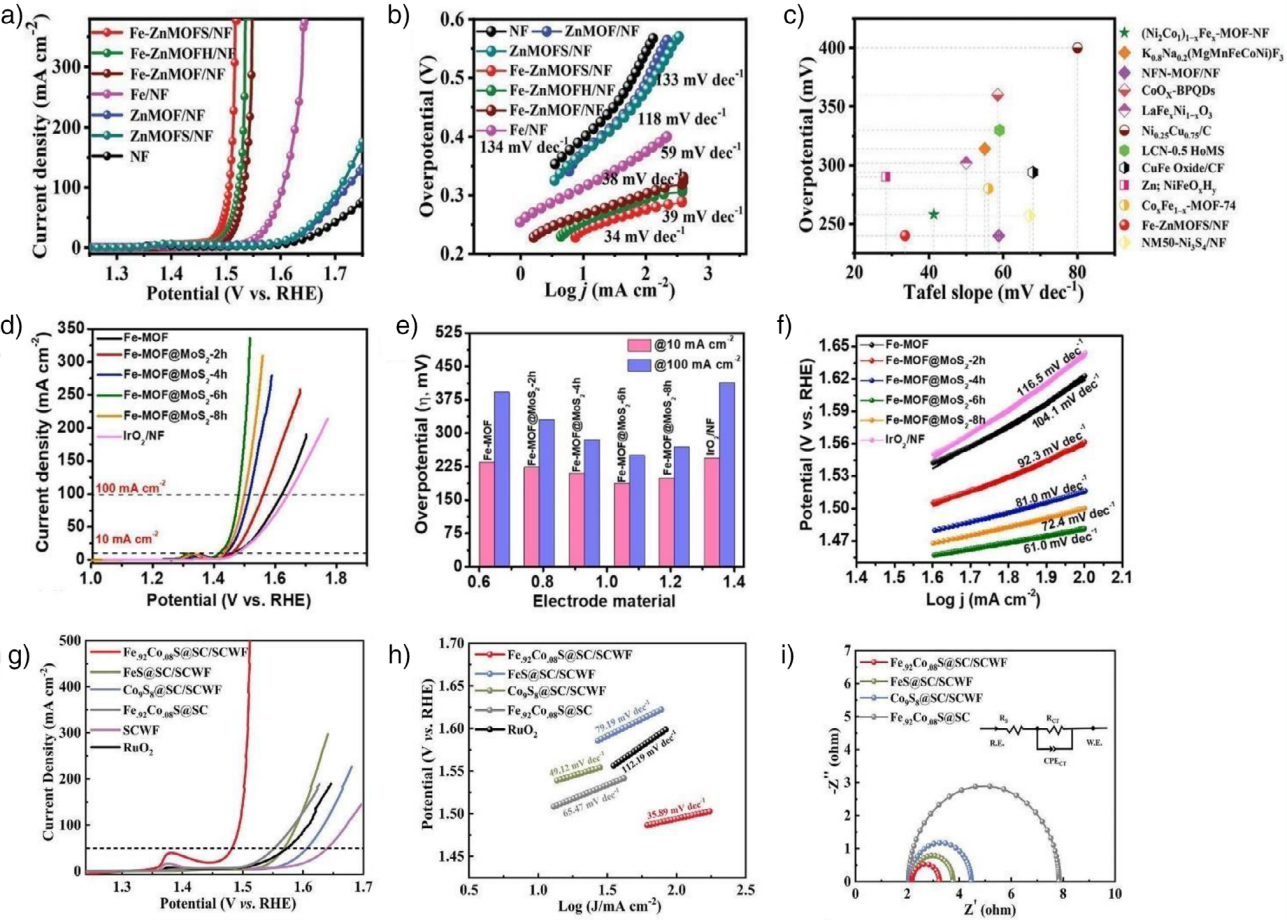
a) LSV curves of various electrocatalysts measured in an O_2_‐saturated 1.0 M aq. KOH solution. b) The corresponding Tafel plots derived from the LSV data. c) Comparison of oxygen evolution performance with other reported electrocatalysts. Reproduced from Ref.[157] Copyright 2022, with permission from Royal Society of Chemistry. d) LSV polarization curves of Fe‐MOF, Fe‐MOF@MoS_2_‐2 h, Fe‐MOF@MoS_2_‐4 h, Fe‐MOF@MoS_2_‐6 h, Fe‐MOF@MoS_2_‐8 h, and IrO_2_/NF. e) Overpotential of the electrocatalysts at current densities of 10 and 100 mA cm^−2^. f) Tafel plots for the electrocatalysts. Reproduced from Ref.[147] Copyright 2023, with permission from Elsevier. g) LSV curves for Fe_.92_Co_.08_S@SC/SCWF, FeS@SC/SCWF, Co_9_S_8_@SC/SCWF, Fe_.92_Co_.08_S@SC, SCWF, and commercial RuO_2_. h) The corresponding Tafel slopes. i) Nyquist plots of Fe_.92_Co_.08_S@SC/SCWF, FeS@SC/SCWF, Co_9_S_8_@SC/SCWF, and Fe_.92_Co_.08_S@SC in 1.0 M aq. KOH, with the inset showing the corresponding equivalent circuit. Reproduced from Ref.[158] Copyright 2024, with permission from Elsevier.

MOFs are gaining prominence in catalysis due to their porous crystalline structure, compositional control, customizable pore structures, and high surface areas. These features offer significant opportunities for various catalytic applications. MOF derivatives provide versatility in adjusting composition and structure, optimizing overall performance.^[^ ^157^ ^]^ Wang and colleagues synthesized a novel 3D porous nanorod array electrocatalyst (denoted as Fe‐ZnMOFS/NF when placing the electrode into static water) through an in‐situ confined interface transformation and postmetalation strategy, with a 2D nickel foam serving as the substratectrode, prepared via electrochemical deposition, underwent an interface transformation in water and nitrogen atmosphere, resulting in ZnMOFS nanorods decorated with Fe‐BTC nanodots. The XAS analysis was performed to monitor the structural and electronic changes of the Fe‐ZnMOFS/NF catalyst during the OER process. The results revealed that the Fe oxidation state increased slightly during the reaction. This indicates dynamic changes in the electronic structure that are crucial for maintaining high catalytic activity. Additionally, the FTIR spectroscopy was employed to probe the intermediates involved in the OER process. The results showed characteristic peaks corresponding to the adsorption and desorption of oxygen intermediates. This provides direct evidence of the optimized binding energy for intermediate formation on the Fe‐ZnMOFS/NF surface. DFT calculations provide important insights into the electronic structure and catalytic behavior of the Fe‐ZnMOFS/NF catalyst. Specifically, DFT simulations reveal that the Fe‐BTC nanodots optimize the adsorption energy of oxygen intermediates and enhance electron transfer efficiency during the OER. Figure 13a displays the LSV curves of various electrocatalysts, measured in an O_2_‐saturated 1.0 M aq. This 3D heterostructured catalyst exhibited outstanding OER activity in 1.0 M aq. KOH solution, with an overpotential as low as 240 mV and a Tafel slope of 34 mV dec^−1^, and retained its performance over 800 h of stability testing (Figure 13b). KOH solution, highlighting the OER performance of the electrocatalysts and providing a comparison of their electrochemical activity under the same experimental conditions. Additionally, Figure 13c compares the oxygen evolution performance of the studied electrocatalyst with various reported electrocatalysts, demonstrating the superior activity of the current catalyst with its lower overpotential and higher efficiency in driving the OER under similar conditions. This remarkable performance is ascribed to the nanoconfinement effect within the catalyst, which enhances the accessibility of active sites and improves mass and charge transfer efficiency. Specifically, the interfacial etching and in‐situ precipitation processes led to the formation of Fe‐BTC nanodots, optimizing the binding energy of intermediates, while uncoordinated carboxylate groups acted as proton transfer relays, accelerating the proton transfer process and markedly improving the OER activity.

In another study, R. Velayutham successfully synthesized a bifunctional electrocatalyst, Fe‐MOF@MoS_2_‐6 h, embedded in a sulfur‐doped carbon matrix using a two‐step solvothermal method.^[^ ^147^ ^]^ The catalyst was prepared by growing an iron metal‐organic framework (Fe‐MOF) on a nickel foam substrate, followed by the in‐situ deposition of molybdenum disulfide on its surface, which resulted in outstanding OER electrocatalytic performance, attributed to its innovative surface‐oriented architecture. The LSV analysis demonstrated that the Fe‐MOF@MoS_2_‐6 h catalyst achieved a low overpotential of approximately 187 mV at 10 mA cm^−2^ and 251 mV at 100 mA cm^−2^, outperforming Fe‐MOF and other Fe‐MOF@MoS_2_ variants (Figure 13d,e). Moreover, the catalyst exhibited a small Tafel slope of 61.0 mV dec^−1^, indicating its superior OER kinetics compared to commercial IrO_2_/NF and other tested electrocatalysts (Figure 13f). The excellent catalytic properties of the Fe‐MOF@MoS_2_‐6 h catalyst are largely attributed to the enhanced electron and ion transport facilitated by the heterostructured nanorods and nanoflakes formed via the surface‐oriented growth of MoS_2_ on the Fe‐MOF. The nanoconfined environment within the sulfur‐doped carbon matrix is pivotal in suppressing nanoparticle agglomeration, thereby preserving structural integrity and optimizing electron transfer kinetics. Electrochemical impedance spectroscopy demonstrated that the Fe‐MOF@MoS_2_‐6 h catalyst exhibited the lowest charge transfer resistance among tested samples, confirming superior conductivity and rapid electron transfer capabilities. This confined architecture not only enhances catalytic activity but also ensures exceptional catalyst stability by maintaining structural robustness during operation.

The integration of 3D composite structures has been recognized as a highly effective strategy for enhancing catalytic performance.^[^ ^158^ ^]^ By combining different dimensional components, such as 0D nanoparticles and 1D fibers, researchers can engineer distinct structural configurations that maximize the exposure of active sites and improve the flow of reactants.^[^ ^159^ ^]^ Zhao et al. successfully synthesized a bimetallic iron‐cobalt sulfide catalyst (Fe_.92_Co_.08_S@SC/SCWF) embedded in a sulfur‐doped carbon matrix and anchored to sulfur‐doped carbonized wood fiber through synchronous carbonization and sulfidation.^[^ ^158^ ^]^ During the synthesis, a metal‐organic framework (MOF‐74) was used as a precursor and the Fe_.92_Co_.08_S nanoparticles were formed by reacting the metal ions within MOF‐74 with sulfur vapor, resulting in a catalyst composed of 0D materials encapsulated in a 3D sulfur‐doped carbon matrix. The unique structural design not only enhances the catalyst's surface area and active site density but also improves the flow of electrolytes and the transport of reactants, thereby accelerating the electrochemical reaction rate. The structural effect of this design is critical in preventing the agglomeration of Fe_.92_Co_.08_S nanoparticles, ensuring a uniform distribution and maximizing the exposure of active sites. The catalytic activity of Fe_.92_Co_.08_S@SC/SCWF was evaluated using a three‐electrode setup in 1.0 M aq. KOH. The LSV results revealed that the catalyst achieved current densities of 50 and 100 mA cm^−2^ at low overpotentials of 238 and 252 mV, respectively, outperforming other variants like FeS@SC/SCWF and Co_9_S_8_@SC/SCWF (Figure 13g). The catalyst also demonstrated excellent OER kinetics with a Tafel slope of 35.89 mV dec^−1^, significantly lower than that of commercial RuO_2_ and other comparison catalysts, indicating rapid OER reaction kinetics (Figure 13h). Moreover, EIS showed that Fe_.92_Co_.08_S@SC/SCWF had the lowest charge transfer resistance (0.35 Ω), further confirming its good charge transfer properties (Figure 13i). The remarkable catalytic properties of the Fe_.92_Co_.08_S@SC/SCWF catalyst are primarily attributed to the synergistic catalytic effect between the bimetallic Fe and Co components, as well as the optimization of the catalyst's microstructure within the sulfur‐doped carbon matrix. The successful synthesis of this catalyst demonstrates the advantages of 3D composite structures in electrocatalysis, where encapsulating 0D nanoparticles onto 1D fibers within a 3D framework significantly improves both stability and efficiency by optimizing active site distribution and conductivity.

In the pursuit of efficient, stable, and cost‐effective electrocatalysts to advance sustainable energy conversion technologies, Ren et al. developed a novel perovskite@zeolite composite (denoted as MAPbBr_3_@AlPO‐5), designed to enhance OER performance through unique structural engineering. The MAPbBr_3_@AlPO‐5 catalyst exhibited excellent performance, with an overpotential of 233 mV at 10 mA cm^−2^, a Tafel slope of 45.06 mV dec^−1^, and stability for 30 h.^[^ ^160^ ^]^ These improvements are attributed to dynamic interfacial engineering and synergistic effects within confined nanostructures. During OER, the composite restructures to form an edge‐sharing α‐PbO_2_ active layer, optimizing oxygen intermediate adsorption through charge transfer at the perovskite/α‐PbO_2_ interface. Perovskite nanocrystals confined within zeolite composite pores also improve water stability. In‐situ Raman verifies the reconstructed α‐PbO_2_ and retained zeolites while *ex‐situ* XPS analysis reveals that strong interfacial interactions between MAPbBr_3_ and the zeolite composite matrix promote the retention of the perovskite within subsurface layers, thereby contributing to its exceptional long‐term stability under water oxidation conditions. The DFT analyses along with electrochemical characterizations indicate that soft‐lattice halide perovskites facilitate lattice oxygen activation at the MAPbBr_3_/AlPO‐5 interface, promoting the lattice oxygen‐mediated mechanism driven OER pathways.

The ability of 3D confined structures to improve OER performance is exemplified by the Ru/TiO_x_ catalyst, where structural confinement not only stabilizes active sites but also enhances charge transfer efficiency and long‐term catalytic stability. This concept of 3D confinement is further demonstrated in the development of other electrocatalysts, such as Jang et al. developed a unique electrocatalyst, termed PCO‐nHI, composed of cobalt phosphide and cobalt oxide nanocubes that form subnanometer heterojunction interfaces at a 10 nm scale.^[^ ^161^ ^]^ In the preparation process, 0D cobalt phosphide and cobalt oxide nanocubes were uniformly dispersed on a 3D nickel foam substrate using a spray‐coating technique, followed by phosphidation, resulting in a catalyst composed of 0D materials on a 3D substrate. In an alkaline environment (1 M aq. KOH solution), the PCO‐nHI catalyst demonstrated outstanding OER performance, with an overpotential of 396.4 mV at a current density of 100 mA cm^−2^, indicating good catalytic activity. Additionally, the catalyst exhibited excellent stability, with an increase in overpotential of only 53 mV after 100 h of continuous testing at a current density of 100 mA cm^−2^. The DFT results further confirm that the subnanometer heterointerfaces effectively prevent the aggregation of nanocubes, thus maintaining the stability of the catalyst and improving both mass and charge transfer efficiency during the OER process. The superior performance arises from subnanometer heterojunction effects between cobalt phosphide and cobalt oxide, coupled with confined space effects. The heterojunction modulates oxygen intermediate adsorption/desorption, while confinement suppresses nanoparticle agglomeration‐enhancing electrochemical stability and reaction efficiency.

The use of 3D confined structures in electrocatalysts significantly enhances performance in the OER. By integrating nanoparticles and fibers within 3D frameworks, researchers can optimize active site exposure, improve electron and ion transport, and prevent nanoparticle aggregation. This confinement effect enhances the adsorption of oxygen intermediates and accelerates proton transfer, leading to faster reaction kinetics. DFT calculations highlight that the confined environment improves the electronic properties of metal sites, stabilizes catalysts, and maintains high catalytic efficiency over time. Additionally, 3D composite structures, such as carbon‐based frameworks and MOFs, provide both structural stability and conductivity, further boosting long‐term OER performance. These advances show that confined 3D nanostructures offer an effective strategy for improving the efficiency and durability of OER electrocatalysts.

In summary, confined effects play a crucial role in enhancing OER catalysts for water splitting. Utilizing 1D, 2D, and 3D confined spaces significantly improves catalytic efficiency by optimizing electron transfer, active site exposure, and reaction kinetics. In 1D systems, materials like CNTs improve stability and electron mobility, while 2D materials such as MXenes regulate interlayer spacing to enhance catalytic activity. In 3D systems, composites like MOFs offer larger surface areas, prevent aggregation, and enhance stability. DFT calculations provide valuable insights into how confined environments alter electronic structures, improve reactant adsorption, and reduce energy barriers.^[^ ^48^ ^]^ DFT simulations reveal that 1D and 2D confined spaces optimize metal–support interactions, while 3D systems stabilize active sites and prevent aggregation, further boosting OER performance. Despite these advancements, challenges remain in scaling and stabilizing catalysts for industrial applications. Future research should refine DFT models and focus on optimizing confined structures to meet the demands of large‐scale OER catalysis.^[^ ^116^ ^]^

## 6 Bifunctional Electrocatalysts for Overall Water Splitting in Confined Space

Bifunctional electrocatalysts for overall water splitting possess both highly efficient HER and OER activities, enabling a high current density at low operational potential while demonstrating robust stability and minimized maintenance expenditures. Compared to monofunctional catalysts, rational design of such integrated catalysts reduces system complexity and capital costs. Precious metal‐based hybrids demonstrate benchmark activity‐stability synergy, but their prohibitively high material costs constrain scalable deployment. The electrocatalytic performance of transition metal‐based catalysts such as NiFeMo alloy has been improved, but their inherent electrocatalytic activity and acid‐stability within low‐pH solutions remain suboptimal. The following section provides an in‐depth exploration of recent confined electrocatalysts for overall water splitting. A detailed overview of these catalysts and their properties is presented in Table 3, highlighting their performance characteristics, challenges, and potential for practical applications.

**Table 3:** Properties of 1D‐3D confined electrocatalysts for overall water splitting

Electrodes	Dimension	Cell voltage at 10 mA cm^−2^ (V)	Electrolyte	Stability (h)	Ref.
D‐CoNiO_x_‐P‐NFs (+/−)	1D	1.52	1 M aq. KOH	20	[170]
NFC@CNSs‐700 (+/−)	2D	1.59	1 M aq. KOH	24	[174]
NiFe@MoS_2_ (+/−)	2D	1.493	0.5 M aq. H_2_SO_4_	100	[111]
NCNT‐NP@NF (+/−)	3D	1.54	1 M aq. KOH	150	[179]
S‐NiFe_2_O_4_/NF (+/−)	3D	1.63	1 M aq. KOH	24	[181]
CoP@NPC (+/−)	3D	1.72	1 M aq. KOH	24	[182]
SC‐Cu_SA_‐NC (+/−)	3D	1.58	1 M aq. KOH	15	[183]
CoNiPO_x_@V_3%_‐Co_4_N/NF (+/−)	3D	1.52	1 M aq. KOH	50	[184]

### 6.1 Electrocatalysis in 1D Channels

Leveraging renewable energy for electrocatalytic water splitting is considered a promising approach for sustainable hydrogen production. Unlike the traditional method of developing separate electrocatalysts for the two half‐reactions of water splitting, namely the HER and the OER, increasing attention is being given to the design and development of bifunctional electrocatalysts. These catalysts are capable of efficiently promoting both HER and OER simultaneously.

Recent progress in developing cost‐effective electrocatalysts for water splitting has focused on materials such as transition metal oxides, hydroxides, and nitrides for OER, while HER relies on compounds like metal chalcogenides, carbides, and phosphides.^[^ ^164^, ^165^, ^166^, ^167^, ^168^ ^]^ However, few catalysts effectively facilitate both reactions across various pH conditions. Nitrogen‐doped carbon‐encapsulated iron‐group alloys are promising for water splitting, as their strong metal–carbon interaction enhances electronic coupling and enriches active sites. Alloy composition modifications fine‐tune adsorption energies, while single‐crystalline structures improve conductivity and mechanical stability, boosting efficiency and durability in water‐splitting. Zeng et al. synthesized a bifunctional catalyst composed of single‐crystalline cobalt–iron (CoFe) alloy nanoparticles encapsulated in nitrogen‐doped CNTs (CoFe@N‐C) via a straightforward heat‐treatment method.^[^ ^169^ ^]^ This catalyst features a core‐shell structure where 0D CoFe nanoparticles are enclosed within 1D nitrogen‐doped CNTs. The CoFe@N‐C catalyst demonstrated remarkable electrocatalytic performance in 1 M aq. KOH solution, with an overpotential of 292 mV for the OER at a current density of 10 mA cm^−2^ and a Tafel slope of 64 mV dec^−1^. For the HER, the overpotential was 110 mV under identical conditions. Furthermore, the catalyst exhibited exceptional stability, showing no significant change in overpotential after 1000 cycles of cyclic voltammetry and no observable decay in current density during a 20 h constant potential test. DFT calculations reveal that the carbon nanotube confinement modulates the charge distribution at the CoFe interface, leading to optimized binding energies for key oxygen intermediates. The encapsulation not only prevents particle agglomeration but also induces electronic delocalization between the alloy and the carbon support, which lowers the energy barrier for intermediate formation and accelerates the overall reaction kinetics. The superior performance of CoFe@N‐C can be attributed to the synergistic electronic coupling between the single‐crystalline CoFe alloy and the highly conductive nitrogen‐doped CNTs, along with the distinct benefits provided by the confined space structure, which improves both the stability and activity of the catalyst by precisely regulating the nanoscale environment around the active sites.

Embedding defective nanoparticles within a porous 1D structure presents a promising strategy for electrocatalysis, as it enhances defect site exposure and mass transport. However, achieving this in practice remains challenging. In a notable study, Hu et al. successfully fabricated a novel 1D porous nanofiber catalyst, D‐CoNiO_x_‐NFs, using a unique space‐confined strategy.^[^ ^170^ ^]^ The preparation involved embedding CoNi‐PBA nanoparticles into PAN nanofibers and subjecting them to air calcination, which resulted in a space‐confined effect, leading to the formation of numerous lattice defects and unsaturated metal sites. In their study, D‐CoNiO_x_‐P‐NFs, with a phosphorus content of approximately 12.9 wt%, exhibited significantly enhanced OER performance compared to CoNiO_x_‐P‐NFs. The water‐splitting cell assembled with D‐CoNiO_x_‐P‐NFs as both the anode and cathode electrocatalysts achieves a current density of 10 mA cm^−2^ at a low cell voltage of 1.52 V. This value is significantly lower than that required by the benchmark RuO_2_//Pt/C couple (1.61 V) and also outperforms other nonprecious metal‐based catalysts such as CuCoO‐NWs and V‐CoP@a‐CeO_2_. Moreover, the system demonstrates remarkable durability, maintaining a stable current density over 20 h of continuous operation without noticeable degradation. The enhanced catalytic activities of D‐CoNiO_x_‐P‐NFs are attributed to unsaturated metal sites and a porous 1D structure, which facilitate faster charge transfer and efficient mass transport‐improving performance in both OER and HER. Additionally, confined spaces from polyacrylonitrile contraction during calcination suppress nanoparticle aggregation, optimizing the local catalytic environment and enhancing electrocatalytic performance.

In the endeavor to improve the efficiency of electrocatalysts for HER and OER in electrochemical water splitting, Huo et al. developed a catalyst with platinum atoms confined within a MOF using pore space partitioning, forming a sandwich‐like structure. This design leverages the synergistic interaction between platinum and the MOF. The catalyst exhibited outstanding performance, with an overpotential of 2.5 mV for HER in acidic electrolyte and 265 mV for OER in alkaline electrolyte at 10 mA cm^−2^, a Tafel slope of 42.5 mV dec^−1^ for HER, and excellent stability over 12 h.^[^ ^171^ ^]^ These enhancements are attributed to atomic‐level confinement and unique electronic interactions within the MOF. XPS and XAS spectroscopy confirm the interactions between platinum atoms and the MOF, promoting H* adsorption and accelerating HER. The confined environment prevents platinum aggregation, ensuring stability. DFT calculations reveal that the higher electronegativity and electron density of the pyridinic nitrogen facilitate the formation of robust Pt─N coordination bonds, thereby enhancing the structural stability of the Pt array anchored between metal atoms and organic linkers

1D confined architectures enhance bifunctional electrocatalysis for water splitting due to their directional electron transport, high aspect ratios, and ability to confine active species. Embedding nanoparticles in carbon or polymer‐derived 1D frameworks exposes defect‐rich surfaces, prevents agglomeration, and improves conductivity. The confinement effect stabilizes intermediates, reshapes local coordination, and modulates HER and OER intermediate adsorption energies. DFT simulations show that 1D confinement promotes electronic delocalization, enriches d‐band centers, and reduces energy barriers for intermediate formation.^[^ ^172^ ^]^ Synergistic effects at metal–metal oxide, metal–carbon, or metal–hydroxide interfaces are amplified within 1D channels, especially with heteroatom doping or strain. However, 1D confined catalysts face scalability and uniformity issues, and their performance can decline at higher current densities due to mass transport limitations. The narrow confinement also restricts active site exposure, reducing efficiency for large‐scale use. These challenges highlight the need to explore 2D confined materials, which offer better tunability, accessibility for intermediates, and scalability. The next section will explore 2D confined architectures in bifunctional electrocatalysis for water splitting.

### 6.2 Electrocatalysis in 2D Layered Spaces

In recent years, 2D materials have gained attention for their exceptional electrocatalytic properties, driven by strong in‐plane bonding and weak interlayer interactions. These materials create confined spaces at interfaces, enabling novel chemical processes known as “chemistry under 2D confinement.” This environment enhances catalysis, surface adsorption, molecular growth, and electrochemical processes, transforming 2D materials into nanoreactors. The 2D layers regulate surface chemistry and catalytic activity, reducing molecular adsorption and promoting reactions. This ability to modulate molecular configurations and metal coordination improves electrocatalytic performance, particularly for water splitting.^[^ ^18^ ^]^ Transition metal‐based catalysts encapsulated in 2D materials benefit from improved catalytic activity, long‐term stability, and efficient bifunctional performance in both HER and OER, making them promising candidates for sustainable hydrogen production.^[^ ^173^ ^]^

**Figure 14:**
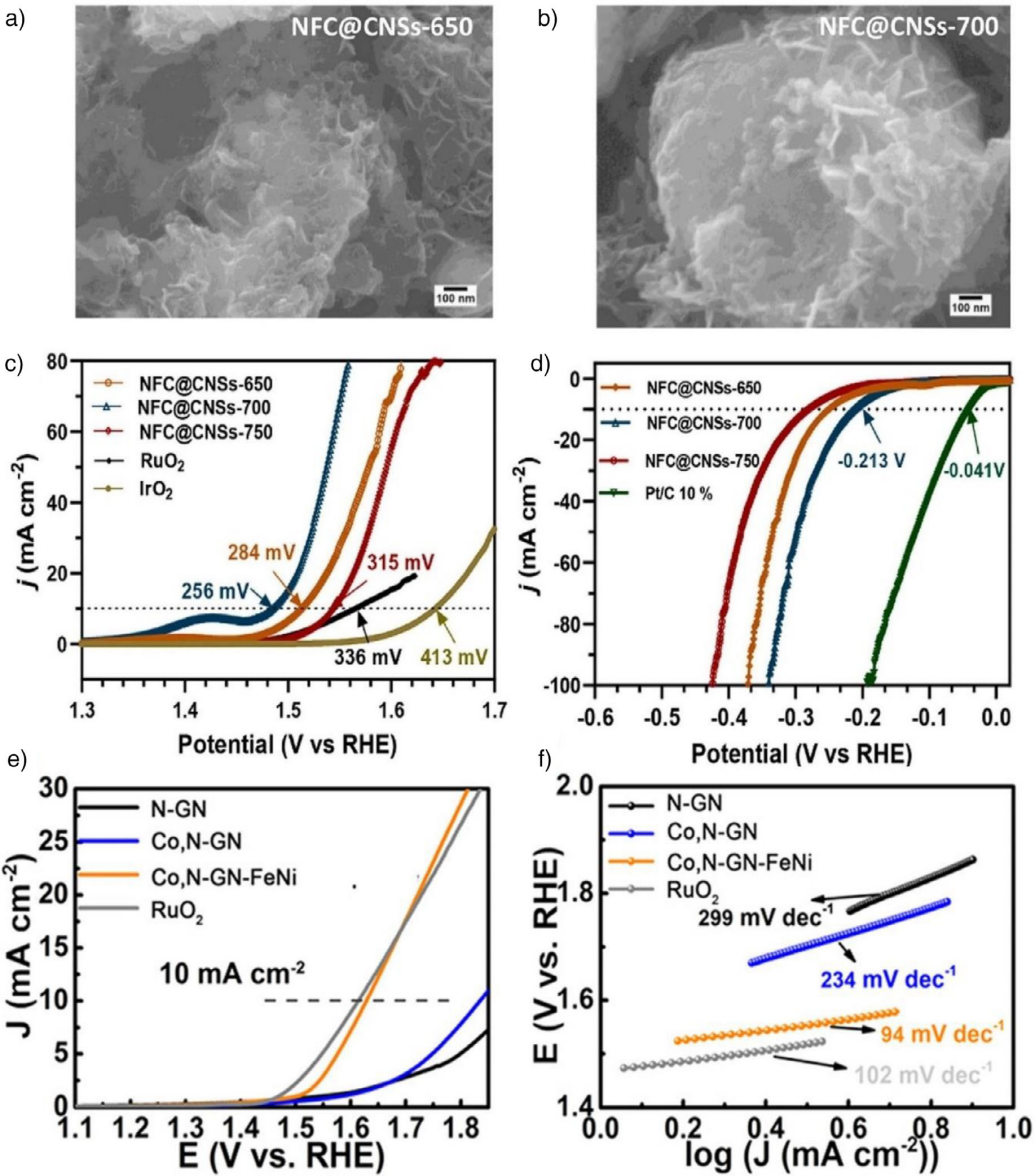
a) SEM image of NFC@CNSs‐650. b) SEM image of NFC@CNSs‐700. Reproduced from Ref.[174] Copyright 2021, with permission from American Elsevier. c) LSV curves showing the OER performance of the as‐prepared catalysts. d) LSV curves illustrating the HER activity of the catalysts. Reproduced from Ref.[174] Copyright 2021, with permission from Elsevier. e) LSV profiles of the samples for the OER and f) corresponding Tafel plots. Reproduced from Ref.[175] Copyright 2020, with permission from American Chemical Society.

The development of bifunctional electrocatalysts for simultaneous HER and OER has become central to efficient, sustainable hydrogen production. Transition metal‐based catalysts, being abundant and cost‐effective, offer promising alternatives to noble metals. When integrated into 2D materials, these catalysts utilize confined spaces to enhance both HER and OER activity, advancing hydrogen production technologies. Yaseen et al. synthesized a novel Ni–Fe–Co‐based nanoparticle catalyst (NFC@CNSs) encapsulated in 2D carbon nanosheets using a one‐step pyrolysis strategy.^[^ ^174^ ^]^ This innovative catalyst was engineered to overcome the performance limitations of previous materials by enhancing both HER and OER activities. Figure 14a,b shows the SEM images of NFC@CNSs‐650 and NFC@CNSs‐700, highlighting their morphological differences due to calcination temperatures, Figure 14c,d presents the LSV polarization curves of the as‐prepared catalysts for the HER and OER, showcasing their electrocatalytic performance. The NFC@CNSs‐700 catalyst demonstrated exceptional electrocatalytic activity in a 1.0 M aq. KOH solution, exhibiting low overpotentials (96.1 mV for HER and 240 mV for OER), small Tafel slopes (115.1 mV dec^−1^ for HER and 61.29 mV dec^−1^ for OER), and excellent long‐term stability, maintaining sustained water splitting for 150 h. These attributes resulted in the NFC@CNSs‐700 catalyst achieving outstanding overall water splitting performance, reaching a current density of 10 mA cm^−2^ at a cell voltage of only 1.54 V. The superior performance of the NFC@CNSs catalyst is attributed to the synergistic interaction between the trimetallic nanoparticles and the carbon nanosheets, which enhance electron transfer, provide abundant active sites, and prevent nanoparticle aggregation. DFT calculations support these findings, revealing that the spatial confinement within the carbon nanosheets induces electron redistribution among the Ni, Fe, and Co components, leading to a more favorable electronic environment at the catalyst surface. This modulated electronic structure reduces the energy barriers for OER intermediate adsorption and accelerates charge transfer kinetics. Additionally, the confined carbon matrix stabilizes the metal nanoparticles against oxidation and migration, ensuring both structural integrity and sustained catalytic activity. The confined space within the carbon nanosheets plays a crucial role in stabilizing the nanoparticles, thereby optimizing the catalytic environment and ensuring long‐term durability.

Building on the insights from NFC@CNSs, the development of confined growth and doping of transition metals within carbon hosts presents another promising avenue for improving bifunctional catalytic activities. Achieving both confined growth and doping of transition metals within carbon hosts is a highly promising approach to producing distinct bifunctional catalytic activities. However, this process remains challenging due to the difficulty of achieving synchronous nucleation and diffusion of metallic ions in a single synthesis step. Ding et al. developed a novel catalyst, Co,N‐codoped graphene‐confined FeNi nanoparticles (Co,N‐GN‐FeNi), synthesized through a simple pyrolysis process. This catalyst includes the high OER activity of FeNi nanoparticles.^[^ ^121^ ^]^ Figure 14e presents the LSV curves of the samples during the OER process, revealing their electrocatalytic capabilities. At a current density of 10 mA cm^−2^, the overpotential of Co,N‐GN‐FeNi was 1.63 V, outperforming Co,N‐GN and N‐GN and thus demonstrating better catalytic activity for the OER. Figure 14f presents the corresponding Tafel plots, where the 94 mV dec^−1^ Tafel slope of Co,N‐GN‐FeNi is significantly lower than those of Co,N‐GN (234 mV dec^−1^) and N‐GN (299 mV dec^−1^), indicating excellent OER kinetics. DFT calculations reveal that the spatial confinement of FeNi within the graphene matrix promotes strong metal–support interactions, which shift the d‐band center of the active metals closer to the Fermi level. This shift enhances the adsorption–desorption balance of oxygen intermediates and facilitates charge delocalization across the metal–carbon interface. The presence of Co and N dopants further adjusts the local electronic structure, creating energetically favorable sites for OER steps and suppressing surface oxidation or restructuring. The enhanced OER catalytic activity of Co,N‐GN‐FeNi can be ascribed to the additional coupling of uniformly distributed FeNi nanoparticles confined‐grown within the Co,N‐GN host. Moreover, the relatively low Tafel slope of Co,N‐GN compared to N‐GN highlights how Co doping also plays a significant role in improving the OER kinetics. In conclusion, the confined growth of FeNi nanoparticles within the graphene host and Co doping are crucially important to enhancing the catalyst's performance, as they effectively enhance electron transfer, stabilize active sites, and reduce energy barriers.

Although the Co,N‐GN‐FeNi catalyst demonstrates impressive performance in the OER, it is crucial to explore additional materials that exhibit enhanced bifunctional catalytic activity for both HER and OER. Jiang et al. developed an interlayer‐confined NiFe dual‐atom catalyst within MoS_2_, termed NiFe@MoS_2_.^[^ ^111^ ^]^ This catalyst was synthesized using laser molecular beam epitaxy followed by a self‐curving treatment, which confined Ni and Fe atoms within the interlayer space of 2D MoS_2_ nanoscrolls. The NiFe@MoS_2_ catalyst exhibited remarkable bifunctional electrocatalytic performance in acidic solutions. For the HER, it achieved an overpotential of 67 mV at a current density of −10 mA cm^−2^, with a Tafel slope of 58 mV dec^−1^. For the OER, it demonstrated an overpotential of 201 mV at 10 mA cm^−2^, with a Tafel slope of 86 mV dec^−1^. The catalyst also showed excellent stability, maintaining its performance for over 50 h of continuous testing. The outstanding performance is attributed to the confined interlayer space, which enhances the adsorption strength on the active metal centers and protects the dual atoms from aggregation and degradation, thereby improving catalytic activity and ensuring long‐term durability.

In the development of efficient and stable electrocatalysts for overall water splitting in acidic media, innovative strategies are crucial for enhancing performance and durability. Jiang et al. introduced a novel electrocatalyst: NiFe dual atoms confined within MoS_2_ nanoscrolls (NiFe@MoS_2_) to enhance water splitting efficiency and stability in acidic media. The catalyst showed exceptional performance, with an overpotential of 67 mV for the HER at −10 mA cm^−2^ (Tafel slope of 26.8 mV dec^−1^) and 201 mV for the OER at 10 mA cm^−2^ (Tafel slope of 48.3 mV dec^−1^), maintaining activity for over 100 h.^[^ ^111^ ^]^ DFT calculations revealed that the interlayer‐confined structure optimizes the adsorption strength of reaction intermediates, enhancing efficiency. The MoS_2_ nanoscrolls protect the NiFe dual atoms from corrosion while promoting synergistic interactions, ensuring stability. In a two‐electrode cell, the NiFe@MoS_2_ catalyst achieved a cell voltage of 1.493 V at 10 mA cm^−2^, outperforming the IrO_2_||Pt/C electrolyzer. The XAS and EELS, as well as DFT calculations, further elucidate the role of the interlayer‐confined structure in optimizing the adsorption strength of reaction intermediates and enhancing catalytic efficiency. This work highlights the role of dynamic interfacial engineering and dual‐site synergy in improving electrocatalytic performance for water splitting in acidic environments.

2D confined electrocatalytic systems enhance overall water splitting by improving activity, selectivity, and stability. Their planar structures provide high active site exposure, rapid charge transport, and controlled confinement of metal species. Embedding nanoparticles or atomic clusters in graphene, carbon nanosheets, or metal‐organic matrices prevents aggregation and ensures structural rigidity under harsh conditions. DFT calculations show that nanoscale confinement reshapes the electronic structure by modulating d‐band centers, charge redistribution, and orbital overlap with key intermediates.^[^ ^18^ ^]^ These modifications lower activation barriers for HER and OER, optimize adsorption–desorption energetics, and enhance metal–support interactions. Heteroatom doping and dual‐metal coordination in 2D layers create catalytically favorable sites while maintaining structural integrity. Together, these features make 2D confined architectures highly efficient and durable for bifunctional water splitting.^[^ ^173^ ^]^

### 6.3 Electrocatalysis in 3D Confined Spaces

**Figure 15:**
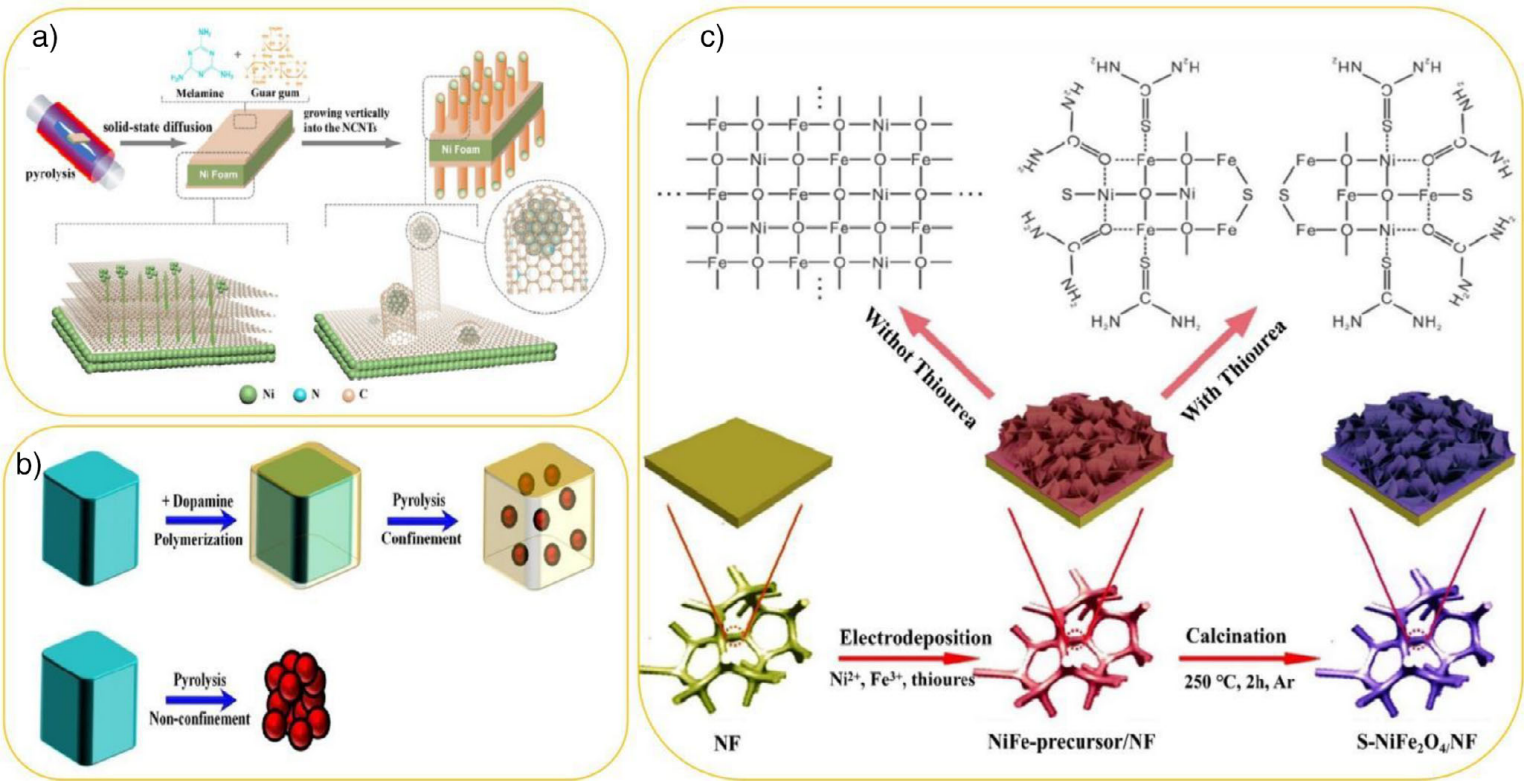
a) Schematic representation of the synthesis process for NCNT‐NP@NF. Reproduced from Ref.[179] Copyright 2020, with permission from Elsevier. b) Schematic illustrating the product morphology resulting from pyrolysis under spatially confined and nonconfined conditions. Reproduced from Ref.[180] Copyright 2018, with permission from Elsevier. c) Schematic depiction of the confined growth process for S‐NiFe_2_O_4_/NF, facilitated by thiourea. Reproduced from Ref.[181] Copyright 2017, with permission from Elsevier.

Unlike 1D and 2D nanoconfinement, 3D nanoconfinement offers less restriction on molecular size, allowing reactants and intermediates to participate more effectively in reactions. This is beneficial for bifunctional catalysts, enhancing both HER and OER performance. The broader spatial environment supports free diffusion, efficient charge transfer, and interaction with the catalyst surface, improving reaction kinetics and selectivity. These features make 3D nanoconfinement ideal for water splitting reactions requiring dual functionality in electrocatalysis. In the realm of electrocatalysis, nanocarbon materials,^[^ ^176^ ^]^ including CNTs,^[^ ^20^ ^]^ graphene, and aerogels, along with their corresponding heteroatom doping such as nitrogen, sulfur, boron, and phosphorus, are of considerable interest given their stability in harsh acid and alkali environments, adjustable molecular structures, and distinctive electronic characteristics.^[^ ^177^, ^178^ ^]^ Particularly, various heteroatom‐doped CNTs have been identified as potential substitutes for noble metals. Incorporating transition metals into heteroatom‐doped CNTs has become a well‐established strategy for enhancing the performance of electrocatalysts. Chen et al. successfully developed a novel self‐supported bifunctional electrocatalyst, Ni nanoparticles encapsulated within vertically aligned nitrogen‐doped CNTs (NCNT‐NP@NF), using a solid‐state diffusion approach (Figure 15a).^[^ ^179^ ^]^ This process involved spraying a guar gum and melamine solution onto nickel foam, followed by drying and high‐temperature pyrolysis, yielding a 3D catalyst with 1D nitrogen‐doped CNTs on a 3D nickel foam substrate. The NCNT‐NP@NF demonstrated remarkable electrocatalytic activity in 1.0 M aq. KOH solution, with low overpotentials of 96.1 mV for HER at 10 mA cm^−2^ and 240 mV for OER, and retained excellent stability for over 150 h during water splitting. The outstanding performance is attributed to the uniform distribution of Ni nanoparticles, electronic modulation by nitrogen doping, protective encapsulation by NCNTs, and the abundant channels offered by the vertically aligned NCNTs for electron and mass transport. DFT calculations provide mechanistic insights into this performance, showing that the encapsulation of Ni nanoparticles within nitrogen‐doped CNTs modifies the local electronic environment by inducing charge transfer from Ni to the surrounding carbon matrix. This interaction shifts the electronic states of surface Ni atoms, optimizing their binding strength with oxygen intermediates involved in OER. Moreover, the aligned CNT architecture enhances the orbital overlap between Ni d‐states and adsorbed species, thereby reducing the energy barriers associated with reaction steps. The confinement also acts as an energetic stabilizer, preventing surface reconstruction under oxidative potential and preserving the activity of the catalytic interface. These combined effects underscore the role of nanoscale confinement in tuning the catalytic landscape and ensuring sustained electrocatalytic performance.

In a similar approach, Zhang et al. synthesized a novel particle‐in‐box nanostructure, an N‐doped graphene layer coated with Fe–Ni alloy nanoparticles encapsulated within N‐doped carbon hollow nanoboxes, using a spatially confined pyrolysis process (Figure 15b).^[^ ^180^ ^]^ This 3D catalyst consists of a 2D N‐doped graphene layer coating on Fe–Ni alloy nanoparticles encapsulated within a 3D N‐doped carbon hollow nanobox. In an alkaline solution, the catalyst exhibits excellent electrocatalytic performance, with low overpotentials of 270 mV for the OER and 201 mV for HER at 10 mA cm^−2^, small Tafel slopes of 63.9 mV dec^−1^ for OER and 50 mV dec^−1^ for HER, high current density, and excellent long‐term stability with only a slight increase in potential after 10 h of testing at 10 mA cm^−2^. Such performance is considered to be very good, and is mainly due to the advantageous particle‐in‐box nanostructure, which offers a confined and effective reaction environment, and the synergistic effects resulting from the conductive, hollow, and porous carbon shell and the small size of the Fe‐Ni alloy nanoparticle core and its N‐doped graphene layer shell. DFT calculations further reveal that the spatial confinement within the hollow nanoboxes induces local charge redistribution at the Fe–Ni/carbon interface, enhancing the electronic coupling between metal d‐orbitals and the N‐doped carbon matrix. This confinement‐driven interfacial interaction results in a downward shift in the projected density of states of surface Fe and Ni atoms, which reduces the binding energy of key intermediates and facilitates their dynamic conversion during the OER. Moreover, the curvature and internal strain generated within the hollow carbon shells modulate the local electronic potential, stabilizing reaction pathways and suppressing surface oxidation. Additionally, N‐doping enhances the electronic properties of the carbon materials, increasing the number of active sites and thus boosting catalytic activity.

NiFe‐based compounds, abundant, cost‐effective, and with unique properties, show promise as alternatives to noble metal catalysts for HER and OER. To maximize their catalytic potential in alkaline and neutral media, well‐structured NiFe‐based nanomaterials are essential for optimizing performance in both reactions. Liu et al. synthesized sulfur‐incorporated nickel ferrite (S‐NiFe_2_O_4_) nanosheets composed of ultrasmall nanoparticles (∼2 nm) on nickel foam (NF) using a thiourea‐assisted electrodeposition method followed by low‐temperature calcination (Figure 15c).^[^ ^181^ ^]^ This process resulted in a 3D hierarchical structure with interconnected nanosheets built from ultrasmall nanoparticles, providing abundant active sites and high electrical conductivity. The S‐NiFe_2_O_4_/NF catalyst demonstrated excellent bifunctional catalytic performance for water splitting in both alkaline (1 M aq. KOH) and neutral (1 M aq. PBS) solutions, achieving a current density of 10 mA cm^−2^ at overpotentials of 138 mV for HER and 267 mV for OER in alkaline solution, and 197 mV for HER and 494 mV for OER in neutral solution. S‐NiFe_2_O_4_/NF showed excellent long‐term durability. Its performance stems from ultrasmall nanoparticles (large surface area, abundant active sites) and sulfur doping (enhanced electronic properties/conductivity). DFT calculations provide further insight, demonstrating that sulfur incorporation alters the local charge density around Ni and Fe centers, introducing asymmetry into the surface electronic structure. This asymmetry lowers the energetic barrier for adsorption and transformation of key intermediates during the OER process. Moreover, the nanoscale confinement of the Fe–O and Ni–O coordination environments within the thin nanosheets modifies the electronic distribution and orbital overlap with reactant species, thereby accelerating surface redox kinetics. The confined domains also maintain a higher density of active high‐spin Fe^3+^ and Ni^2+^ sites under electrochemical conditions, preserving catalytic integrity during prolonged operation. The confined growth strategy‐controlled particle size/distribution, prevented aggregation, and optimized the catalytic environment, improving structural stability and water splitting performance.

Efficient and stable bifunctional electrocatalysts are essential for water splitting, which is crucial for sustainable hydrogen production. Although traditional Pt and RuO_2_ catalysts are effective, their high cost and limited availability have driven the search for more affordable alternatives. Sun et al. developed a 3D nanostructured catalyst comprising nanosized transition metal phosphides encapsulated in nitrogen‐phosphorus codoped carbon (TMP@NPC) using adenosine triphosphate as the phosphorus source.^[^ ^182^ ^]^ The synthesis involved mixing transition metal salts with ATP and melamine, followed by heating at 800 °C in an argon/hydrogen atmosphere. This process produced TMP nanoparticles uniformly encapsulated in a codoped carbon matrix, forming a core‐shell structure that enhances stability and performance. Among these, CoP@NPC showed the best bifunctional electrocatalytic performance in 1 M aq. KOH, with an overpotential of 184.35 mV for HER and 303 mV for OER at 10 mA cm^−2^. The catalyst also demonstrated excellent stability over 50 h of continuous operation. The high performance is attributed to the nanosized CoP particles and the N, P codoped carbon shell, which enhances conductivity, provides abundant active sites, and prevents nanoparticle aggregation. The confined space within the carbon shell stabilizes the nanoparticles and optimizes the local catalytic environment, improving overall efficiency.

**Figure 16:**
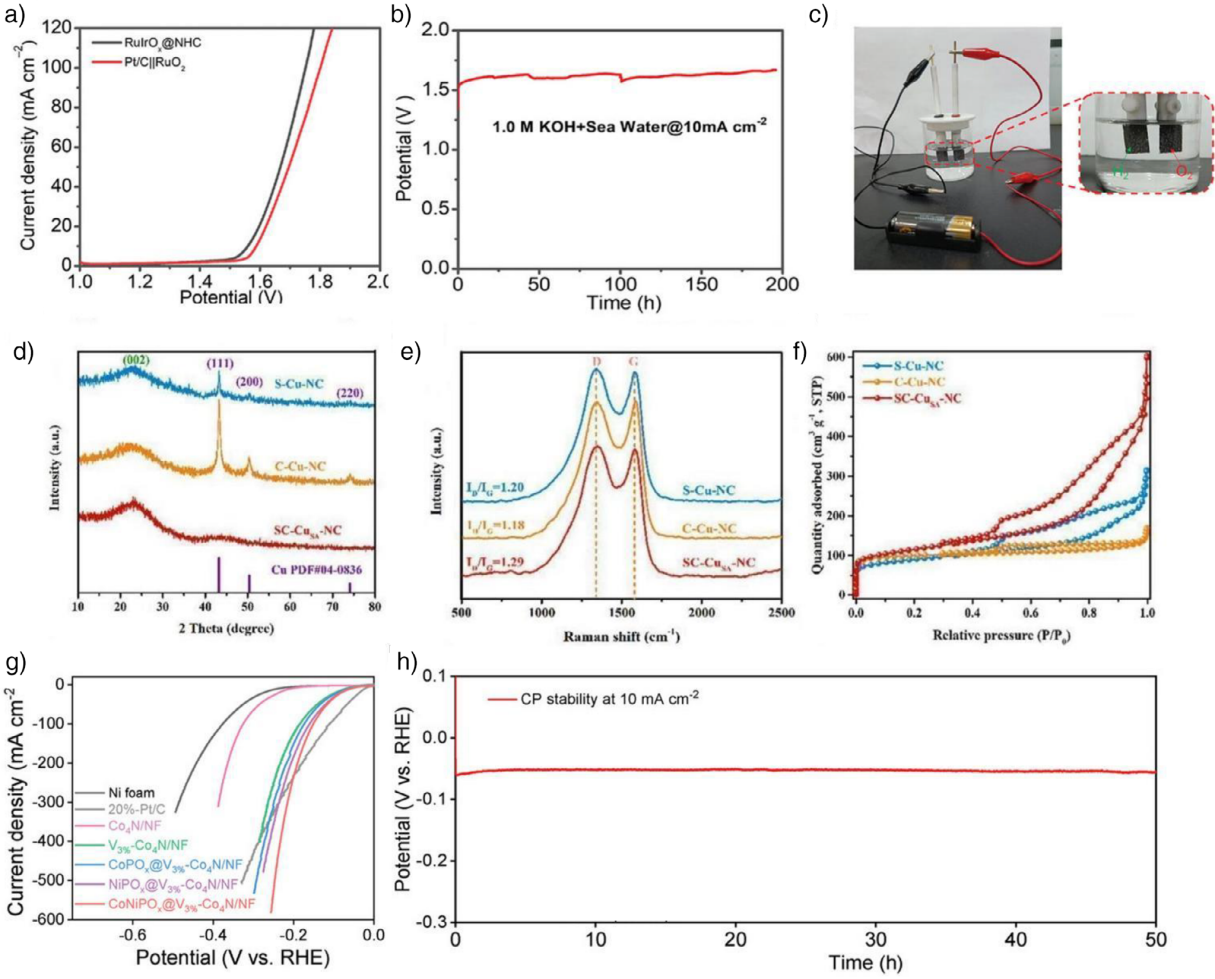
a) Polarization curves comparing the overall water splitting performance of the RuIrO_x_@NHC // RuIrO_x_@NHC system with that of the commercial Pt/C // RuO_2_ couple. b) Cell voltage profile of the RuIrO_x_@NHC // RuIrO_x_@NHC configuration recorded over 200 h at a constant current density of 10 mA cm^−2^. c) Photograph showing overall water splitting powered by a standard 1.5 V battery using RuIrO_x_@NHC electrodes. Reproduced from Ref. [6] Copyright 2023, with permission from John Wiley and Sons. d) XRD pattern, e) Raman spectra, f) N_2_ adsorption–desorption isotherm. Reproduced from Ref.[183] Copyright 2023, with permission from Elsevier. g) LSV curves of electrolyzers configured with CoNiPO_x_@V_3%_‐Co_4_N/NF (+/−), NiPO_x_@V_3%_‐Co_4_N/NF (+/−), CoPO_x_@V_3%_‐Co_4_N/NF (+/−), V_3%_‐Co_4_N/NF (+/−), Co_4_N/NF (+/−), commercial RuO_2_/NF (+)  // Pt‐C/NF (−), and bare Ni foam electrodes. h) Durability assessment of CoNiPO_x_@V_3%_‐Co_4_N/NF(+/−) under a constant applied voltage of 1.7 V over extended operation. Reproduced from Ref.[184] Copyright 2022, with permission from John Wiley and Sons.

Although Pt‐based HER catalysts are highly effective, their scarcity and high cost limit their widespread use. Ru‐based electrocatalysts, offering comparable HER activity at a lower cost, have emerged as a promising alternative. In this context, Lu et al. developed a bifunctional catalyst composed of 3 nm RuIrO_x_ nanocrystals anchored onto nitrogen‐doped hollow carbon (NHC), referred to as RuIrO_x_@NHC.^[^ ^6^ ^]^ This catalyst was synthesized using a carbonization strategy followed by HF etching, resulting in strong anchoring between the RuIrO_x_ nanocrystals and the carbon skeleton, effectively restricting nanocrystal growth during high‐temperature processing. As shown in Figure 16a,b, the RuIrO_x_@NHC catalyst exhibits excellent overall water splitting performance in alkaline seawater electrolyte, requiring only a low cell voltage of 1.54 V to reach a current density of 10 mA cm^−2^, and maintaining stable activity for up to 200 h. Notably, it also demonstrates remarkable durability, operating continuously for 100 h at 0.5 A cm^−2^ and 40 h at 1 A cm^−2^ in 1 M KOH mixed with seawater. As illustrated in Figure 16c, the catalyst can drive overall water splitting using a 1.5 V AA (alkaline) battery, with visible generation of H_2_ and O_2_ bubbles at the cathode and anode, respectively. These gases were quantitatively collected via a drainage gas collection method, revealing Faradaic efficiencies exceeding 98.5% in both acidic and alkaline media. The molar ratio of hydrogen to oxygen approached the theoretical value of 2:1, further confirming the exceptional application potential of RuIrO_x_@NHC for overall water electrolysis. The RuIrO_x_@NHC catalyst features a high surface area, and a multilayered porous carbon framework with well‐distributed nanocrystals, providing abundant active sites for catalytic reactions. Additionally, RuIrO_x_@NHC maintained excellent stability over 2000 cycles and prolonged electrolysis tests. This remarkable performance is attributed to the strong interfacial bonding between the RuIrO_x_ nanocrystals and the nitrogen‐doped hollow carbon skeleton, which enhances electron transfer and stabilizes active sites. The confined space within the nitrogen‐doped hollow carbon restricts nanocrystal growth, increasing the number of active sites and enhancing overall catalytic activity, while also preventing agglomeration and ensuring consistent performance during extended catalysis.

Single‐atom catalysts are gaining significant attention due to their high atomic utilization, which maximizes the efficiency of each metal atom involved in catalysis. Zhang et al. developed SC‐Cu_SA_‐NC, an N‐doped porous carbon catalyst with single Cu atoms, via flash nanocomplexation and a double‐network gel strategy.^[^ ^183^ ^]^ This method ensures the uniform dispersion and spatial isolation of Cu atoms within the carbon network. The catalyst was prepared by forming a sodium alginate/carboxymethyl cellulose‐Cu double‐network hydrogel through FNC, followed by freeze‐drying and pyrolysis at 800 °C. The resulting 3D interconnected porous carbon network confines Cu single atoms within a nitrogen‐doped carbon matrix, forming highly active Cu‐N_4_ coordination sites. Figure 16d presents the XRD pattern, highlighting the crystallographic structure of the materials with distinctive peaks that correspond to the various phases present. Figure 16e shows the Raman spectra, where the peaks reveal the coexistence of graphitic carbon and disordered carbon structures within the material. Lastly, Figure 16f depicts the N_2_ adsorption–desorption isotherms, indicating the material's porosity. The presence of both mesopores and micropores contributes to the high surface area and improved catalytic performance. SC‐Cu_SA_‐NC demonstrated outstanding bifunctional electrocatalytic performance in 1 M aq. KOH solution, achieving overpotentials of 124 mV for HER and 314 mV for OER at the current density of 10 mA cm^−2^. A two‐electrode water splitting system employing SC‐Cu_SA_‐NC as both the anode and cathode delivers a low voltage of 1.58 V at 10 mA cm^−2^ and exhibits the stability of constant voltage over 15 h. The good performance is attributed to the unique 3D carbon network, which enhances mass transport, provides abundant active sites, and stabilizes the Cu atoms. The confined space within the double‐network gel during FNC plays a critical role in preventing Cu atom aggregation, ensuring uniform dispersion, and maintaining catalyst stability during pyrolysis, thereby optimizing the catalytic environment and enhancing the overall electrochemical performance.

Integrating ultrathin amorphous transition metal phosphate nanosheets onto electron‐tuned crystalline core‐shell structures enhances HER activity by lowering hydrogen adsorption energy. This improves HER efficiency and addresses slow kinetics in both OER and HER in alkaline media. The optimized structure balances electronic properties, promoting faster electron transfer and improving overall electrocatalytic performance. For example, Singh et al. developed a novel 3D heterostructured electrocatalyst comprising an amorphous CoNi phosphate (CoNiPO_x_) shell over a crystalline core of vanadium‐doped cobalt nitride nanowires (V_3%_‐Co_4_N), referred to as CoNiPO_x_@V_3%_‐Co_4_N/NF.^[^ ^184^ ^]^ Synthesized via hydrothermal, nitridation, and electrodeposition: V‐doped Co‐CHH nanowires grew on Ni foam, formed V‐Co_4_N via ammonia nitridation, and coated with CoNiPO_x_ nanosheets. The CoNiPO_x_@V_3%_‐Co_4_N/NF (+/−) has a 3D mesoporous structure (amorphous shell/crystalline core), offering abundant active sites. In 1 M KOH, it showed 53 mV (HER) and 270 mV (OER) overpotentials at 10 mA cm^−2^, with high long‐term stability. As shown in Figure 16g, under the conditions of a scan rate of 2 mV s^−1^ and a potential window of 1–2 V, the CoNiPO_x_@V_3%_‐Co_4_N/NF (+/−) electrolyzer exhibits optimal performance: it has the maximum current density and the lowest cell potential at all current densities. Specifically, this electrolyzer only requires an extremely low cell potential of 1.52 V to reach a current density of 10 mA cm^−2^. In addition to its excellent catalytic activity, the long‐term stability of the electrolyzer is also a key indicator of its practical application value. As shown in Figure 16h, the long‐term durability of the CoNiPO_x_@V_3%_‐Co_4_N/NF (+/−) alkaline electrolyzer was investigated using the chronoamperometric (CA) technique: when continuously operated at an applied potential of 1.7 V for 50 h, its current density gradually increased during the initial h of operation, then stabilized over time, and only showed minimal deterioration even after 50 h. The superior performance of CoNiPO_x_@V_3%_‐Co_4_N/NF (+/−) is attributed to the synergy between the amorphous CoNiPO_x_ shell and the crystalline V_3%_‐Co_4_N core, which lowers charge‐transfer resistance, increases the electrochemically active surface area, and optimizes the electronic structure. The confined space within the amorphous shell and crystalline core plays a crucial role in improving the catalyst's performance by ensuring the uniform distribution of active sites and facilitating efficient electron and ion transport, leading to enhanced catalytic activity and long‐term stability.

3D‐confined architectures enhance bifunctional electrocatalysis in HER and OER by providing interconnected porous networks, hierarchical channels, and restricted domains. These features increase active site exposure, efficient charge/mass transport, and improve catalyst–electrolyte interaction. Encapsulating active species in carbon matrices, oxide shells, or doped frameworks prevents aggregation and ensures stability. DFT calculations show that 3D confinement redistributes charge, modulates coordination environments, and enhances orbital interactions, optimizing H* and OH*/OOH* adsorption energies.^[^ ^172^ ^]^ Additionally, Strain effects and defect states from spatial confinement lower energy barriers and accelerate HER/OER kinetics. These structural and electronic advantages make 3D‐confined systems a promising strategy for developing high‐performance, durable, and cost‐efficient electrocatalysts for water splitting.

## 7 Summary and Outlook

### 7.1 Confined Catalyst Architectures: Merits and Limitations

1D, 2D, and 3D nanostructures each offer distinct advantages in catalysis but also face specific challenges. 1D nanostructures provide high specific surface areas with abundant catalytically active sites, promoting efficient reactant adsorption and surface reactions. Their anisotropic electron transport allows for rapid charge transfer kinetics, and their exceptional mechanical robustness helps maintain structural integrity during electrochemical cycling. However, the preparation of 1D catalysts is complex and costly, and although these structures offer numerous active sites, many may be encapsulated internally, limiting their exposure to reactants. This restricts their catalytic efficiency, necessitating further optimization of their composition for performance enhancement in various electrolytes.

2D materials, such as graphene derivatives, offer high active site exposure and superior electronic conductivity, facilitating efficient interfacial charge migration. However, they tend to stack, reducing the availability of active sites, and some materials suffer from poor stability, with potential structural changes during prolonged electrochemical reactions. Strategies to prevent stacking and enhance stability are crucial for improving their long‐term performance.

3D frameworks incorporate high‐density active sites within interconnected pore networks, which enhance reactant diffusion and reaction accessibility. These hierarchical structures offer improved mechanical stability and percolative electron conduction pathways, which maintain performance stability during electrocatalytic processes. However, the preparation of 3D catalysts is also complex and costly, requiring the development of simpler synthesis methods to optimize their structure and stability.

In summary, while 1D, 2D, and 3D nanostructures each offer unique advantages for catalysis, addressing challenges in preparation, stability, and optimization of active site exposure is critical for their large‐scale application and efficiency in catalysis.

### 7.2 Confined Catalysts in the Progress and Challenges of Large‐Scale Hydrogen Generation

The application of confined catalysts in industrial hydrogen production is still in the early stages of research and development, with no large‐scale industrial application yet realized. Although the confinement effect has demonstrated significant advantages in catalyst design, including regulating the electronic structure of catalysts, enhancing reaction activity and selectivity, and improving catalyst stability and durability, applying it in industrial hydrogen production faces multiple challenges. In laboratory‐scale studies, confined catalysts have shown promising performance. For instance, in technologies such as proton exchange membrane fuel cells (PEMFCs) and solid oxide electrolysis cells (SOECs), confined catalysts can significantly improve hydrogen production efficiency and selectivity.^[^ ^185^ ^]^ However, the complexity and high cost of these technologies have restricted their large‐scale industrial application. Additionally, the long‐term stability of confined catalysts under industrial operating conditions has not been fully verified, and this remains a critical obstacle to their large‐scale deployment.

At present, the application of confined catalysts in industrial hydrogen production is primarily concentrated in laboratory settings and small‐scale demonstration projects. For example, some research teams have achieved efficient hydrogen generation by encapsulating catalysts within nanoporous materials, such as MOFs and CNTs.^[^ ^186^, ^187^ ^]^ However, these technologies face the following challenges in large‐scale applications:

I. Cost issues: The preparation process of confined catalysts is complex, involving high‐precision synthesis of nanomaterials and encapsulation techniques, resulting in high costs. For instance, the synthesis of MOFs requires specific organic ligands and metal ions, whose costs are significantly higher than those of traditional catalysts. Additionally, the encapsulation of 1D channels demands precise control over their internal space, further increasing preparation costs. This makes the economic viability of confined catalysts in industrial applications questionable. Recent reports from international institutions emphasize that catalysts must reduce the production cost per kilogram of hydrogen, and current high‐cost preparation processes fail to meet this critical criterion.

II. Stability issues: Although confined catalysts exhibit good stability in laboratory environments, their activity may decline, or they may even deactivate under industrial conditions due to exposure to high temperatures, high pressures, and corrosive gases. At high temperatures, the structural integrity of catalyst encapsulating materials may be compromised, negatively impacting their catalytic performance. This highlights the need for further validation of the long‐term stability of confined catalysts under industrial conditions. As noted in international reports, industry standards call for catalysts to operate continuously for thousands of h, and current confined catalyst systems have not yet reliably achieved this durability benchmark.

III. Scalability issues: The preparation of confined catalysts typically requires precise control over nanostructures, which is difficult to achieve in large‐scale production. For example, the encapsulation of CNTs necessitates accurate regulation of their internal space to ensure uniform distribution of catalysts and efficient reactions.^[^ ^186^ ^]^ Moreover, controlling uniformity and consistency during large‐scale production remains a technical challenge, which is closely linked to the overall cost and practicality of these catalysts in industrial settings.

IV. Technical maturity issues: The application of confined catalysts in industrial hydrogen production is still in the technical verification stage, lacking long‐term operation data to meet the reliability requirements of industrial production. Current research is mostly focused on laboratory scales, with insufficient data from practical applications in industrial environments. This highlights the need to further enhance the technical maturity of confined catalysts for industrial hydrogen production, which entails integrating material science with engineering, policy, and economics. Ultimately, when evaluating new catalysts like confined systems, performance in the lab is only part of the equation. Equal consideration must be given to cost, service life, supply chain stability, recyclability, and energy consumption during production. Even a catalyst with exceptional lab performance would be of little use to hydrogen energy enterprises if it relies on rare elements or degrades within a month.

The large‐scale application of confined catalysts in industrial hydrogen production requires innovation across several key areas. First, reducing catalyst preparation costs is essential for widespread adoption. This can be achieved by optimizing synthesis processes, using low‐cost precursor materials, and improving catalyst efficiency. Additionally, utilizing renewable resources or waste materials as precursors can further lower costs and enhance economic viability, providing strong support for promoting technology. Second, catalyst stability and durability are vital for reliable operation under harsh industrial conditions. Material modification, surface protection technologies, and novel encapsulation materials can significantly improve resistance to deactivation, corrosion, and thermal stability, thereby enhancing overall reliability. Scalable production process is equally important. Developing large‐scale production technologies, such as continuous processes and automated encapsulation, ensures uniform catalyst distribution and reaction efficiency. Using industrial‐grade equipment will further improve production efficiency and consistency, facilitating the transition to industrial application. Furthermore, in‐depth research into the confinement effect and its influence on catalytic performance, along with optimizing catalyst structure, will provide a solid foundation for advancing technology. Finally, policy support and industry collaboration are crucial for accelerating the commercialization of confined catalysts. Joint investments by governments and enterprises, industry‐academia partnerships, and the establishment of special funds will help fast‐track the development and deployment of these technologies. Through these efforts, confined catalysts are expected to be widely applied in industrial hydrogen production, contributing to the growth of the hydrogen energy industry.

### 7.3 Nanoconfinement in Electrocatalytic Water Splitting: Progress and Prospects

Significant progress has been made in the research of confined catalysts for water splitting, particularly in terms of catalytic efficiency, selectivity, and stability. As a green hydrogen production method, the efficiency of water splitting and the cost of hydrogen production are directly affected by the performance of its catalysts. Confined catalysts regulate the surface electronic structure of catalysts by restricting their reaction space, optimizing the interaction between reactants and catalysts, and significantly enhancing the catalytic reaction rate and selectivity. The advantages of the confinement effect are mainly manifested in the following aspects. First, confined catalysts can enhance the interaction between reactants and catalysts by altering the adsorption mode and electronic structure of reactant molecules, which is particularly prominent in the HER and OER of water splitting. Second, confined catalysts generally exhibit higher stability, especially under extreme reaction conditions such as high temperature, high pressure, and strong acid/alkali environments. Compared with traditional catalysts, confined catalysts can effectively avoid the aggregation of metal particles, thus reducing catalyst deactivation. These characteristics enable confined catalysts to demonstrate stronger durability and long‐term service performance in water splitting. However, the application of confined catalysts in water splitting still faces several challenges. First, the synthesis methods are complex and costly. Especially regarding the stability and performance controllability of catalysts, maintaining catalyst performance in large‐scale applications is a difficult issue. Second, although existing confined catalysts perform excellently in short‐term experiments, how to further improve the corrosion resistance and antiaging properties of catalysts during long‐term use remains a key problem to be solved. Finally, the strength of the confinement effect is closely related to the pore structure and electronic structure of the catalyst. Currently, how to precisely regulate these factors to optimize the catalytic effect remains a research hotspot.

**Figure 17:**
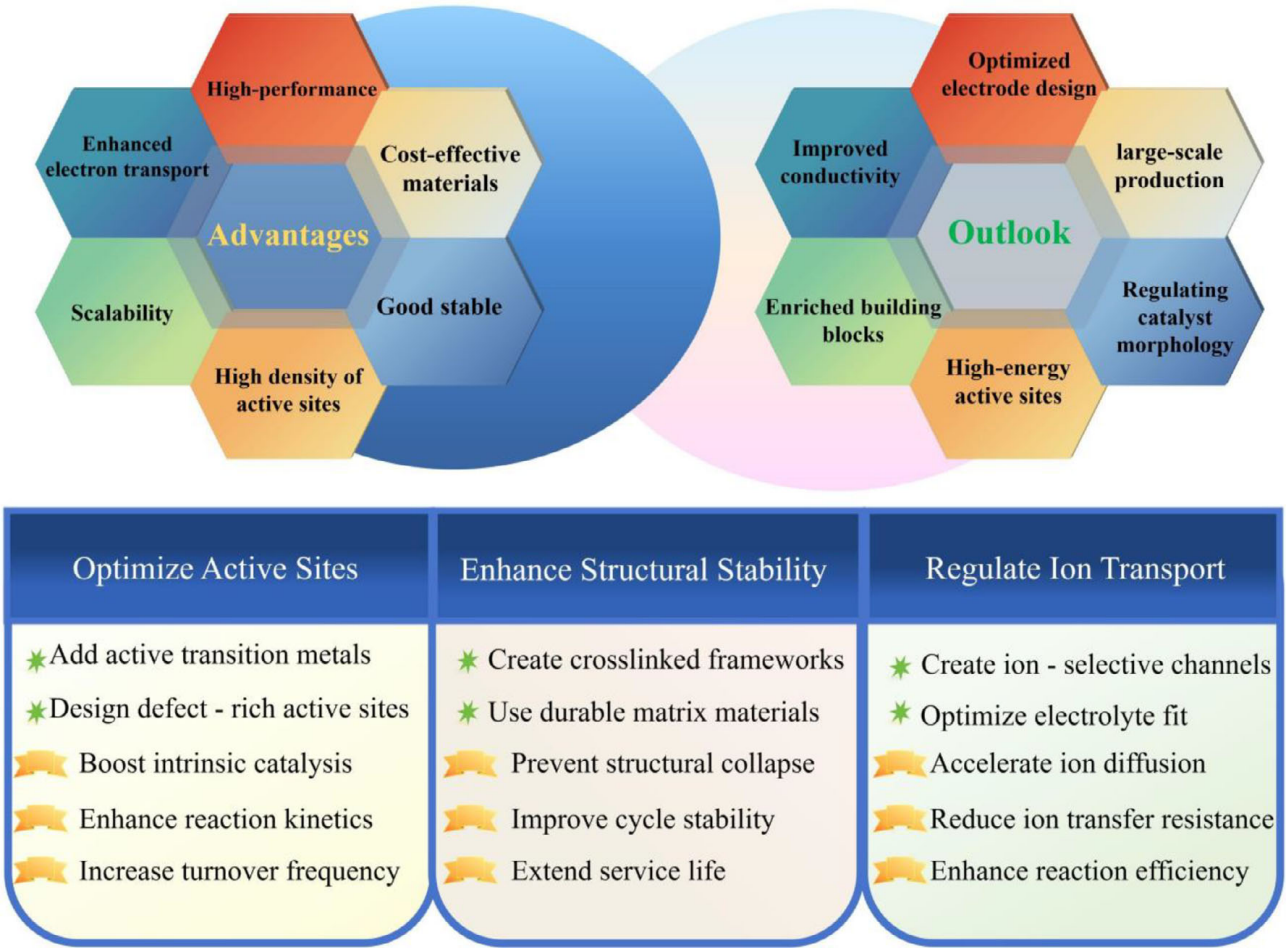
Advantages and outlook of spatial confinement electrocatalysis for water splitting, along with effective approaches applied to enhance the electrochemical performance of related electrode materials.

Despite the progress made in the application of confined catalysts in water splitting, their successful deployment in broader industrial and laboratory settings requires in‐depth exploration of material design, catalytic performance enhancement, and characterization techniques. To better illustrate the future research directions and potential, Figure 17 summarizes the advantages and development prospects of confined catalysts, identifies multiple strategies for improving catalyst performance, and provides a clear framework for future studies. The figure outlines several key strategies covering different aspects of catalyst design and catalytic reaction optimization. First, optimizing active sites‐such as through the addition of transition metals, designing defect‐rich sites, and enhancing intrinsic catalytic effects‐can significantly improve reaction kinetics and accelerate reaction rates. Second, in terms of enhancing structural stability, creating crosslinked frameworks and adopting durable matrix materials can effectively prevent the structural collapse of catalysts during reactions, thereby extending their service life. Additionally, regulating ion transport is another important approach to performance improvement: constructing ion‐selective channels and optimizing electrolyte compatibility can increase ion diffusion efficiency, reduce transport resistance, and thus enhance the overall reaction efficiency.

Figure 17 provides guidance on future catalyst design, including electrode optimization, large‐scale production, and catalyst morphology regulation. These strategies will promote the widespread industrial application of confined catalysts. Regarding large‐scale industrial hydrogen production, as discussed in the previous section, the stability and operability of confined catalysts remain key challenges. Effective optimization in synthesis methods, cost control, and long‐term maintenance of catalyst performance are essential for realizing their industrial application. Based on these strategies, future research will focus on the following five areas:

I. Development and optimization of advanced materials: Researchers will strive to develop more stable and efficient catalyst materials. Novel nanomaterials such as 2D transition metal dichalcogenides (TMDs) and perovskite materials exhibit excellent electronic conductivity and high surface activity, which can further enhance catalytic performance. Meanwhile, designing catalysts with specific pore structures enables more precise regulation of interactions between reactants and catalysts, thereby optimizing the efficiency of catalytic reactions.

II. To gain a deeper understanding of how the confinement effect influences catalyst performance, future research will increasingly rely on advanced characterization techniques. Specifically, the use of in‐situ methods will enable real‐time monitoring of structural and performance changes in catalysts during catalytic processes. Techniques such as in‐situ X‐ray diffraction, synchrotron radiation, and integrated differential phase contrast scanning transmission electron microscopy (iDPC‐STEM) are essential for observing catalyst structural dynamics and reactive interactions under practical conditions. Moreover, complementary methods like X‐ray absorption spectroscopy, mass spectrometry, and neutron scattering (CMS) offer valuable insights into ionic migration and concentration changes within catalysts, shedding light on the mechanisms that govern catalyst activity and stability. By integrating these cutting‐edge techniques, researchers can analyze the atomic‐scale mechanisms of catalytic reactions, ultimately providing crucial theoretical support for the design of more efficient and stable catalysts.

III. Utilization of more accurate DFT calculations for catalyst design optimization: With advancements in computing power, DFT calculations will play an increasingly important role in catalyst design. Through more advanced DFT calculations, researchers can accurately simulate changes in electronic structure during catalytic reactions and reveal how the confinement effect affects the electronic properties of catalysts. Advanced DFT calculations not only deepens understanding of catalytic reaction mechanisms but also provides theoretical guidance for optimized catalyst design, further accelerating catalyst screening and facilitating the discovery of high‐efficiency catalysts.

IV. Introduction of in‐situ dynamic monitoring techniques: In the future, the integration of more advanced in‐situ dynamic monitoring techniques and DFT calculations will open new avenues for catalyst research. By real‐time monitoring of dynamic changes in catalysts during water splitting, researchers can accurately capture moments of performance variation and adjust catalyst design and application strategies in a timely manner. In‐situ dynamic monitoring technology can help clarify the specific impacts of the confinement effect on catalytic reaction rate, selectivity, and stability. Additionally, dynamic monitoring can provide timely feedback for catalyst design, aiding the development of more efficient and stable catalysts.

V. Integration of artificial intelligence and machine learning: Artificial intelligence (AI) and machine learning (ML) hold broad application prospects in catalyst design. Through AI and ML algorithms, researchers can mine potential catalyst design rules from massive experimental data to predict catalyst performance and stability. AI and ML not only accelerate the catalyst optimization process but also provide data‐driven support for catalyst screening, promoting the intelligent development of catalyst design.

In summary, the application of confined catalysts in water splitting is still in a stage of rapid development. Future advancements in material innovation, characterization techniques, calculation methods, and artificial intelligence will drive their widespread application in the energy sector. With the integration of interdisciplinary technologies, confined catalysts are expected to become one of the key technologies for clean energy production in the future.

## Conflict of Interests

The authors declare no conflict of interest.

## Data Availability

The data that support the findings of this study are available in the Supporting Information of this article.
